# Kinetics, Mechanism and Theoretical Studies of Norbornene-Ethylene Alternating Copolymerization Catalyzed by Organopalladium(II) Complexes Bearing Hemilabile α-Amino–pyridine

**DOI:** 10.3390/molecules22071095

**Published:** 2017-06-30

**Authors:** Kuo-Hsuan Yu, Shou-Ling Huang, Yi-Hung Liu, Yu Wang, Shiuh-Tzung Liu, Yuan-Chung Cheng, Ya-Fan Lin, Jwu-Ting Chen

**Affiliations:** 1 Department of Chemistry, National Taiwan University, Taipei 10617, Taiwan; d96223126@ntu.edu.tw (K.-H.Y.); shouling@ntu.edu.tw (S.-L.H); yuliu@ntu.edu.tw (Y.-H.L.); wangyu@ntu.edu.tw (Y.W.); stliu@ntu.edu.tw (S.-T.L.); 2 Department of Medicinal and Applied Chemistry, Kaohsiung Medical University, Kaohsiung 80708, Taiwan

**Keywords:** Norbornene–ethylene alternating copolymerization, hemilabile, amino–pyridine, palladium, geometrical isomerism, kinetics, mechanism

## Abstract

Cationic methylpalladium complexes bearing hemilabile bidentate α-amino–pyridines can serve as effective precursors for catalytic alternating copolymerization of norbornene (N) and ethylene (E), under mild conditions. The norbornyl palladium complexes in the formula of {[RHNCH_2_(*o*-C_6_H_4_N)]Pd(C_7_H_10_Me)(NCMe)}(BF_4_) (R = *^i^*Pr (**2a**), *^t^*Bu (**2b**), Ph (**2c**), 2,6-Me_2_C_6_H_3_ (**2d**), 2,6-*^i^*Pr_2_C_6_H_3_ (**2e**)) were synthesized via single insertion of norbornene into the corresponding methylpalladium complexes **1a**–**1e**, respectively. Both square planar methyl and norbornyl palladium complexes exhibit facile equilibria of geometrical isomerization, via sterically-controlled amino decoordination–recoordination of amino–pyridine. Kinetic studies of E-insertion, N-insertion of complexes **1** and **2**, and the geometric isomerization reactions have been examined by means of VT-NMR, and found in excellent agreement with the results estimated by DFT calculations. The more facile N-insertion in the *cis*-isomers, and ready geometric isomerization, cooperatively lead to a new mechanism that accounts for the novel catalytic formation of alternating COC.

## 1. Introduction

Cyclic olefin copolymers (COC) have demonstrated wide-range applications in the industries of coating, packaging, medical equipment, etc. [1,2,3,4,5,6], because of their high T_g_, high transparency, low dielectric constants, and good biocompatibility and processability [7,8,9,10,11,12,13,14,15,16]. These physical properties are usually controlled by the compositions and the microstructures of the monomers, resulting from the design of the catalysts [17,18,19,20,21,22,23,24,25,26,27,28,29,30,31,32,33,34,35,36]. For instance, with the assistance of methylaluminoxane (MAO) [17,37,38,39,40,41,42,43,44,45,46,47,48,49,50], most of the early-transition-metal metallocene derivatives catalyzed copolymerization to afford random E–N products. The ligand steric hindrance readily impedes the norbornene insertion, and thus the PE segments are usually long. On the other hand, if the copolymerization is catalyzed by the non-metallocene titanium complexes, many examples provide an alternating microstructure for the E–N copolymer products [51,52,53,54,55,56,57,58,59,60,61,62,63].

**Scheme 1:**
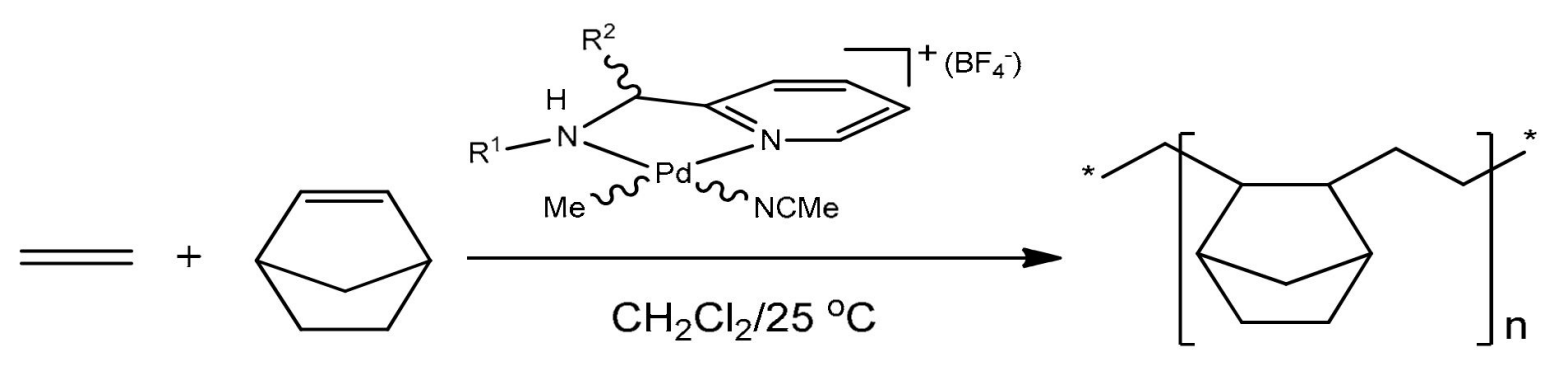
Alternating E–N Copolymerization reactions, catalyzed by {[(α-Amino–pyridine)]Pd(Me)(NCMe)]}[BF_4_].

Although late-transition metal catalysts have been employed to generate functionalized polyolefins [64,65,66,67,68,69,70,71,72], the successful examples to produce COC are still limited [73,74,75,76,77,78,79], because of the occurrence of β-H elimination on the alkyl chain. We previously reported our finding of the alternating E–N copolymerization, induced by methylpalladium(II) cations and bearing hemilabile bidentate ligand of α-amino–pyridines without the assistance of MAO (as shown in Scheme 1) [80]. The general features of the copolymerization show M_w_~10^4^, PDI~1.5, T_g_~100–120 °C, and characteristic N%~50, indicating that the COCs have a predominated alternating E–N microstructure. Furthermore, the reactivity ratios obtained from the linear Fineman–Ross plot show both r_1_ (*k*_EE_/*k*_EN_) and r_2_ (*k*_NN_/*k*_NE)_ are smaller than 1, revealing that both EE- and NN-insertion would be more difficult than EN- or NE-insertion [80]. Synthesis of the alternating copolymer is important for optical applications, since the chemical homogeneous products generate high transparency. The more the percentage compositions of alternating microstructures the copolymers contain, the higher the chemical homogeneity [73].

In order to obtain more detailed information on the structure–reactivity relationship for COC formation (in terms of the late transition metal complexe {[(α-Amino–pyridine)]Pd(Me)(NCMe)]}[BF_4_]), we carefully examined the kinetics in many catalysis-related fundamental steps. Considering E–N copolymerization may be initiated from olefin-coordination, methylpalladium (**1**) and norbornylpalladium (**2**) complexes were chosen to study both norbornene and ethylene insertion, as well as their geometric isomerization. The kinetics provide the E–N copolymerization rate constants *k*_EN_, *k*_E__E_*, k*_NN__,_ whilst *k*_NE_ was given by DFT calculations. The mechanism and the complete energy profiles for the organopalladium catalyzed alternating COC formation were also evaluated. The results establish a comprehensive mechanistic picture that may account for the alternating COC microstructures, caused by the {[(α-Amino–pyridine)]Pd(Me)(NCMe)]}[BF_4_] system.

## 2. Results and Discussion

### 2.1. Synthesis and Characterization of Norbornyl Palladium Complexes

**Scheme 2:**
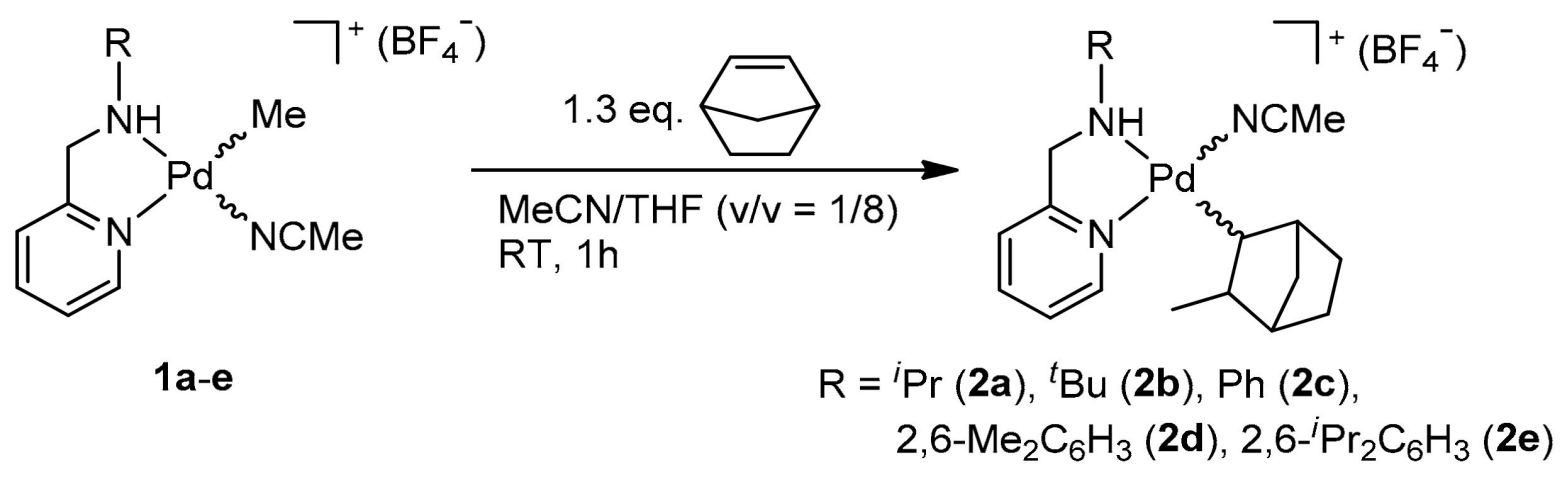
Synthesis of Norbornylpalladium Complexes.

The norbornylpalladium complexes {[RHNCH_2_(*o*-C_6_H_4_N)]Pd(C_7_H_10_Me)(NCMe)}(BF_4_)(R = *^i^*Pr (**2a**), *^t^*Bu (**2b**), Ph (**2c**), 2,6-Me_2_C_6_H_3_ (**2d**), 2,6-*^i^*Pr_2_C_6_H_3_ (**2e**)) were synthesized from the reaction of the corresponding methylpalladium complexes **1a**–**e** and 1.3 molar equivalents of norbornene, to achieve satisfactory yields in MeCN/THF (*v*/*v* = 1/8) (Scheme 2). The weakly donating solvents could efficiently suppress successive norbornene-insertion [81].

**Figure 1:**
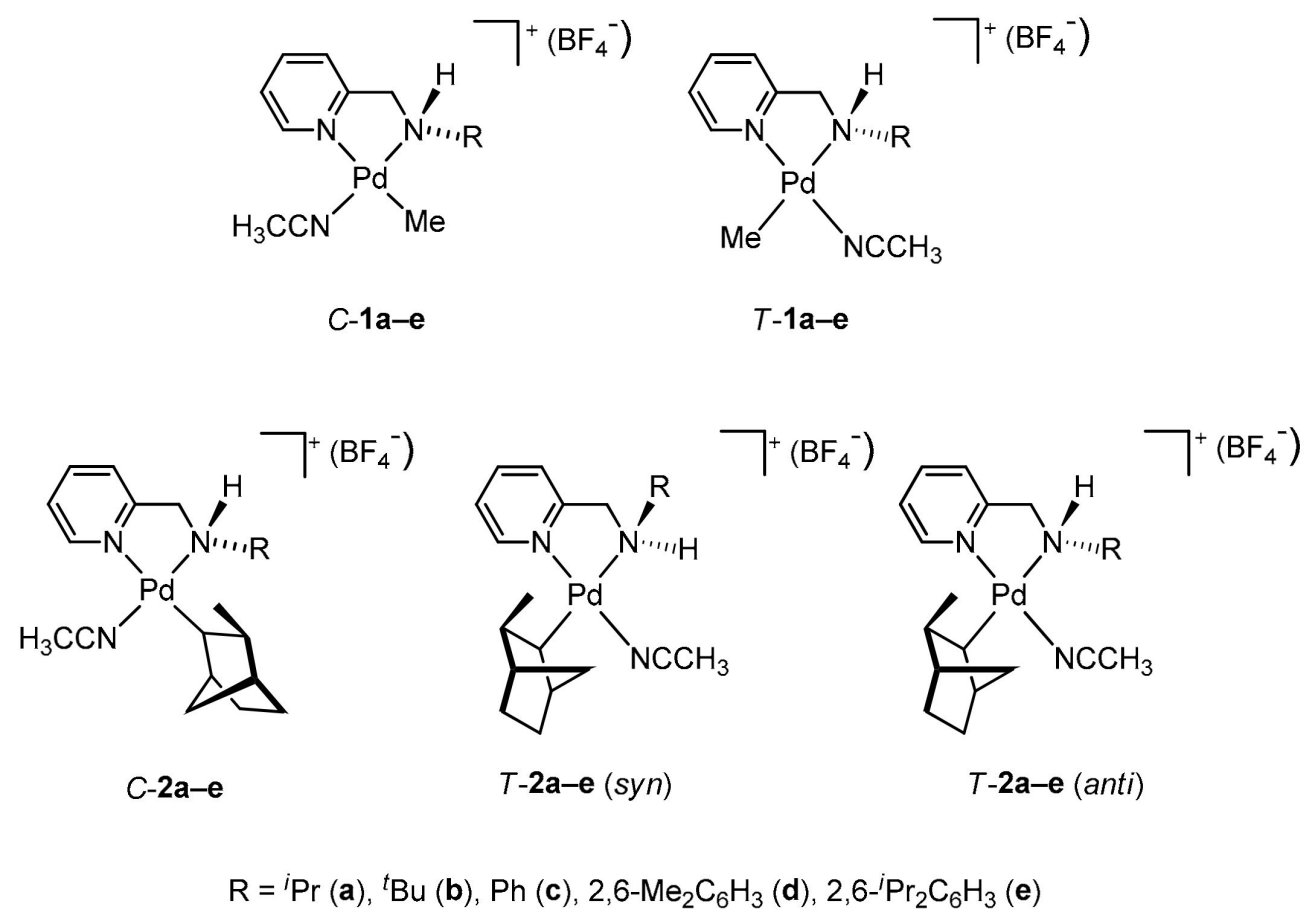
Isomers of complexes **1a**–**e** and **2a**–**e**.

Notably, the norbornylpalladium complexes, synthesized via single N-insertion into a catalytically active species, in a process of polyolefin formation, are rare [81]. Only a few examples had been achieved either by introducing a functionalized alkyl on the olefin [82,83,84,85,86,87,88], or through a neutral palladium species that showed relatively lower affinity to the olefins [89,90,91]. The NMR data measured in CDCl_3_ show that two diastereomers, exclusively in *trans*-diastereomers (*T*-form), are identified for **2a**–**b**, according to the NOE cross-peaks between the norbornyl and *ortho*-H of pyridine in the NOESY spectra. However, in the cases of **2c**–**e**, the *cis*-diastereomers (*C*-form) in low yields are also detected, as listed in Table 1. Herein, *T*-geometrical isomers are designated according to the *trans*-disposition between pyridine and MeCN (*vide infra*); and, the *C*-form for such ligands that are in the *cis* configuration (Figure 1). The selectivity of *T*-form in **2a**,**b** is ascribed to the steric hindrance between the amino substituent and the bulky norbornyl group. Such a factor turns out to be critical to the kinetic and thermodynamic selectivity (*vide infra*). The formation of small amounts of the *C*-form in **2c**–**e** is allowed, presumably due to the constraint of free rotation (due to the amino aryl substituents).

The major diastereomer for **2a**,**b** is in *syn*-configuration, in which the methyl on norbornyl and the amino substituent are at the same side, with respect to the coordination plane (Figure 1). The NOESY spectra also show the NOE cross-peaks between these two alkyl groups. The pyridinyl *o*-H signal of *anti*-configuration for **2a**,**b** is found more downfield than in the *syn*-configuration (**2a**: *anti*/*syn* = 8.42/8.32 ppm; **2b**: *anti*/*syn* = 8.39/8.29 ppm). Accordingly, the relative yields of *syn*/*anti-*products for **2c**–**2e** are evaluated.

**Figure 2:**
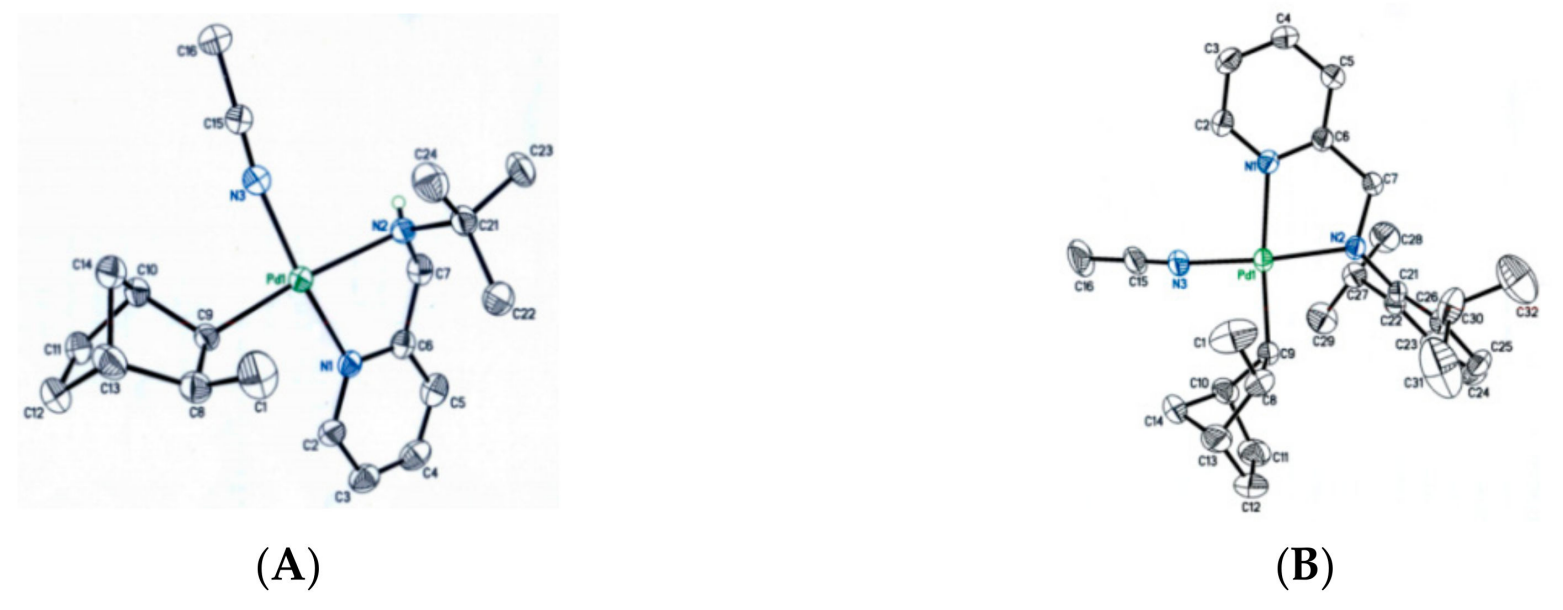
ORTEP drawings of (**A**) {[*^t^*BuHNCH_2_(*o*-C_6_H_4_N)]Pd[(C_7_H_10_)Me](NCMe)}(BF_4_) (*T*-**2b**); (**B**) {[(2,6-*^i^*Pr_2_C_6_H_3_)HNCH_2_(*o*-C_6_H_4_N)]Pd[(C_7_H_10_)Me](NCMe)}(BF_4_)(*C*-**2e**); all H-atoms are omitted for clarity.

The crystallographic analyses provide unequivocal evidence for the molecular structures of these geometrical isomers. The single crystals of *T*-**2b** and *C-***2e** were grown from co-solvents of Et_2_O/MeCN/CH_2_Cl_2_, and their ORTEP drawings are depicted in Figure 2. The collection of *C*-**2e**, which is of the kinetic product from N-insertion into **1e**, is probably due to ready decomposition of the thermodynamically stable *T*-**2e**, via C-H activation at *^i^*Pr_2_C_6_H_3_ [92,93]. The bond lengths of Pd-N1(sp^2^), Pd-N2(sp^3^), and Pd-N3(sp) of **2b**,**e** are comparable with the data of **1a**–**e** [80]. All structural parameters collected in Table S1 are in agreement with the literature data [89,90,91].

### 2.2. Geometrical Isomerisation of Norbornylpalladium Complex

**Figure 3:**
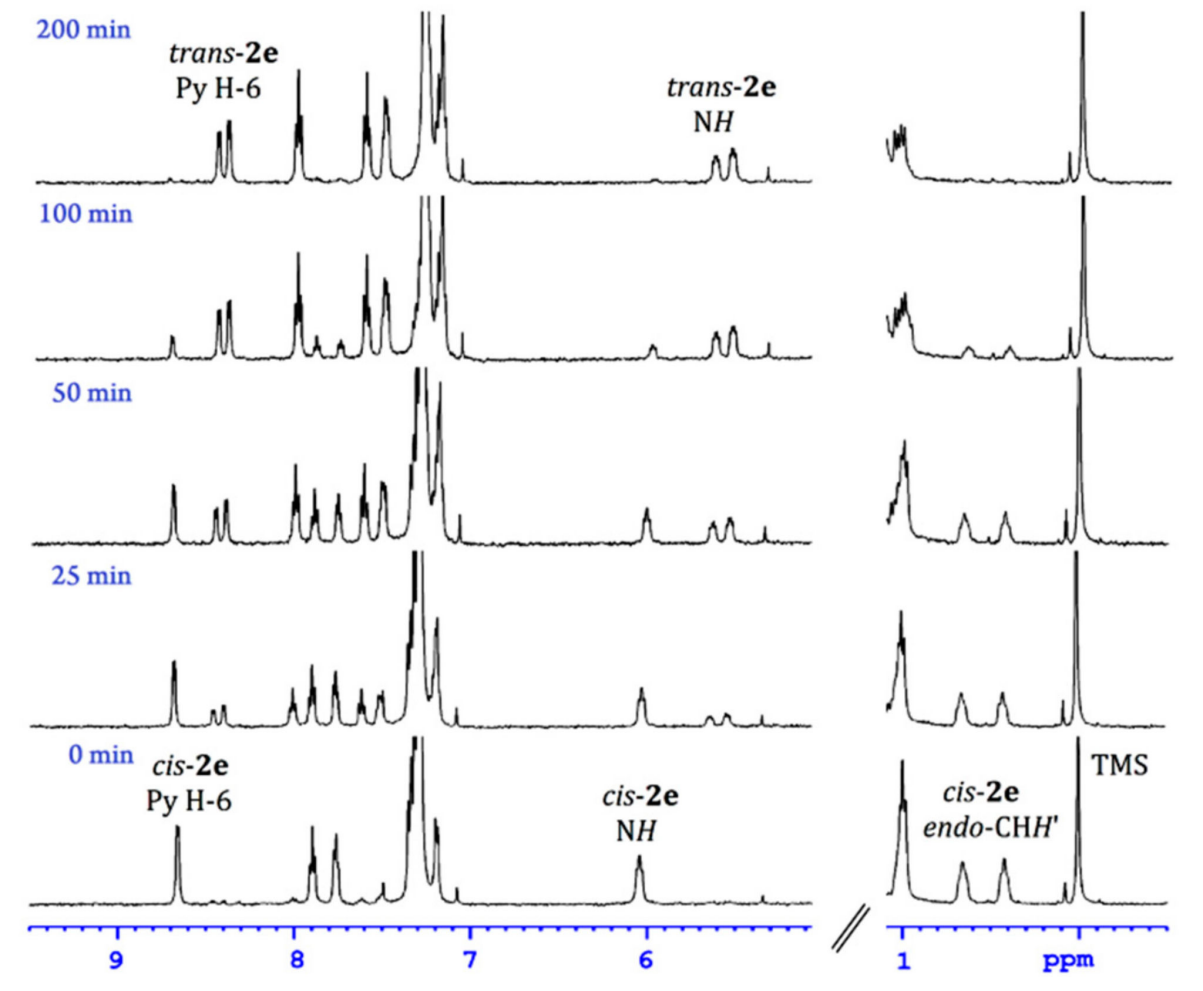
Time-resolved ^1^H-NMR spectra for geometrical isomerization of *C*-**2e** to *T*-**2e**, at 253 K in CDCl_3_.

Dissolving the crystal of *C*-**2e** in CDCl_3_ in ambient conditions resulted in transformation into *T*-**2e**, with the T/C ratio of 94/6. The equilibrium constant (K_eq_) is estimated as 15.7 (*T* over *C*). The value of ΔG^0^ at 298 K is thus −1.6 kcal/mol, calculated from K_eq_. The time-resolved spectra of such an isomerization reaction were monitored by variable-temperature ^1^H-NMR technique. The changes of pyridine(*o*-H), NH, and the norbornyl *endo*-CH_2_ signals show the formation of two diastereomers (Figure 3). The chemical shifts for the *endo*-CH_2_ of the norbornyl group in *C*-**2e** are much more upfield than in *T*-**2e**, presumably caused by the ring current from the amino aryl. Kinetic analysis showed that this isomerization reaction gives first-order rate constants 5.48 × 10^−5^–5.69 × 10^−3^ s^−1^, at 248–273 K. The differences of activation enthalpy (ΔH^‡^_isom_), Gibbs energy (ΔG^‡^_isom_), and entropy (ΔS^‡^_isom_), derived from the linear Eyring relationship, are 21.5 kcal/mol, 18.7 kcal/mol, and 9.5 cal/mol·K, respectively (Figure S5).

**Scheme 3:**
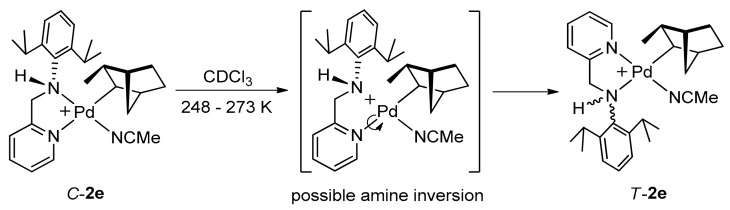
Isomerization reaction mechanism of *C*-**2e**.

The small positive value of ΔS^‡^_isom_ suggests that the geometrical isomerization of **2e** may undergo intramolecular amine decoordination–recoordination, along with amine inversion, leading to enantioisomeric exchange (Scheme 3) (although the solvent-assisted associative pathway may also be possible) [94]. The steric repulsion between the amino substituent and the norbornyl group significantly destabilizes the ground state of *C*-**2e**, and drives the amine group dissociation. Such a mechanism is also supported by the diastereotopic H-exchange of backbone methylene, in prior work of similar derivatives [90].

### 2.3. Kinetics of Norbornene Insertion Reactions in Organopalladium(II) Complexes

**Scheme 4:**
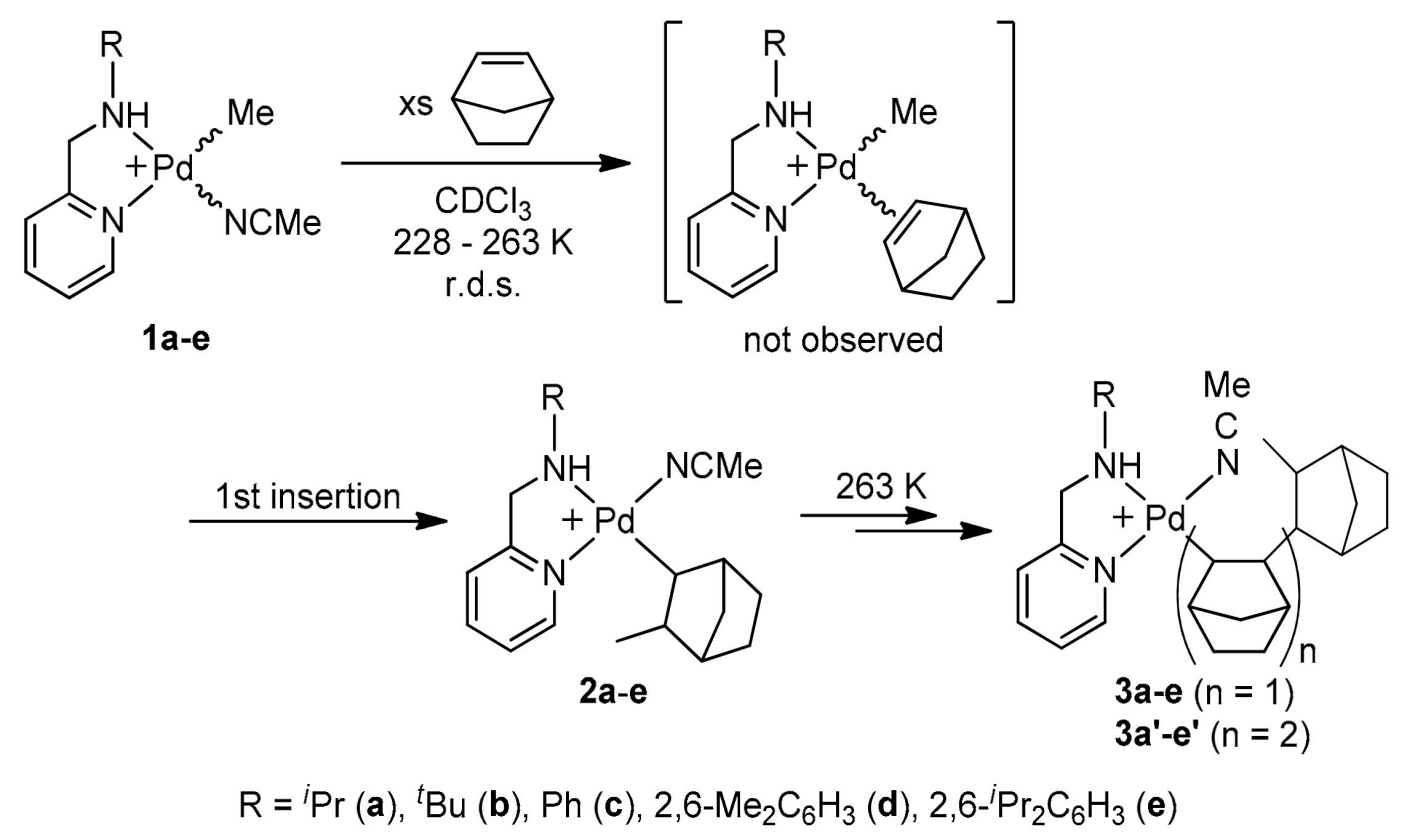
Reactions of successive N-Insertion in **1a**–**e**.

Treatment of **1a**–**e** with excess norbornene gave rise to the formation of polyads of norbornyl units, via successive norbornene insertions. To our surprise, all products are in *T*-form, as displayed in Scheme 4. Besides, the product of tetra-ads (*n* = 3) was absent upon the reaction course. If the reactions were carried out at 228–263 °C, the products **2a**–**e** were respectively produced first, and the multi-*N*-inserted products **3a**–**e** (*n* = 1), such as diads and triads **3a’**–**e’** (*n* = 2), were ensuing. In addition, the norbornene-coordinated species and free MeCN were not observed, indicating that the norbornene coordination, instead of migratory insertion, may be rate-determining (Scheme 4). The fact suggests that this case is very different from the reactions of alternating E–N copolymerization, catalyzed by bis(pyrrolide–imine)titanium, in which the olefin-insertion steps are rate-determining [23].

The kinetic studies on the reactions of the first N-insertion for **1a**–**e** were further examined with use of ^1^H VT-NMR in a pseudo-first-order manner. As listed in Table 2, entries 1–5 show the effect of the amino substituents on the reactivity at 263 K. In entries 1–3, only the rate constants for the *T*-form (*k*_obs_*^T^*) were evaluated, since the *C*-isomer was consumed under the conditions before the 1st measurement. An attempt to record *k*_obs_*^c^* in the condition of lower norbornene concentration failed, because the reaction would proceed too slowly to be monitored. Complex **1b** is significantly slower toward norbornene insertion than the runs of other analogous complexes. The observation of the smaller *k*_obs_*^T^* value of **1b** is probably because of the bulky *^t^*Bu substituent on the amine group, which would hinder the norbornene coordination, and thus retard the norbornene-insertion rate.

It is worth noting that in entry 4 the *k*_obs_*^C^* is 6.1 fold faster than *k*_obs_*^T^*. Similarly, the *k*_obs_*^C^* are 46 and 32 folds of *k*_obs_*^T^* in entries 11 and 12, respectively. Moreover, if the reactions were carried out in temperatures lower than 243 K (entries 13–18), only the consumption of the *C-*isomer was observed. The facts indicate that in such low temperatures, no *T*-to-*C* isomerization occurs, and the reaction of norbornene-insertion only takes place on the *C*-isomer. This is an unprecedented example, of which one geometrical isomer in square-planar configuration shows substantially more facile reactivity than another toward the fundamental reactions of norbornene insertion [85,95].

To shed further light on the kinetics of the norbornene insertion, we chose **1e** to determine the rate laws. The plots of the pseudo-first-order rate constants, against the concentration of norbornene ([N]) in Figure S24, show that *k*_obs_*^C^* is directly proportional to [N] (Entries 15–17), while *k*_obs_*^T^*[N] relationship is linear with a non-zero intercept, which represents the rate constant of *T*-to-*C* isomerization, *k*_isom_. (Entries 5–9). Therefore, the rate law for the norbornene-insertion reactions can be written as Equations (1) and (2), indicating that in *C*-**1e** the rate of N-insertion is much faster than that of isomerization (whereas in *T*-**1e**, the two reactions compete with each other).

(1) $$-\frac{d\left[C-1e\right]}{\mathrm{dt}}=k_{\mathrm{obs}}^{C-1e}\left[C-1e\right]=k_{N}^{C-1e}\left[N\right]\left[C-1e\right]$$

(2) $$-\frac{d\left[T-1e\right]}{\mathrm{dt}}=k_{\mathrm{obs}}^{T-1e}\left[T-1e\right]=(k_{N}^{C-1e}\left[N\right]+k_{\mathrm{isom}})\left[T-1e\right]$$

Indeed, the rate constants *k*_N_*^C^*^-**1e**^ (Entry 10) and *k*_N_*^T^*^-**1e**^ at 263 K were evaluated as 3.06 × 10^−^^1^ and 2.14 × 10^−^^3^ M^−1^s^−1^, and the rate constants of *T*-to-*C* and *C*-to-*T* isomerization (*k*_isom_ and *k*_isom_^−1^) were estimated as 4.60 × 10^−4^ and 8.48 × 10^−4^ s^−1^ (Data were obtained from the intercept of the *k*_obs_*^T^*-[N] linear and the equilibrium constant of isomerization (K_C/T_ = 0.54 at 263 K)). Furthermore, ΔH^‡^_N_ and ΔS^‡^_N_ for the N-insertion reaction of *C*-**1e** were obtained from the Eyring plot, as 13.8 kcal/mol and −8.9 cal/mol·K, respectively (entries 11–14 and 18, Figure S25). The small negative value of ΔS^‡^_N_ agrees with an intermediate A-pathway [96]. One may assert that the rate-determining step of norbornene-coordination tends to be critically dependent on the steric hindrance between the amino substituent and the norbornene. Such a stress may be released via either geometrical isomerization or migratory N-insertion.

If the N-insertion is much more favored in the *C*-form, the problem then arises: why were no PN products observed? A reasonable assumption is that the steric hindrance would be increased by the increasing numbers of inserted norbornene, inhibiting the coordination step. Accordingly, we investigated the 2nd and the 3rd insertion rates for **1a** at 263 K, and found they are ranked in the order of 1st > 2nd ~3rd >> 4th (Figure S26 and Table S9).

The norbornene-insertion reactions of **1** and **2** are worthy to note in several aspects: (1) the N-coordination step is rate-determining, and the N-insertion reactions are favored in the *cis* isomers; (2) the products favor the *trans* isomers, and the *C*–*T* isomerizations take place owing to the steric effects in the hemilabile ligands; and (3) the geometrical isomerization is capable of winning the competition over the successive N-insertion, thus facilitating the alternating N–E-insertion.

### 2.4. Kinetics of Ethylene Insertion Reactions in Organopalladium(II) Complexes

**Scheme 5:**
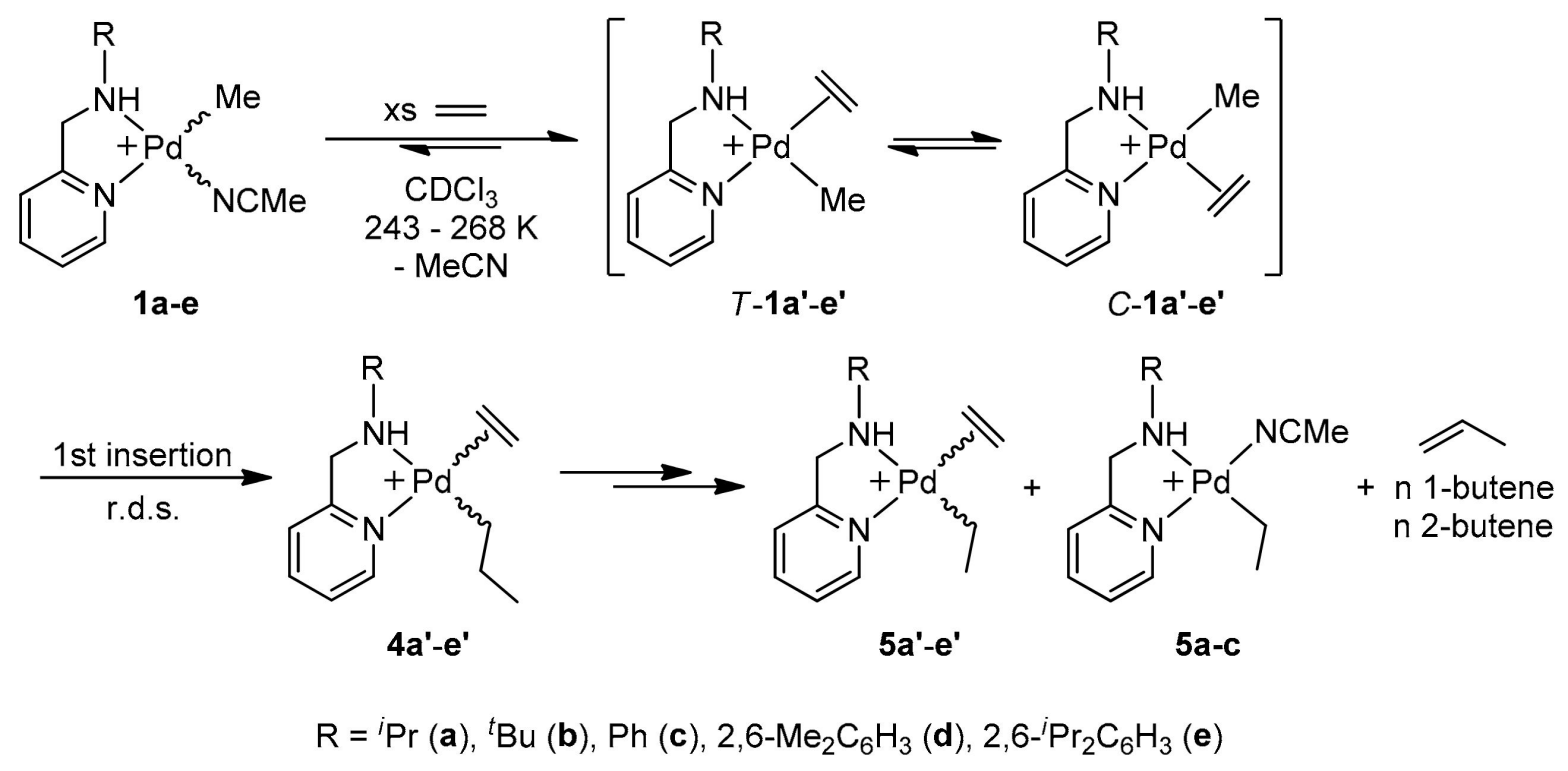
E-Insertion Reactions in complexes **1a**–**e**.

In the presence of excess ethylene, MeCN in **1a**–**e** were readily replaced by ethylene to form *T*/*C*-{[RHNCH_2_(*o*-C_6_H_4_N)]Pd(Me)(C_2_H_4_)}(BF_4_) (**1a’**–**e’**), followed by the E-insertion and an ensuing E-coordination to give *T*/*C*-{[RHNCH_2_(*o*-C_6_H_4_N)]Pd(C_3_H_7_)(C_2_H_4_)}BF_4_) (**4a’**–**e’**), as shown in Scheme 5.

Consequently, ethyl(ethylene)palladium in the form of *T*/*C*-{[RHNCH_2_(*o*-C_6_H_4_N)]Pd(Et)(C_2_H_4_)}-(BF_4_) (**5a’**–**e’**), along with the propene and butenes of chain-transfer products, were detected [97]. *T*-{[RHNCH_2_(*o*-C_6_H_4_N)]Pd(Et)(MeCN)}(BF_4_) (**5a**–**c**), with respective yields of 29%, 50%, and 24%, were also obtained. The absence of the *C*-**5a**–**c** is presumably due to the conformational steric hindrance between the amino substituent and the ethyl ligand [80,98]. *T*-forms are of the more favored configurations in **1a’**–**d’** and **5a’**–**e’**, again, ascribed to the steric effect (Table 3). The E-coordinated complexes appear to undergo geometrical isomerization more facile than that in **2a**–**e**, according to the broadened or coalescent Pd-alkyl signals for **1b’**–**e’**, **5a’**–**b’**, and **5d’**–**e’** found at 263 K.

The kinetic data in Table 4 were collected by monitoring the Me-Pd signals of **1a**–**e** under pseudo-first-order conditions, with excess of [E]. The values of *k*_E_ were measured from the transformations of **1a’**–**e’** to **4a’**–**e’**. Unlike in the processes of N-insertion, the migratory E-insertion in **1’** is comparable between two geometrical isomers. On the other hand, the differences of *k*_E_ are limited in terms of ligand variation (Entries 1–4 and 6).

In entries 6–8, the values of *k*_E_ for **1e** appear to be independent on [E], suggesting the rate-determining step ought to be the step of E-insertion [93,99,100,101,102]. Moreover, the Eyring analyses provide the activation parameters of the E-insertion in **1e**, as 17.3 kcal/mol for ΔH^‡^_E_ and −7.4 cal/mol·K for ΔS^‡^_E_, that are comparable to those for N-insertion in *C*-**1e**. That is probably also an accidental factor for the occurrence of the E–N alternating copolymerization.

In summary for the E-insertion reactions of **1a**–**e**, the E-coordination to **1**, as well as the geometrical isomerization of the ethylene-coordinated species, has to be ready. The successive E-insertions are quenched by β-elimination, but may be accelerated at high [E]. It is assumed that a hydridopalladium species may be formed by chain transfer, and serves as the actual catalytically active resting state. Overall, the E-insertions are less critical than the N-insertions in {[RHNCH_2_(*o*-C_6_H_4_N)]Pd(Me)(NCMe)]}[BF_4_] catalyzed formation of alternating COC.

### 2.5. DFT Approach for E–N Copolymerization

**Chart 1:**
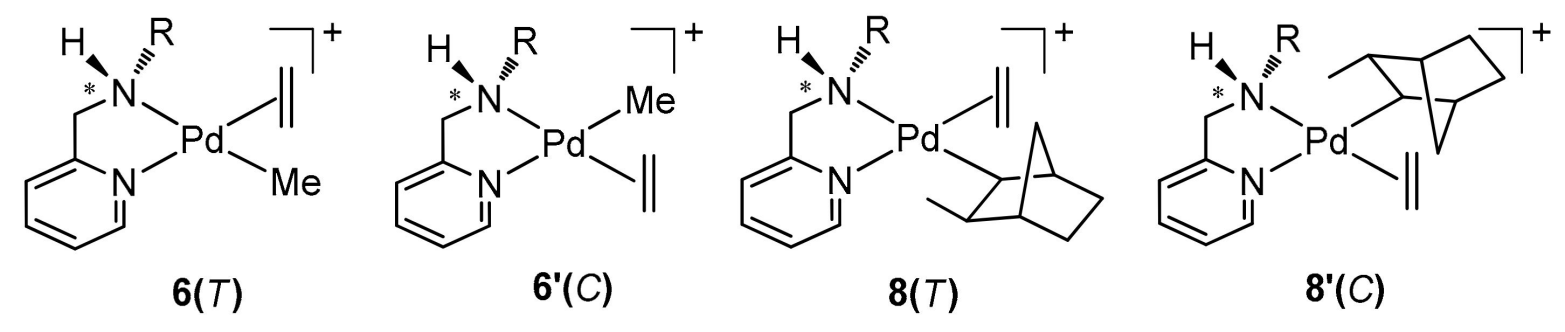
Structures of resting states. (R = 2,6-*^i^*Pr_2_C_6_H_3_).

Kinetic studies for N-insertion and E-insertion reactions provide the evaluations of *k*_N_ and *k*_E_, respectively, which can model the *k*_EN_, *k*_NN_, and *k*_EE_ for E–N copolymerization. Nevertheless, the ^1^H-NMR spectra for the E-insertion reactions of **2a**–**e** are too complicated to have successful kinetic analysis. In order to gain the missing information for *k*_NE_, and to have a comprehensive mechanistic picture for such alternating E–N copolymerization, the gas-phase DFT studies were applied (The chirality of amine is fixed as *R* form not only for easier comparison but also due to the enantiomers giving almost the same energies in preliminary calculations. The energies for *syn*- and *anti*-isomers of norbornyl complex, showing similar energies, were also calculated from energy scan of rotating the Pd–C bond on norbornyl ligand). The complex **1e** was chosen for the calculations of the reaction energy profile, in which the fundamental steps were concerned. Considering the replacement of E for MeCN in organopalladium(II) complexes is feasible and complete (as shown in Scheme 5), the E-coordinated species **6**/**6’** and **8**/**8’**, as illustrated in Chart 1 below, are proposed as the resting states for the calculations.

#### 2.5.1. *C*–*T* Isomerization

**Scheme 6:**
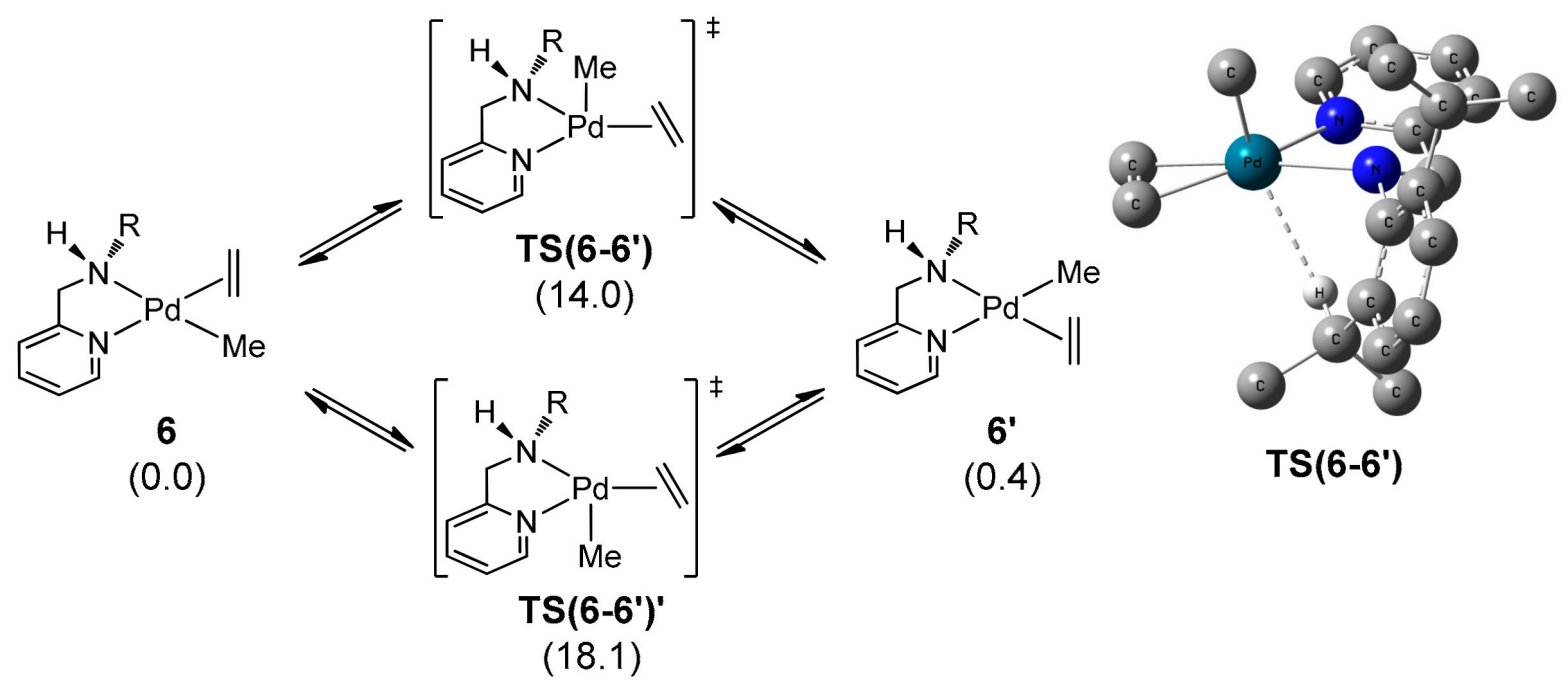
*C*–*T* isomerization via distorted tetrahedral species for norbornyl complexes (E + ZPE, kcal/mol). (R = 2,6-*^i^*Pr_2_C_6_H_3_).

As aforementioned, the isomerization reaction of **1e’** is more facile than those of **1e** and **2e**, suggesting that the isomerizations with E-coordinate and MeCN-coordinate organopalldium species might not be the same. We calculated several kinds of pathways such as A-type [103,104,105], D-type [106], amine-dissociation, Berry pseudorotation [107,108], and direct geometry change [109,110,111,112,113], and found that the last one is the most energy-favored. Rotation of the dihedral angle of N_py_–N_am_–Pd–C_Me_ affords pseudo penta-coordinate transition states **TS(6**–**6’)** and **TS(6**–**6’)’**, with barriers of 14.0 and 18.1 kcal/mol, respectively (Scheme 6). **TS(6**–**6’)** has a weak δ-agostic interaction of the *^i^*Pr group, with the distance Pd–H = 2.5286 Å, shorter than the sum of Van der Waals radii (2.72 Å).

On the other hand, the amine decoordination–recoordination pathway has a barrier of 21.7 kcal/mol, in agreement with the case of **2e**. The results show *C*–*T* isomerization via pseudo penta-coordinate geometry is much lower in energy than via the amine-dissociation pathway. Accordingly, the isomerization mechanisms of the E-bound and MeCN-bond complexes are different. The former may undergo an unusual geometry change, and the later may likely go through amine dissociation. The difference successfully accounts for the experimental results that isomerization of E-bound species are more rapid than the MeCN-bound ones. Likewise, the norboryl species **8** requires 15.1 kcal/mol to overcome activation energy to form **8’**, via a similar pathway of geometry change.

#### 2.5.2. Ethylene Insertion into (C_2_H_4_)Pd-Me

**Figure 4:**
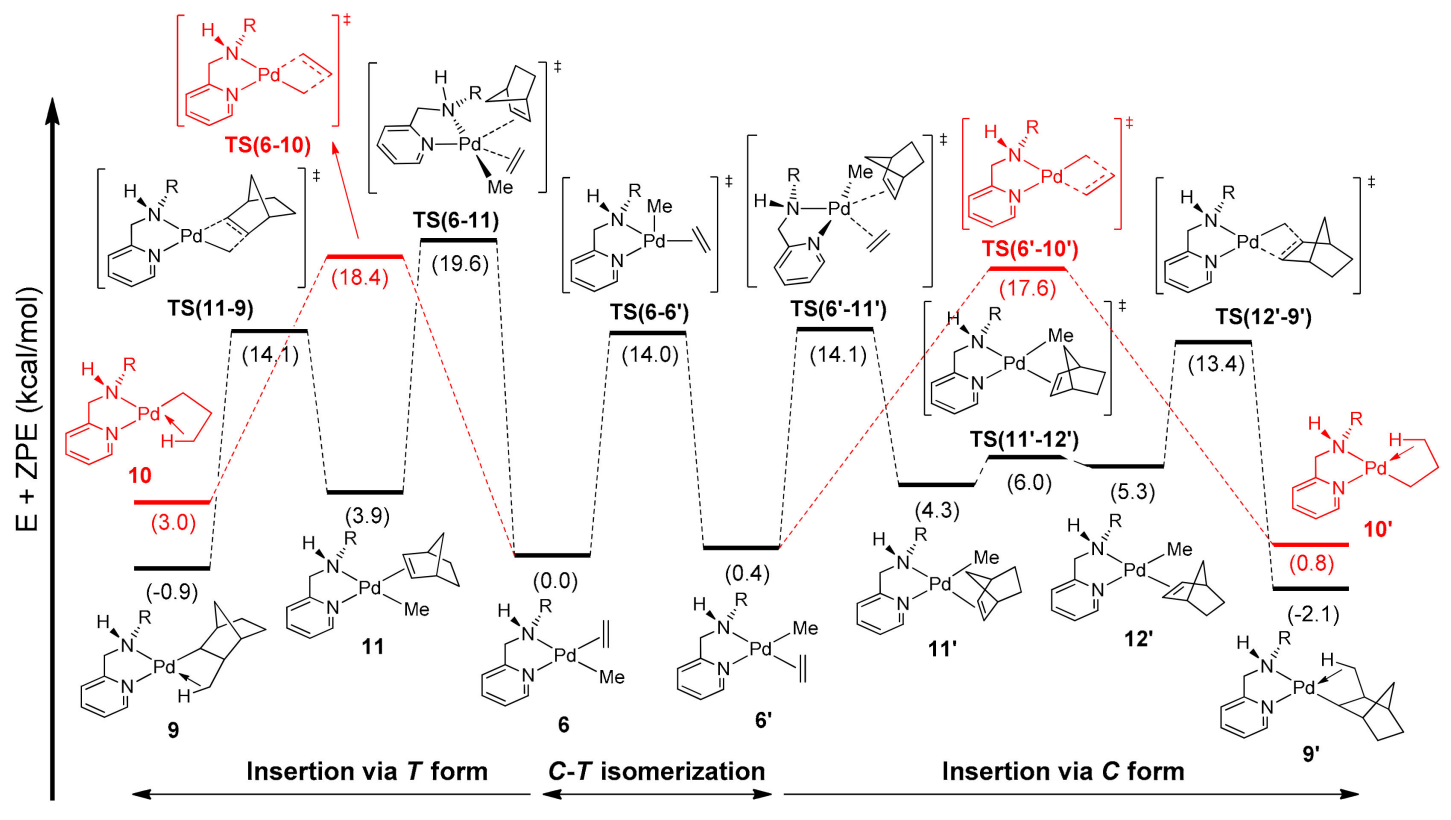
Energy profile for isomerization, ethylene, and norbornene propagation reaction of **6** and **6’** (E + ZPE, kcal/mol). (R = 2,6-*^i^*Pr_2_C_6_H_3_).

The calculations for ethylene insertion reactions started from the E-bound methyl palladium species **6** and **6’**. As depicted in red in Figure 4, the endothermic insertion steps proceed with activation energies of 18.4 and 17.2 kcal/mol, respectively, to give γ-agostic species **10** and **10’**. The former data is in excellent agreement with the kinetic data for **1e’** (17.3 kcal/mol). IRC scan led to local minima **6** and **6’**, and no in-plane π-complex could be found. The mechanism of ethylene insertion is also supported by the previous studies [93,99,100,101,102,114,115,116].

#### 2.5.3. Norbornene Insertion into (C_2_H_4_)Pd-Me.

The norbornene insertion reactions of **6** and **6’** start to approach Pd from the side, above the coordination plane of **6**/**6’**. The transition states **TS(6**–**11)**/**TS(6’**–**11’)** were found to be in the TBP structure. IRC calculations showed that **TS(6**–**11)** and **TS(6’**–**11’)** would respectively form the N-bound π-products **11** and **11’**, first by the olefin replacement. The activation barriers are 19.6 (via *T*-form) and 13.7 (via *C*-form) kcal/mol.

Although both substitution reactions are endothermic by 3.9 kcal/mol, the conformation of norbornene in **11** and **11’** are different. In **11**, the bridge-head CH_2_ moiety tilts away from pyridine, while the CH_2_ directs toward the pyridine in **11’**. In consequence, insertion directly takes place from **11** to form the γ-agostic product **9** by 10.2 kcal/mol barrier. In contrast, **11’** was turned to **12’** by rotating norbornene, then norbornene insertion followed to give **9’**. The rotational motion and insertion barriers are calculated as 1.7 and 8.1 kcal/mol, respectively. The reactions from **9** to **11** and **9’** to **11’** are thus exothermic by 4.8 and 7.4 kcal/mol, respectively (Figure 4).

Overall, the rate-determining step of the norbornene insertions should be the substitution of ethylene by norbornene, via a transition state of N-association (**TS(6**–**11)**/**TS(6’**–**11’))** from the E-bound species. The reaction via *C*-form requires a smaller energy barrier, and thus proceeds faster, consistent with the kinetic results of **1e**. Again, the activation energy between **6’** and **TS(6’**–**11’)**, 14.1 kcal/mol, meets the kinetic result of *C*-**1e**, 13.8 kcal/mol very well.

**Scheme 7:**
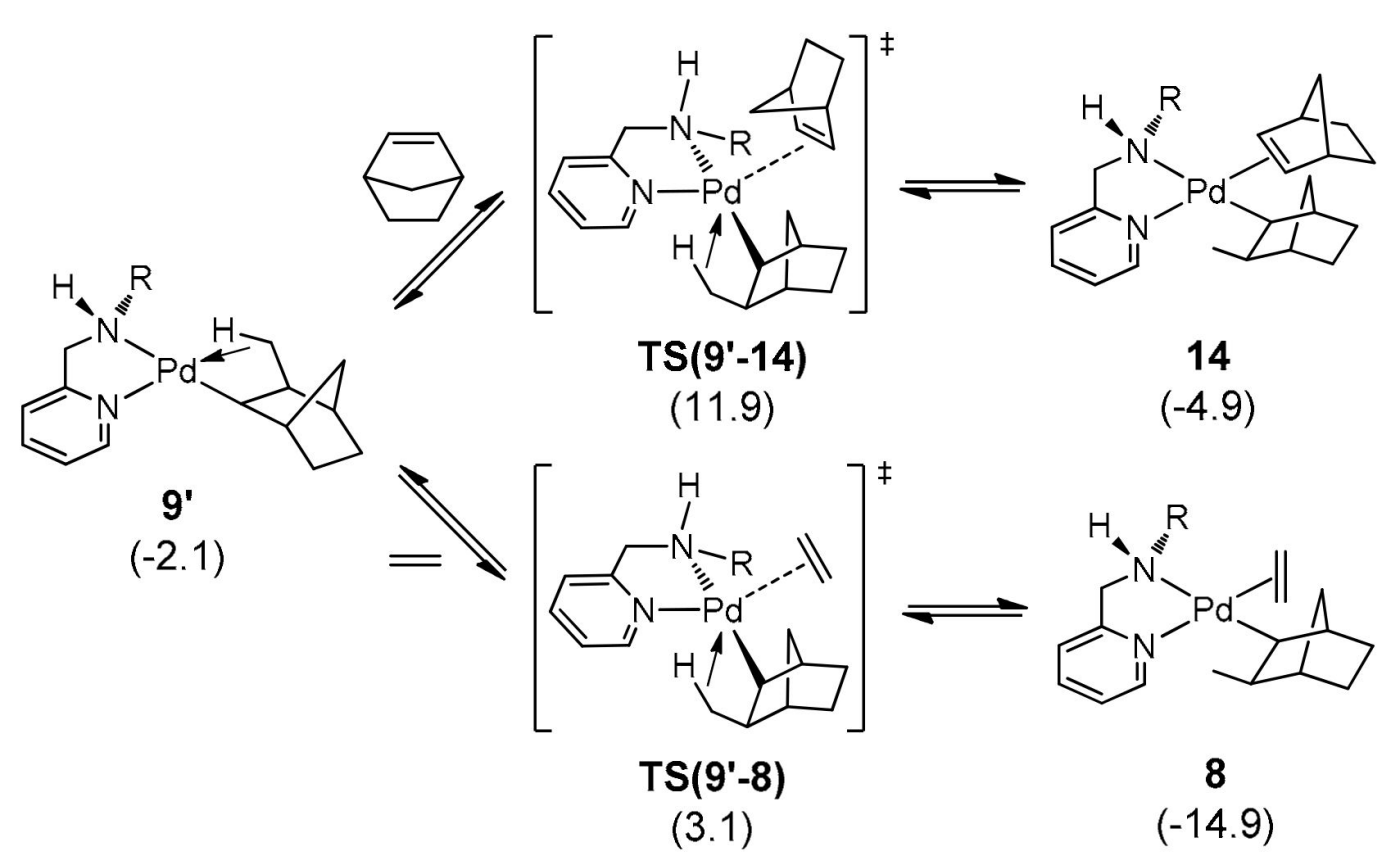
Coordination pathway of ethylene or norbornene for γ-agostic intermediate **9’** (E + ZPE, kcal/mol). (R = 2,6-*^i^*Pr_2_C_6_H_3_).

The subsequent ethylene coordination to **9’** is calculated as an exothermic reaction by 12.8 kcal/mol, and the corresponding activation energy is 5.2 kcal/mol (Scheme 7). In contrast, the energy difference and activation energy for norbornene coordination to **9’** were calculated as −2.8 and 14.0 kcal/mol. Apparently, the process of ethylene coordination to **9’** is substantially favored after the N-insertion reaction, thereby supporting the alternating copolymerization.

#### 2.5.4. Ethylene and Norbornene Insertion into (C_2_H_4_)Pd-C_7_H_10_Me

**Figure 5:**
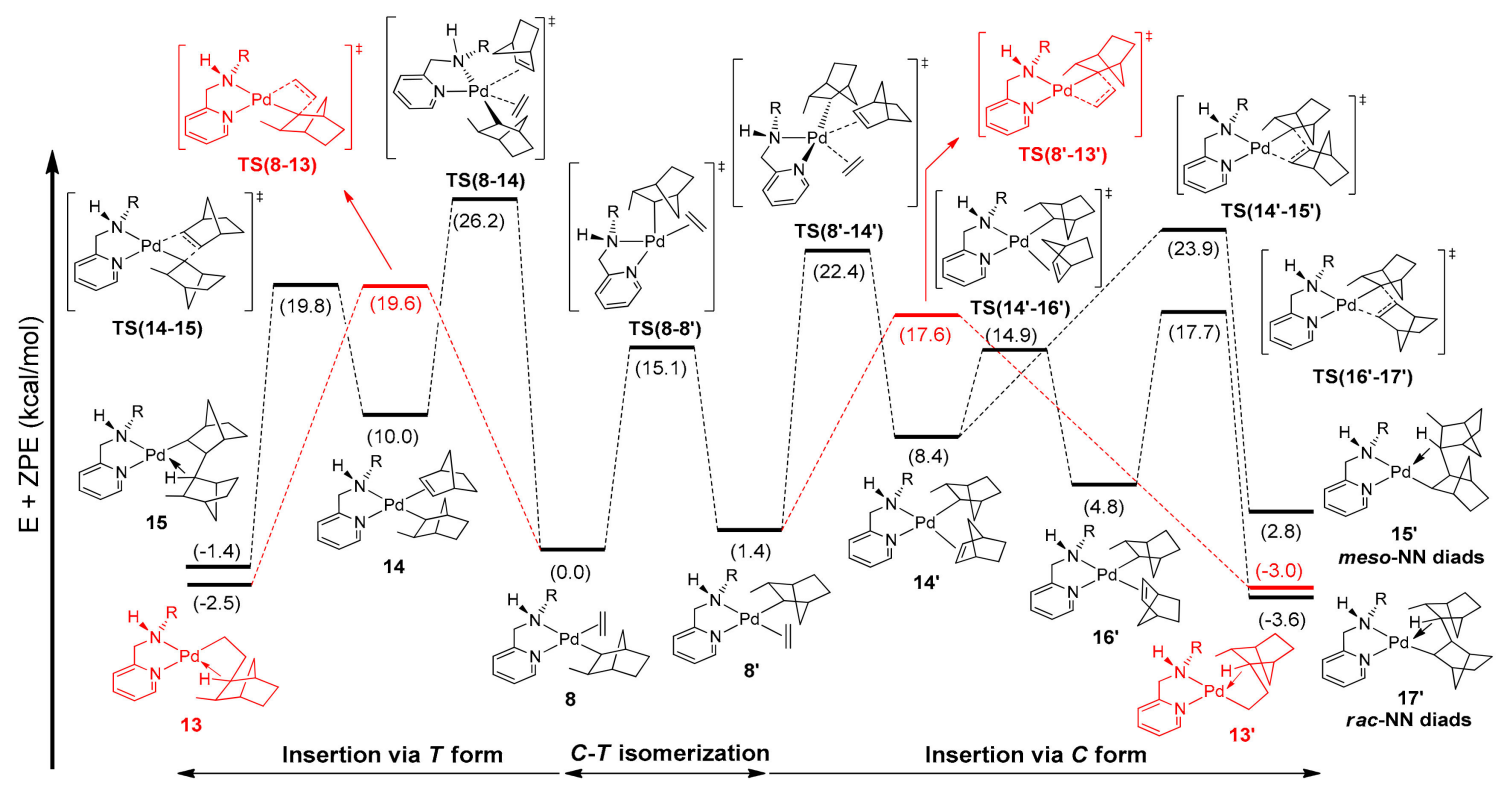
Energy profile for isomerization, ethylene and norbornene propagation reaction of **8** and **8’** (E + ZPE, kcal/mol). (R = 2,6-*^i^*Pr_2_C_6_H_3_).

The energy profiles for ethylene and norbornene insertion processes from **8** and **8’** are illustrated in Figure 5. The transition structure **TS(8’**–**13’)** in the *cis*-form is 2.0 kcal/mol lower than **TS(8**–**13)**, thus the activation barrier is 3.4 kcal/mol lower, supporting the kinetic results (red line in Figure 5). As a consequence, one may propose the thermodynamically more stable isomer **8** converts to **8’**, followed by ethylene insertion to form **13’**, confirming the alternating COC formation.

The N-insertion reactions from **8** and **8’** are found to go through the pathways similar to those started from **6** and **6’**. However, the activation energies of N-coordinating steps are 26.2 (via *T*-form) and 22.4 (via *C*-form) kcal/mol, much higher than the cases for **6** and **6’**. The process for the *C*-form still has a smaller energy barrier, although not enough to make competition with the E-insertion. The successive N-insertions appear to achieve the NN-diads in the racemic form, and also agree with the experimental results.

#### 2.5.5. Mechanism of Alternating E–N Copolymerization

**Scheme 8:**
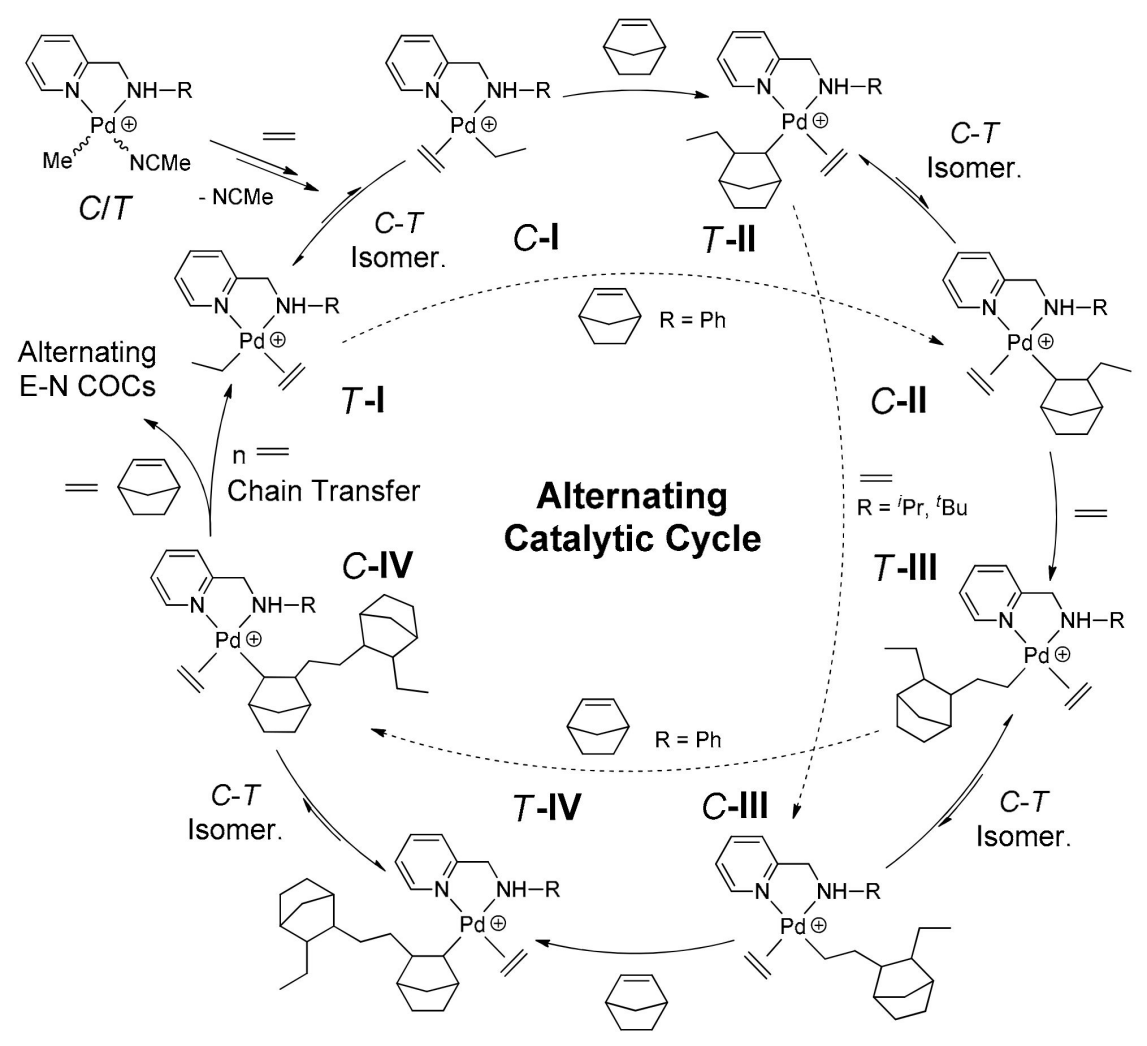
Alternating Catalytic Cycle of E–N Copolymerization. (Solid line: R = 2,6-Me_2_C_6_H_3_, 2,6-*^i^*Pr_2_C_6_H_3_; dash line: R = *^i^*Pr, *^t^*Bu, Ph).

The structure-reactivity relationship of alternating E–N copolymerization may be illustrated as the catalytic cycle, shown in Scheme 8.

The resting states of *T*-**I** and *C*-**I**, representing the E-last-inserted intermediates, are the result of chain transfer. In the major cycle, the *C*-**I** reacting with norbornene is favored to yield *T*-**II**, which prefers to isomerize to *C*-**II**. The *C*-**II** undergoes facile E-insertion to give *T*-**III**, which prefers to isomerize to *C*-**III**, then *T*-**IV** and *C*-**IV**, and so on. The E-N alternating configurations thus may be achieved.

The rapid isomerization between *T*-**I** and *C*-**I** equilibrates, but is less likely to go to *C*-**II** from *T*-**I**. In the equilibrium between *T*-**II** and *C*-**II**, the former one is predominant because of the steric hindrance between the norbornyl and amino moieties. The *T*-**II** can alternatively convert to *C*-**III** too, via E-insertion. In the case of *T*-**III**, instead of isomerizes to *C*-**III**, it may undergo E-insertion to yield *C*-**IV**. Such pathways will also achieve E–N alternating configurations.

The successive N-insertion would have a chance to take place in the *C*-**II** species to give racemic norbornene diads, particularly with high norbornene feeding. The *T*-**II** species unlikely proceeds via such a process, due to its disfavored steric effect. The successive E-insertion may also take place in the *C*-**III**. Such results were indeed observed under the conditions of high pressure of ethylene and low feeding with norbornene.

One may notice that the equilibria of geometrical isomerizations allow the catalysts to choose the more facile pathway of insertion. For the N-last-inserted species such as **II** and **IV**, the *cis*-isomers that undergo E-insertion will be predominant. And, for the E-last-inserted species as **I** and **III**, N-insertion may occur in either isomer. The control factor is mainly attributed to the steric effect.

Of course, competitions are always available when the kinetic conditions vary upon the reaction course. The formation of N-diads or even N-triad, and E-polyads or branches, had been indeed observed by ^13^C-NMR. With the N-feeding in the region of 6–79 mol%, the produced COCs containing norbornene units in 42.0–59.6% and the alternating compositions of E–N are calculated as 70.4–94.2% (Table S1) [80]. One may conclude that the alternating copolymerization is of a more general situation.

Comparing with the catalytic E–N copolymerization using diimino palladium catalysts, in which 5–80 mol % norbornene-feeding resulted in the norbornene contents are ranged in 9–60% in the COCs, the hemilabile unsymmetric amino–pyridine ligands in this study provide unprecedented examples that can afford selective isomerization-controlling olefin-insertions, and lead to high norbornene content as well as a high percentage of alternating E–N microstructure in the COCs.

The activity data, norbornene contents, and norbornene blocks, with alternating percentages in the COC products catalyzed by **1a**–**e**, are collected in Table 5. All data for **1d** and **1e** are generally comparable. Complexes **1a**,**b**,**c** show substantially lower activity; however, slightly less or nearly comparable results in the norbornene contents and alternating compositions were acquired compared to those of **1d**,**e**. The unusually high percentages of N-block for **1c** may be blamed on its relatively large *k*_N_*^T^* and *k*_NN_*^T^*.

We accordingly ascribe that the activity depends on the initialization of N-coordination, as well as the chain transfer or the termination steps. The N-block and alternating compositions depend on the propagation steps, due to N- and E-insertions. Apparently, the fundamental kinetics follow the relationship: *k*_EN_ > *k*_NE_ >> *k*_NN_, *k*_EE_, as well as the concentrations of the monomers. In addition, the critical isomerization equilibria should never be overlooked by viewing the distinct *C*/*T* ratios for the resting complexes **5’** and the precursors **1** and **2**.

## 3. Materials and Methods

### 3.1. Materials

Commercially available reagents were purchased and used without further purification unless otherwise indicated. THF, diethyl ether, dichloromethane, hexane and toluene were dried by passage through an M. Braun solvent purification system (MB-SPS) prior to use. Acetonitrile was distilled over anhydrous CaH_2_. All manipulations of air-sensitive material were performed under a nitrogen atmosphere either in a glove box or by standard Schlenk techniques. The preparation of [(N^N)PdMe(NCMe)][BF_4_] (1a–e) was described in our previous work [80].

### 3.2. Measurements

The NMR spectra were measured on a Bruker DPX-400, AVIII-400 or DMX-500 spectrometer. The corresponding frequencies for ^13^C-NMR spectra were 100.625 and 125.753 MHz, respectively. Values upfield of ^1^H- and ^13^C- data were given in *δ* (ppm) relative to chloroform in CDCl_3_ (7.24, C*H*Cl_3_; 77.0, *C*HCl_3_) or to benzene in d_6_-benzene (7.15, C_6_*H*_6_; 128.7, *C*_6_H_6_). To have good integration data, the ^13^C NMR spectra of copolymers were obtained at 100.625 MHz in CDCl_3_ or C_6_D_6_ using inverse gated proton decoupling with 30 degree pluse and 3 s delay between the pluses. The NMR probe temperature for kinetic analysis was calibrated by using methanol standard (183–273 K) before the measurement. Assignments are based on ^1^H- and ^13^C-NMR spectroscopy with COSY, NOESY and HSQC techniques. High-resolution mass spectrometric analyses were collected on a WATERS LCT Premier XE spectrometer. Elemental analysis was done on a Thermo Scientific FLASH 2000 CHNS analyzer. Gel permeation chromatography (GPC) was performed in toluene at 40 °C using a Kratos model spectroflow 400 equipped with PL-mixed D exclusion limit 400 k columns, and the polystyrene calibration curve was used for analyses. Differential scanning calorimetry was measured under a continuous nitrogen purge (20 mL/min) on a Perkin-Elmer Pyris 6 DSC instrument. The data were gathered on the secondary heating cycle using a heating and cooling scan rate of 10 °C/min.

## 4. Conclusions

This article reports detailed kinetics and a comprehensive mechanistic study for the catalytic alternating copolymerization of norbornene and ethylene, with use of the precursors (methylpalladium(II) cations (**1a**–**e**) bearing hemilabile bidentate of α-amino–pyridines).

The kinetic studies evidence that the key resting states are likely to be the ethyl(ethylene)palladium complexes (**5a’**–**e’**). The kinetic data of insertion reactions for **1a**–**e** reveal that the catalytic cycle is predominated by the N-insertion reactions, in which the N-coordination is rate-determining. The N-insertion in the *C*-isomer is significantly more facial (*k*_N_*^C^*^-**1e**^*/k*_N_*^T^*^-**1e**^ = 143 at 263 K), and the feasible geometrical isomerization is mainly controlled by the steric effects, cooperatively achieving the alternating N–E copolmerization.

The DFT calculations not only show consistent results with the kinetic measurements, but also afford a complimentary mechanistic understanding. The calculations for *C-***8’** also provide the favored E-insertion. The successive N-insertion in *C-***8’** will lead to racemic N-block.

This study thus is good example, of which the structure-reactivity relationship may be fine-tuned by steric control. The hemilabile unsymmetric bidentate ligand of α-amino–pyridine on the square planar organopalladium(II) cations demonstrate reactivities of convenient geometric isomerization and isomer-differentiated kinetic selectivity towards fundamental olefin-insertion. Such an unusual combination achieves the catalysis of COC-formation, with a high percentage of alternating microstructure.

## Figures and Tables

**Table 1:** Relative abundance for the isomers of **1** and **2**.

RHNCH_2_(*o*-C_6_H_4_N)	Me-Pd [8]			MeC_7_H_10_-Pd		
R		*T* (%)	*C* (%)		*T* (%) (*anti*/*syn*)	*C* (%)
*^i^*Pr	**1a**	82	18	**2a**	100 (30/70)	0
*^t^*Bu	**1b**	100	0	**2b**	100 (27/73)	0
Ph	**1c**	93	7	**2c**	96 (41/55)	4
2,6-Me_2_C_6_H_3_	**1d**	60	40	**2d**	87 (47/40)	13
2,6-*^i^*Pr_2_C_6_H_3_	**1e**	62	38	**2e**	94 (44/50)	6

**Table 2:** Kinetic data for norbornene insertion reactions of {[RHNCH_2_(*o*-C_6_H_4_N)]Pd(Me)(NCMe)}(BF_4_)(R = *^i^*Pr (**1a**), *^t^*Bu (**1b**), Ph (**1c**), 2,6-Me_2_C_6_H_3_ (**1d**), 2,6-*^i^*Pr_2_C_6_H_3_ (**1e**)) in CDCl_3_.

Entry	Cat	Temp. (K)	[Cat] (×10^−3^ M)	[N] ^a^/[Cat]	*k*_obs_ (10^−4^ s^−1^)	
					*C*	*T*
1	**1a**	263	5.3	28.7	- ^b^	8.69
2	**1b**	263	24.9	82.2	- ^c^	2.40
3	**1c**	263	2.0	23.2	- ^b^	51.60
4	**1d**	263	1.1	16.7	134.00	21.80
5	**1e**	263	3.4	10.8	- ^b^	5.47
6	**1e**	263	3.4	24.2	- ^b^	6.19
7	**1e**	263	3.4	62.6	- ^b^	10.10
8	**1e**	263	3.4	134.5	- ^b^	12.90
9	**1e**	263	3.4	260.9	- ^b^	23.80
10	**1e**	263	1.7	21.0	110.00	-
11	**1e**	258	3.3	31.1	109.00	2.35
12	**1e**	253	3.4	26.3	41.30	1.29
13	**1e**	243	3.3	28.0	16.80	-
14	**1e**	238	3.4	27.2	9.68	-
15	**1e**	233	3.3	20.1	7.11	-
16	**1e**	233	3.3	33.7	10.80	-
17	**1e**	233	3.3	40.5	12.90	-
18	**1e**	228	3.4	31.3	2.50	-

^a^ Determined by ^1^H-NMR, using tetramethylsilane as an internal standard; ^b^ the *C-*isomer was consumed under the conditions before the 1st measurement; ^c^ no *C*-isomer.

**Table 3:** The yields of E-coordinated Pd(II) species, from the reactions of **1** and ethylene in CDCl_3_.

RHNCH_2_(*o*-C_6_H_4_N)	E-Coord. Complexes					
R		*T* (%)	*C* (%)		*T* (%)	*C* (%)
*^i^*Pr	**1a’**	58	42	**5a’**	59	41
*^t^*Bu	**1b’**	63	37	**5b’**	58	42
Ph	**1c’**	100	0	**5c’**	100	0
2,6-Me_2_C_6_H_3_	**1d’**	59	41	**5d’**	64	36
2,6-*^i^*Pr_2_C_6_H_3_	**1e’**	38	62	**5e’**	67	33

**Table 4:** Kinetic data for the pseudo-first-order *k*_E_ of ethylene insertion reaction of **1a**–**e** in CDCl_3_.

Entry	Temp. (K)	Cat	[Cat] (10^−3^ M)	[E] ^a^/[Cat]	*k*_E_ (10^−4^ s^−1^)		
					*C*	*T*	Total ^b^
1	263	**1a**	5.3	18.3	^c^	^c^	6.44
2	263	**1b**	3.6	21.3	^d^	8.05	-
3	263	**1c**	4.4	25.9	^e^	1.93	-
4	263	**1d**	3.2	17.7	4.74	4.72	4.28
5	268	**1e**	3.4	21.7	13.10	14.30	13.60
6	263	**1e**	3.3	14.3	4.36	3.80	4.14
7	263	**1e**	3.3	25.0	4.46	4.19	4.37
8	263	**1e**	3.3	51.9	4.91	4.27	4.71
9	258	**1e**	3.4	49.4	3.40	3.20	3.37
10	253	**1e**	3.4	32.5	1.16	1.17	1.16
11	243	**1e**	3.3	27.2	0.41	0.41	0.41

^a^ Determined by ^1^H-NMR spectra using tetramethylsilane as an internal standard; ^b^ the rate is estimated from the sum of decrease in intensity of *C*- and *T*- isomers in ^1^H-NMR spectra; ^c^ the signals of Pd-Me for *C*- and *T*- isomers are overlapped; ^d^ all the signals are overlapped; and ^e^ lack of C- isomer.

**Table 5:** Norbornene blocks of E–N copolymers catalyzed by **1a**–**1e**. ^a^

Cat	Act. ^b^	NB_coc_^c^ (mol %)	Single ^c^ (mol %)	Diads ^c^ (mol %)	Triads ^c^ (mol %)	Alter. ^c^ (mol %)
1a	14	44.0	33.7	6.9	3.4	76.6
1b	5	42.9	39.0	3.9	0	81.9
1c	6	47.5	26.4	12.4	8.7	71.0
1d	27	48.1	34.8	6.9	6.4	80.8
1e	28	49.0	38.7	6.1	4.2	86.3

^a^ Reaction conditions: 0.06 mmol of catalysts, 21 bar of ethylene, 50 mL of CH_2_Cl_2_, 30 min, and room temperature; ^b^ Activity = kg (COC)mol^−1^(Pd) h^−1^. Determined by ^13^C-NMR.

## Supplementary Materials

Supplementary materials are available online. Syntheses and characterizations of complexes, crystallographic data, and CIF files of *T*-**2b** and *C*-**2e**, kinetic data of isomerization, ethylene, and norbornene insertion reactions, ^13^C-NMR spectra of COCs and theoretical calculation details.
