# Supplementary material for: Kinetics, Mechanism and Theoretical Studies of Norbornene-Ethylene Alternating Copolymerization Catalyzed by Organopalladium(II) Complexes Bearing Hemilabile α-Amino–pyridine
Source: Molecules. 2017 Jun 30;22(7):1095. doi: 10.3390/molecules22071095 (PMC6152412; doi:10.3390/molecules22071095)
Supplement: Supplementary file 1 [file molecules-22-01095-s001.pdf]

Kinetics, Mechanism, and Theoretical Studies of Norbornene–Ethylene Alternating Copolymerization Catalyzed by Organopalladium(II) Complexes Bearing Hemilabile α-Amino–pyridine

Kuo-Hsuan Yu [1], Shou-Ling Huang [1], Yi-Hung Liu [1], Yu Wang [1], Shiuh-Tzung Liu [1], Yuan-Chung Cheng [1,*], Ya-Fan Lin [1,2,*] and Jwu-Ting Chen [1,*]

[1] Department of Chemistry, National Taiwan University, Taipei 10617, Taiwan; [2] Department of Medicinal and Applied Chemistry, Kaohsiung Medical University, Kaohsiung 80708, Taiwan

Supplementary

Synthesis and Spectral Characterization **2**

^{1}H NMR spectra and assignments for *T*-**2e** and *C*-**2e** **8**

Crystallography data of *T*-**2b** and *C*-**2e** **9**

Copolymerization and Fineman-Ross data of **1e** **10**

Copolymer characterization and their ^{13}C NMR spectra **11**

Copolymerization data of **1b** and **2b** **15**

Time-resolved NMR spectra of *C*-**2e** **17**

Kinetic data for geometrical isomerism of *C*-**2e** **19**

Time-resolved NMR spectra for norbornene insertion reaction by taking **1a** as example **20**

Kinetic data for norbornene insertion reactions **21**

Time-resolved NMR spectra for ethylene insertion reaction by taking **1a** as example **25**

Knetic data for ethylene insertion reactions **26**

Example for the characterization of E-coordinated species by NOESY spectrum **28**

Van’t Hoff plot of **1e’** **29**

Theoretical calculation details **30**

References **33**

Synthesis and spectral characterization

{[^{i}PrHNCH$_2$(*o*-C$_6$H$_4$N)]Pd[(C$_7$H$_{10}$)Me](NCMe)}(BF$_4$) (2a). To a stirred solution of **1a** (260 mg, 0.65 mmol) in a 5:1 THF/acetonitrile mixture (12 mL) was added 1.3 equiv. of norbornene (84 mg, 0.89 mmol) in THF (6 mL) slowly. The mixture was stirred at room temperature for 1h, and then filtered through Celite. After removal of solvent *in vacuo*, the residue was washed with hexane (10 mL) and filtered. The crude product was washed with hexane (2 × 5 mL) again and dried in *vacuo*. The desired air-sensitive complex was obtained as light yellow powder in 83% yield (222 mg, 0.54 mmol). ^{1}H NMR (CDCl$_3$, 400 MHz) for major *T*-**2a**: δ 8.32 (d, J_{H-H} = 5.3 Hz, 1H, Py H-6), 7.87 (td, J_{H-H} = 7.5, 1.4 Hz, 1H, Py H-4), 7.42 (m, 1H, Py H-3; 1H, Py H-5), 4.53 (dd, J_{H-H} = 16.6, 6.4 Hz, 1H, Py-C*H*H'N), 3.94 (m, 1H, N*H*CH(CH$_3$)$_2$; 1H, Py-CH*H'*N), 2.75 (sept, J_{H-H} = 6.5 Hz, 1H, NHC*H*(CH$_3$)$_2$), 2.39 (bs, 3H, NCC*H$_3$*), 2.32 (d, J_{H-H} = 7.3 Hz, 1H, Pd-C*H*-), 2.13 (bs, 1H, bridgehead C*H*), 1.89 (d, J_{H-H} = 3.0 Hz, 1H, bridgehead C*H*), 1.73 (quin, J_{H-H} = 7.4 Hz, 1H, CH$_3$-C*H*-), 1.63 (m, 1H, *syn* apical C*H*H'), 1.53 (m, 1H, *exo*-C*H*H'-), 1.46 (d, J_{H-H} = 7.2 Hz, 3H, C*H$_3$*-CH-), 1.26 (m, 1H, *exo*-C*H*H'-), 1.19, 1.09 (d, J_{H-H} = 6.3, 6.5 Hz, 6H, NHCH(C*H$_3$*)$_2$), 1.18–1.00 (m, 1H, *anti* apical C*H*H'; 2H, *endo*-CH*H'*); minor *T*-**2a**: δ 8.42 (d, J_{H-H}= 5.2 Hz, 1H, Py H-6), 7.88 (m, 1H, Py H-4), 7.42 (m, 1H, Py H-3; 1H, Py H-5), 4.47 (dd, J_{H-H} = 16.6, 6.3 Hz, 1H, Py-C*H*H'N), 3.94 (m, 1H, N*H*CH(CH$_3$)$_2$; 1H, Py-CH*H'*N), 2.75 (m, 1H, NHC*H*(CH$_3$)$_2$), 2.39 (bs, 3H, NCC*H$_3$*), 2.31 (m, 1H, Pd-C*H*-), 2.16 (bs, 1H, bridgehead C*H*), 1.82 (bs, 1H, bridgehead C*H*), 1.63 (m, 1H, *syn* apical C*H*H'), 1.53 (m, 1H, *exo*-C*H*H'-), 1.46 (m, 3H, C*H$_3$*-CH-), 1.35 (m, 1H, CH$_3$-C*H*-), 1.26 (m, 1H, *exo*-C*H*H'-), 1.13, 1.05 (d, J_{H-H} = 7.1, 7.2 Hz, 6H, NHCH(C*H$_3$*)$_2$), 1.18–1.00 (m, 1H, *anti* apical C*H*H'; 2H, *endo*-CH*H'*). ^{13}C NMR (CDCl$_3$, 125.753 MHz) for major *T*-**2a**: δ 164.48 (Py C-2), 148.99 (Py C-6), 139.31 (Py C-4), 124.47 (Py C-5), 123.11 (Py C-3), 122.19 (N*C*CH$_3$), 53.77 (Py-*C*H$_2$N), 52.57 (Pd-*C*H-), 51.92 (NH*C*H(CH$_3$)$_2$), 45.22 (CH$_3$-*C*H-), 45.04, 44.78 (bridgehead *C*H), 34.60 (apical *C*H$_2$), 30.12, 29.32 (*C*H$_2$), 22.44, 21.34 (NHCH(*C*H$_3$)$_2$), 21.77 (*C*H$_3$-CH-), 3.29 (NC*C*H$_3$); minor *T*-**2a**: δ 164.25 (Py C-2), 146.90 (Py C-6), 139.51 (Py C-4), 123.98 (Py C-5), 123.48 (Py C-3), 122.06 (N*C*CH$_3$), 57.34 (Pd-*C*H-), 53.19 (Py-*C*H$_2$N), 51.39 (NH*C*H(CH$_3$)$_2$), 45.18 (CH$_3$-*C*H-), 45.61, 44.96 (bridgehead *C*H), 34.60 (apical *C*H$_2$), 30.28, 29.22 (*C*H$_2$), 22.75, 21.49 (NHCH(*C*H$_3$)$_2$), 21.12 (*C*H$_3$-CH-), 3.29 (NC*C*H$_3$). Anal. Calcd for $C_{19}H_{30}N_3PdBF_4$: C, 46.22; H, 6.12; N, 8.51. Found: C, 46.46; H, 6.33; N, 7.70. HR-MS (ESI, *m*/*z*): calcd for $C_{19}H_{30}N_3Pd$ [M-BF$_4$]$^+$ 406.1487, found: 406.1474.

{[^{t}BuHNCH$_2$(*o*-C$_6$H$_4$N)]Pd[(C$_7$H$_{10}$)Me](NCMe)}(BF$_4$) (2b). The synthesis was carried out according to the same procedure as for **2a**, using **1b** (234 mg, 0.57 mmol) and norbornene (69 mg, 0.74 mmol) to give the light yellow product **2b** (203 mg, 70%). Single crystals suitable for X-ray diffraction were grown by slow diffusion of Et$_2$O into a saturated MeCN/CH$_2$Cl$_2$ solution of **2b**. ^{1}H NMR (CDCl$_3$, 400 MHz) for major *T*-**2b**: δ 8.29 (d, J_{H-H} = 5.4 Hz, 1H, Py H-6), 7.86 (td, J_{H-H} = 7.7, 1.2 Hz, 1H, Py H-4), 7.47 (d, J_{H-H} = 7.8 Hz, 1H, Py H-3), 7.37 (t, J_{H-H} = 6.4 Hz, 1H, Py H-5), 4.42 (dd, J_{H-H} = 16.8, 7.1 Hz, 1H, Py-C*H*H'N), 4.05 (d, J_{H-H} = 17.2 Hz, 1H, Py-CH*H'*N), 3.91 (d, J_{H-H} = 7.0 Hz, 1H, N*H*C(CH$_3$)$_3$), 2.38 (bs, 3H, NCC*H$_3$*), 2.29 (d, J_{H-H} = 7.4 Hz, 1H, Pd-C*H*-), 2.11 (d, J_{H-H} = 2.9 Hz, 1H, bridgehead C*H*), 1.88 (d, J_{H-H} = 3.6 Hz, 1H, bridgehead C*H*), 1.76 (m, 1H, CH$_3$-C*H*-), 1.64 (d, J_{H-H} = 9.7 Hz, 1H, *syn* apical C*H*H'), 1.55 (m, 1H, *exo*-C*H*H'-), 1.50 (d, J_{H-H} = 7.2 Hz, 3H, C*H$_3$*-CH-), 1.30 (m, 1H, *exo*-C*H*H'-), 1.15 (s, 9H, NHC(C*H$_3$*)$_3$), 1.20–1.00 (m, 1H, *anti* apical C*H*H'; 2H, *endo*-CH*H'*); minor *T*-**2b**: δ 8.39 (d, J_{H-H} = 5.6 Hz, 1H, Py H-6), 7.88 (m, 1H, Py H-4), 7.46 (m, 1H, Py H-3), 7.37 (m, 1H, Py H-5), 4.42 (m, 1H, Py-C*H*H'N), 4.05 (m, 1H, Py-CH*H'*N), 3.91 (m, 1H, N*H*C(CH$_3$)$_3$), 2.38 (bs, 3H, NCC*H$_3$*), 2.31 (m, 1H, Pd-C*H*-), 2.16 (d, J_{H-H} = 3.2 Hz, 1H, bridgehead C*H*), 1.82 (m, 1H, bridgehead C*H*), 1.64 (m, 1H, *syn* apical C*H*H'), 1.55 (m, 1H, *exo*-C*H*H'-), 1.50 (m, 3H, C*H$_3$*-CH-), 1.30 (m, 1H, *exo*-C*H*H'-), 1.15 (s, 9H, NHC(C*H$_3$*)$_3$), 1.20–1.00 (m, 1H, CH$_3$-C*H*-; 1H, *anti* apical C*H*H'; 2H, *endo*-CH*H'*). ^{13}C NMR (CDCl$_3$, 100.625 MHz) for major *T*-**2b**: δ 166.22 (Py C-2), 148.76 (Py C-6), 139.16 (Py C-4), 124.19 (Py C-5), 122.41 (Py C-3), 122.41 (N*C*CH$_3$), 55.75 (NH*C*(CH$_3$)$_3$), 52.55 (Pd-*C*H-), 50.65 (Py-*C*H$_2$N), 45.36 (CH$_3$-*C*H-), 45.04, 44.89 (bridgehead *C*H), 34.65 (apical *C*H$_2$), 30.12, 29.42 (*C*H$_2$), 28.52 (NHC(*C*H$_3$)$_3$), 21.77 (*C*H$_3$-CH-), 3.42 (NC*C*H$_3$); minor *T*-**2b**: δ 166.14 (Py C-2), 146.43 (Py C-6), 139.39 (Py C-4), 123.83 (N*C*CH$_3$) 123.61 (Py C-5), 122.77 (Py C-3), 57.79 (Pd-*C*H-), 55.45 (NH*C*(CH$_3$)$_3$), 50.37 (Py-*C*H$_2$N), 45.73 (CH$_3$-*C*H-), 45.32, 44.97 (bridgehead *C*H), 34.65 (apical *C*H$_2$), 30.39, 29.27 (*C*H$_2$), 28.65 (NHC(*C*H$_3$)$_3$), 22.48 (*C*H$_3$-CH-), 3.42 (NC*C*H$_3$). Anal. Calcd for $C_{20}H_{32}N_3PdBF_4$: C, 47.31; H, 6.35; N, 8.28. Found: C, 46.55; H, 6.31; N, 7.83. HR-MS (ESI, *m*/*z*): calcd for $C_{20}H_{32}N_3Pd$ [M-BF$_4$]$^+$ 420.1630, found: 420.1631.

{[PhHNCH$_2$(*o*-C$_6$H$_4$N)]Pd[(C$_7$H$_{10}$)Me](NCMe)}(BF$_4$) (2c). The synthesis was carried out according to the same procedure as for **2a**, using **1c** (420 mg, 0.97 mmol) and norbornene (96 mg, 1.01 mmol) to give the light brown product **2c** (386 mg, 75%). ^{1}H NMR (CDCl$_3$, 400 MHz) for major *T*-**2c**: δ 8.32 (d, J_{H-H} = 5.5 Hz, 1H, Py H-6), 7.96 (m, 1H, Py H-4), 7.52 (d, J_{H-H} = 7.8 Hz, 1H, Py H-3), 7.45 (t, J_{H-H} = 6.6 Hz, 1H, Py H-5), 7.23–7.16 (m, 2H, *m*-Ar), 6.99 (t, J_{H-H} = 7.4 Hz, 1H, *p*-Ar), 6.77 (d, J_{H-H} = 7.8 Hz, 2H, *o*-Ar), 6.18 (d, J_{H-H} = 6.5 Hz, 1H, N*H*-Ar), 4.88 (dd, J_{H-H} = 16.9, 6.9 Hz, 1H, Py-C*H*H'N),

4.24 (d, J_{H-H} = 16.9 Hz, 1H, Py-CH*H'*N), 2.51 (d, J_{H-H} = 6.7 Hz, 1H, Pd-C*H*-), 2.31 (s, 3H, NCC*H*$_3$), 2.13 (d, J_{H-H} = 3.4 Hz, 1H, bridgehead C*H*), 1.81 (d, J_{H-H} = 3.0 Hz, 1H, bridgehead C*H*), 1.63 (d, J_{H-H} = 9.4 Hz, 1H, *syn* apical C*H*H'), 1.53–1.40 (m, 1H, CH_3-C*H*-; 1H, *exo*-C*H*H'-), 1.25 (m, 1H, *exo*-C*H*H'-), 1.12 (d, J_{H-H} = 7.2 Hz, 3H, C*H*$_3$-CH-), 1.10–1.00 (m, 1H, *anti* apical C*H*H'; 2H, *endo*-CH*H'*); minor *T*-**2c**: δ 8.38 (d, J_{H-H} = 5.3 Hz, 1H, Py H-6), 7.96 (m, 1H, Py H-4), 7.57 (d, J_{H-H} = 7.9 Hz, 1H, Py H-3), 7.45 (t, J_{H-H} = 6.6 Hz, 1H, Py H-5), 7.23–7.16 (m, 2H, *m*-Ar), 7.01 (t, J_{H-H} = 7.6 Hz, 1H, *p*-Ar), 6.87 (d, J_{H-H} = 7.9 Hz, 2H, *o*-Ar), 6.16 (bs, 1H, N*H*-Ar), 4.82 (dd, J_{H-H} = 16.6, 6.4 Hz, 1H, Py-C*H*H'N), 4.30 (dd, J_{H-H} = 16.5, 2.6 Hz, 1H, Py-CH*H'*N), 2.47 (d, J_{H-H} = 7.5 Hz, 1H, Pd-C*H*-), 2.23 (s, 3H, NCC*H*$_3$), 2.04 (d, J_{H-H} = 2.8 Hz, 1H, bridgehead C*H*), 1.83 (bs, 1H, bridgehead C*H*), 1.53–1.40 (m, 1H, *syn* apical C*H*H'; 1H, *exo*-C*H*H'-), 1.25 (m, 1H, *exo*-C*H*H'-), 1.21 (d, J_{H-H} = 7.1 Hz, 3H, C*H*$_3$-CH-), 1.12 (m, 1H, CH_3-C*H*-), 1.10–1.00 (m, 1H, *anti* apical C*H*H'; 2H, *endo*-CH*H'*). ^{13}C NMR ($CDCl_3$, 125.753 MHz) for major *T*-**2c**: δ 163.93 (Py C-2), 148.48 (Py C-6), 146.02 (*ipso*-Ar), 139.79 (Py C-4), 129.37 (*m*-Ar), 124.68 (Py C-5), 123.91 (*p*-Ar), 123.04 (Py C-3), 120.77 (N*C*CH_3), 118.63 (*o*-Ar), 56.67 (Pd-*C*H-), 55.38 (Py-*C*H_2N), 45.57, 44.90 (bridgehead *C*H), 44.77 (CH_3-*C*H-), 34.53 (apical *C*H_2), 29.64, 29.20 (*C*H_2), 22.19 (*C*H_3-CH-), 3.11 (NC*C*H_3); minor *T*-**2c**: δ 163.48 (Py C-2), 147.61 (Py C-6), 146.14 (*ipso*-Ar), 139.90 (Py C-4), 129.68 (*m*-Ar), 124.49 (Py C-5), 123.91 (*p*-Ar), 123.35 (Py C-3), 120.77 (N*C*CH_3), 118.76 (*o*-Ar), 58.37 (Pd-*C*H-), 55.19 (Py-*C*H_2N), 45.94 (bridgehead *C*H), 45.10 (CH_3-*C*H-), 44.90 (bridgehead *C*H), 34.53 (apical *C*H_2), 29.79, 29.20 (*C*H_2), 22.04 (*C*H_3-CH-), 2.92 (NC*C*H_3). Anal. Calcd for $C_{22}H_{28}N_3PdBF_4$: C, 50.07; H, 5.35; N, 7.96. Found: C, 50.27; H, 5.40; N, 6.71. HR-MS (ESI, *m/z*): calcd for $C_{22}H_{28}N_3Pd$ $[M-BF_4]^+$ 440.1339, found: 440.1318.

{[(2,6-Me$_2$C$_6$H$_3$)HNCH$_2$(*o*-C$_6$H$_4$N)]Pd[(C$_7$H$_{10}$)Me](NCMe)} (BF$_4$) (2d). The synthesis was carried out according to the same procedure as for **2a**, using **1d** (210 mg, 0.45 mmol) and norbornene (56 mg, 0.59 mmol) to give the light yellow product **2d** (182 mg, 73%). ^{1}H NMR ($CDCl_3$, 400 MHz) for major *T*-**2d**: δ 8.41 (d, J_{H-H} = 5.5 Hz, 1H, Py H-6), 7.90 (td, J_{H-H} = 7.7, 5.4 Hz, 1H, Py H-4), 7.51 (m, 1H, Py H-3), 7.43 (dd, J_{H-H} = 7.2, 6.6 Hz, 1H, Py H-5), 7.05 (m, 2H, *m*-Ar,), 6.96 (m, 1H, *p*-Ar), 5.44 (td, J_{H-H} = 6.4, 3.1 Hz, 1H, N*H*-Ar), 4.69 (dd, J_{H-H} = 35.8, 6.3 Hz, 1H, Py-C*H*H'N), 4.43 (m, 1H, Py-CH*H'*N), 3.00-2.40 (br, 6H, Ar-C*H*$_3$), 2.66 (s, 1H, Pd-C*H*-), 2.05 (bs, 1H, bridgehead C*H*), 1.85 (d, J_{H-H} = 3.2 Hz, 1H, bridgehead C*H*), 1.68 (bs, 3H, NCC*H*$_3$; 1H, CH_3-C*H*-), 1.52–1.42 (m, 1H, *syn* apical C*H*H'; 2H, *exo*-C*H*H'-), 1.25 (d, J_{H-H} = 7.0 Hz, 3H, C*H*$_3$-CH-), 1.30–1.10 (m, 2H, *endo*-CH*H'*), 1.10–1.00 (m, 1H, *anti* apical C*H*H'); minor *T*-**2d**: δ 8.36 (d, J_{H-H} = 5.3 Hz, 1H, Py H-6), 7.90 (td, J_{H-H} = 7.7, 5.4 Hz, 1H, Py H-4), 7.51 (m, 1H, Py H-3), 7.43 (dd, J_{H-H} = 7.2, 6.6 Hz, 1H, Py H-5), 7.05 (m, 2H, *m*-Ar,), 6.96 (m, 1H, *p*-Ar), 5.35 (td, J_{H-H} = 5.8, 4.4 Hz, 1H, N*H*-Ar), 4.73 (dd, J_{H-H} = 35.9, 6.2 Hz, 1H, Py-C*H*H'N), 4.43 (m, 1H, Py-CH*H'*N), 3.00-2.40 (br, 6H, Ar-C*H*$_3$), 2.64 (s, 1H, Pd-C*H*-), 2.14 (d, J_{H-H} = 2.4 Hz, 1H, bridgehead C*H*), 1.87 (s, 1H, bridgehead C*H*), 1.66 (bs, 3H, NCC*H*$_3$), 1.57 (m, 1H, CH_3-C*H*-), 1.52–1.42 (m, 1H, *syn* apical C*H*H'; 1H, *exo*-C*H*H'-), 1.25 (d, J_{H-H} = 7.0 Hz, 3H, C*H*$_3$-CH-; 1H, *exo*-C*H*H'-), 1.30–1.10 (m, 2H, *endo*-CH*H'*), 1.10–1.00 (m, 1H, *anti* apical C*H*H'). ^{13}C NMR ($CDCl_3$, 100.625 MHz) for major *T*-**2d**: δ 162.59 (Py C-2), 147.10 (Py C-6), 141.66 (*ipso*-Ar), 139.44 (Py C-4), 130.03 (*o*-Ar), 129.86 (*m*-Ar),125.22 (*p*-Ar), 124.34 (Py C-5), 123.70 (Py C-3), 119.49 (N*C*CH_3), 59.79 (Pd-*C*H-), 54.32 (Py-*C*H_2N), 46.02 (bridgehead *C*H), 45.44 (CH_3-*C*H-), 45.26 (bridgehead *C*H), 34.68 (apical *C*H_2), 29.75, 29.29 (*C*H_2), 22.33 (*C*H_3-CH-), 19.29, 18.93 (Ar-*C*H_3), 1.78 (NC*C*H_3); minor *T*-**2d**: δ 163.02 (Py C-2), 148.26 (Py C-6), 141.44 (*ipso*-Ar), 139.41 (Py C-4), 130.03 (*o*-Ar), 129.86 (*m*-Ar), 125.28 (*p*-Ar), 124.61 (Py C-5), 123.74 (Py C-3), 119.74 (N*C*CH_3), 59.53 (Pd-*C*H-), 54.36 (Py-*C*H_2N), 46.45, 45.26 (bridgehead *C*H), 44.99 (CH_3-*C*H-), 34.68 (apical *C*H_2), 29.90, 29.19 (*C*H_2), 21.80 (*C*H_3-CH-), 19.29, 18.93 (Ar-*C*H_3), 1.78 (NC*C*H_3). Anal. Calcd for $C_{24}H_{32}N_3PdBF_4$: C, 51.87; H, 5.80; N, 7.56. Found: C, 52.18; H, 6.02; N, 6.55. HR-MS (ESI, *m/z*): calcd for $C_{24}H_{32}N_3Pd$ $[M-BF_4]^+$ 468.1629, found: 468.1631.

{[(2,6-^{i}Pr$_2$C$_6$H$_3$)HNCH$_2$(*o*-C$_6$H$_4$N)]Pd[(C$_7$H$_{10}$)Me](NCMe)} (BF$_4$) (2e). The synthesis was carried out according to the same procedure as for **2a**, using **1e** (365 mg, 0.71 mmol) and norbornene (86 mg, 0.92 mmol) to give the light yellow product **2e** (340 mg, 78%). Single crystals suitable for X-ray diffraction were grown by slow diffusion of Et_2O into a saturated $MeCN/CH_2Cl_2$ solution of **2e**. ^{1}H NMR ($CDCl_3$, 400 MHz, 298 K) for major *T*-**2e**: δ 8.37 (d, J_{H-H} = 5.1 Hz, 1H, Py H-6), 7.95 (t, J_{H-H} = 7.6 Hz, 1H, Py H-4), 7.54 (bs, 1H, Py H-3), 7.46 (t, J_{H-H} = 6.6 Hz, 1H, Py H-5), 7.23–7.05 (m, 2H, *m*-Ar; 1H, *p*-Ar), 5.46 (td, J_{H-H} = 6.5, 4.4 Hz, 1H, N*H*-Ar), 4.59 (dd, J_{H-H} = 16.3, 4.6 Hz, 1H, Py-C*H*H'N), 4.32 (m, 1H, Py-CH*H'*N, 1H, Ar-C*H*(CH_3)$_2$), 3.29 (bs, 1H, Ar-C*H*(CH_3)$_2$), 2.68 (d, J_{H-H} = 7.4 Hz, 1H, Pd-C*H*-), 2.03 (bs, 1H, bridgehead C*H*), 1.88 (bs, 1H, bridgehead C*H*), 1.67 (bs, 1H, CH_3-C*H*-), 1.60 (bs, 3H, NCC*H*$_3$), 1.59–1.45 (m, 1H, *syn* apical C*H*H'; 2H, *exo*-C*H*H'-), 1.45–1.30 (m, 3H, C*H*$_3$-CH-; 1H, *endo*-CH*H'*), 1.35–1.15 (m, 12H, Ar-CH(C*H*$_3$)$_2$), 1.20–1.11 (m, 1H, *endo*-CH*H'*), 1.10–0.95 (m, 1H, *anti* apical C*H*H'); minor *T*-**2e**: δ 8.42 (d, J_{H-H} = 5.3 Hz, 1H, Py H-6), 7.95 (t, J_{H-H} = 7.6 Hz, 1H, Py H-4), 7.54 (bs, 1H, Py H-3), 7.46 (t, J_{H-H} = 6.6 Hz, 1H, Py H-5), 7.23–7.05 (m, 2H, *m*-Ar; 1H, *p*-Ar), 5.54 (td, J_{H-H} = 5.8, 3.7 Hz, 1H, N*H*-Ar), 4.59 (dd, J_{H-H} = 16.3, 4.6 Hz, 1H, Py-C*H*H'N), 4.65 (bs, 1H, Ar-C*H*(CH_3)$_2$), 4.32 (m, 1H, Py-CH*H'*N), 3.29 (bs, 1H, Ar-C*H*(CH_3)$_2$), 2.72 (d, J_{H-H} = 7.2 Hz, 1H, Pd-C*H*-), 2.15 (bs, 1H, bridgehead C*H*), 1.88 (bs, 1H, bridgehead C*H*), 1.67 (bs, 1H,

CH_3-C*H*-), 1.60 (bs, 3H, NCCH_3), 1.59–1.45 (m, 1H, *syn* apical C*H*H'; 2H, *exo*-C*H*H'-), 1.45–1.30 (m, 3H, CH_3-CH-; 1H, *endo*-CH*H'*), 1.35–1.15 (m, 12H, Ar-CH(CH_3)$_2$), 1.20–0.95 (m, 1H, *endo*-CH*H*), 1.10–0.95 (m, 1H, *anti* apical C*H*H' '). ^{1}H NMR ($CDCl_3$, 400 MHz, 248 K) for *C*-**2e**: δ 8.63 (d, J_{H-H} = 5.0 Hz, 1H, Py H-6), 7.89 (t, J_{H-H} = 7.5 Hz, 1H, Py H-4), 7.76 (dd, J_{H-H} = 6.5, 6.1 Hz, 1H, Py H-5), 7.38–7.30 (m, 2H, *m*-Ar; 1H, *p*-Ar), 7.19 (d, J_{H-H} = 5.5 Hz, 1H, Py H-3), 6.06 (t, J_{H-H} = 7.5 Hz, 1H, N*H*-Ar), 4.62 (dd, J_{H-H} = 16.6, 5.9 Hz, 1H, Py-C*H*H'N), 4.50 (sept, J_{H-H} = 6.5 Hz, 1H, Ar-C*H*(CH_3)$_2$), 4.43 (dd, J_{H-H} = 16.9, 10.1 Hz, 1H, Py-CH*H'*N), 3.03 (sept, J_{H-H} = 6.4 Hz, 1H, Ar-C*H*(CH_3)$_2$), 2.57 (s, 3H, NCCH_3), 1.97 (s, 1H, bridgehead C*H*), 1.73 (s, 1H, bridgehead C*H*), 1.49 (d, 1H, J_{H-H} = 10.2 Hz, *syn* apical C*H*H'), 1.46 (m, 3H, CH_3-CH-; 3H, Ar-CH(CH_3)$_2$), 1.39 (d, J_{H-H} = 6.2 Hz, 3H, Ar-CH(CH_3)$_2$), 1.33 (d, J_{H-H} = 6.9 Hz, 3H, Ar-CH(CH_3)$_2$), 1.32 (d, J_{H-H} = 6.9 Hz, 3H, Ar-CH(CH_3)$_2$), 1.24 (t, J_{H-H} = 7.2 Hz, 1H, *exo*-C*H*H'-), 1.16 (d, J_{H-H} = 7.3 Hz, 1H, Pd-C*H*-), 1.00 (m, 1H, CH_3-C*H*-; 1H, *anti* apical C*H*H'; 1H, *exo*-C*H*H'-), 0.66 (bs, 1H, *endo*-CH*H'*), 0.42 (bs, 1H, *endo*-CH*H'*). ^{13}C NMR ($CDCl_3$, 100.625 MHz, 298 K) for major *T*-**2e**: δ 162.36 (Py C-2), 148.28 (Py C-6), 139.63 (*ipso*-Ar), 138.80 (Py C-4), 126.27 (*m*-Ar), 125.66 (*o*-Ar), 124.75 (Py C-5), 123.82 (Py C-3, *p*-Ar), 119.92 (N*C*CH_3), 58.21 (Pd-*C*H-), 57.05 (Py-*C*H_2N), 46.09 (bridgehead *C*H), 45.38 (CH_3-*C*H-), 44.88 (bridgehead *C*H), 34.68 (apical *C*H_2), 29.95, 29.18 (*C*H_2), 28.27, 27.75 (Ar-*C*H(CH_3)$_2$), 25.54, 25.24, 24.52, 23.28 (Ar-CH(*C*H_3)$_2$), 22.93 (*C*H_3-CH-), 1.73 (NC*C*H_3); minor *T*-**2e**: δ 162.31 (Py C-2), 147.26 (Py C-6), 139.63 (*ipso*-Ar), 139.58 (Py C-4), 126.27 (*m*-Ar), 125.90 (*o*-Ar), 124.52 (Py C-5), 123.82 (Py C-3, *p*-Ar), 119.66 (N*C*CH_3), 60.05 (Pd-*C*H-), 57.05 (Py-*C*H_2N), 46.59 (bridgehead *C*H), 45.38 (CH_3-*C*H-), 44.78 (bridgehead *C*H), 34.68 (apical *C*H_2), 29.56, 29.30 (*C*H_2), 28.33, 27.75 (Ar-*C*H(CH_3)$_2$), 25.54, 25.24, 24.52, 23.28 (Ar-CH(*C*H_3)$_2$), 21.84 (*C*H_3-CH-), 0.98 (NC*C*H_3). Anal. Calcd for $C_{28}H_{40}N_3PdBF_4$: C, 54.96; H, 6.59; N, 6.87. Found: C, 54.99; H, 6.89; N, 6.19. HR-MS (ESI, *m/z*): calcd for $C_{28}H_{40}N_3Pd$ [M-BF_4]$^+$ 524.2281, found: 524.2257.

{[^{i}PrHNCH$_2$(*o*-C$_6$H$_4$N)]Pd(Me)(CH$_2$=CH$_2$)}(BF$_4$) (1a'). ^{1}H NMR ($CDCl_3$, 500 MHz, 263 K) for *T*-**1a'**: δ 8.33 (bs, 1H, Py H-6), 7.99 (bs, 1H, Py H-4), 7.63–7.40 (m, 1H, Py H-3; 1H, Py H-5), 4.85 (bs, 1H, N*H*CH(CH_3)$_2$), 4.56 (dd, J_{H-H} = 16.3, 6.1 Hz, 1H, Py-C*H*H'N), 4.06 (d, J_{H-H} = 16.5 Hz, 1H, Py-CH*H'*N), 2.84 (sept, J_{H-H} = 6.3 Hz, 1H, NHC*H*(CH_3)$_2$), 1.16 (d, J_{H-H} = 5.7 Hz, 3H, NHCH(CH_3)$_2$), 1.11 (d, J_{H-H} = 6.2 Hz, 3H, NHCH(CH_3)$_2$), 0.64 (bs, 3H, Pd-CH_3); *C*-**1a'**: δ 7.89 (m, 1H, Py H-6; 1H, Py H-4), 7.63–7.40 (m, 1H, Py H-3; 1H, Py H-5), 4.26 (d, J_{H-H} = 17.1 Hz, 1H, Py-C*H*H'N), 4.08 (bs, 1H, Py-CH*H'*N), 3.98 (bs, 1H, N*H*CH(CH_3)$_2$), 3.22 (bs, 1H, NHC*H*(CH_3)$_2$), 1.22 (d, J_{H-H} = 6.1 Hz, 3H, NHCH(CH_3)$_2$), 1.16 (d, J_{H-H} = 5.7 Hz, 3H, NHCH(CH_3)$_2$), 0.68 (bs, 3H, Pd-CH_3). The Pd(CH_2=CH_2) signals are overlapped with free ethylene.

{[^{t}BuHNCH$_2$(*o*-C$_6$H$_4$N)]Pd(Me)(CH$_2$=CH$_2$)}(BF$_4$) (1b'). ^{1}H NMR ($CDCl_3$, 500 MHz, 263 K) for *T*-**1b'**: δ 8.29 (bs, 1H, Py H-6), 7.98 (bs, 1H, Py H-4), 7.54 (bs, 1H, Py H-3), 7.48 (bs, 1H, Py H-5), 4.87 (bs, 1H, N*H*C(CH_3)$_3$), 4.58 (dd, J_{H-H} = 18.1, 5.5 Hz, 1H, Py-C*H*H'N), 4.17 (d, J_{H-H} = 18.2 Hz, 1H, Py-CH*H'*N), 1.18, 1.07 (s, 9H, NHC(CH_3)$_3$), 0.68 (s, 1H, Pd-CH_3); *C*-**1b'**: δ 7.89 (bs, 1H, Py H-6; 1H, Py H-4), 7.54 (bs, 1H, Py H-3), 7.44 (bs, 1H, Py H-5), 4.41 (bs, 1H, Py-C*H*H'N), 3.92 (d, J_{H-H} = 5.4 Hz, 1H, Py-CH*H'*N), 1.20, 1.10 (s, 9H, NHC(CH_3)$_3$), 0.76 (s, 1H, Pd-CH_3). The N*H*C(CH_3)$_3$ signal of *C*-**1b'** is obscured. The Pd(CH_2=CH_2) signals are overlapped with free ethylene.

{[PhHNCH$_2$(*o*-C$_6$H$_4$N)]Pd(Me)(CH$_2$=CH$_2$)}(BF$_4$) (1c'). ^{1}H NMR ($CDCl_3$, 500 MHz, 263 K) for *T*-**1c'**: δ 8.37 (bs, 1H, Py H-6), 8.07 (t, J_{H-H} = 8.0 Hz, 1H, Py H-4), 7.61 (d, J_{H-H} = 8.0 Hz, 1H, Py H-3), 7.57 (t, J_{H-H} = 5.7 Hz, 1H, Py H-5), 7.27 (t, J_{H-H} = 7.5 Hz, 1H, *m*-Ar), 7.11 (m, 1H, *m*-Ar), 6.90 (d, J_{H-H} = 7.0 Hz , 1H, *o*-Ar), 6.85 (bs, 1H, *p*-Ar), 6.48 (d, J_{H-H} = 7.4 Hz, 1H, *o*-Ar), 6.28 (bs, 1H, N*H*-Ar), 5.01 (d, J_{H-H} = 16.7 Hz, 1H, Py-C*H*H'N), 4.43 (d, J_{H-H} = 17.4 Hz, 1H, Py-CH*H'*N), 0.72 (s, 3H, Pd-CH_3). The Pd(CH_2=CH_2) signals are overlapped with free ethylene.

{[(2,6-Me$_2$C$_6$H$_3$)HNCH$_2$(*o*-C$_6$H$_4$N)]Pd(Me)(CH$_2$=CH$_2$)} (BF$_4$) (1d'). ^{1}H NMR ($CDCl_3$, 500 MHz, 263 K) for *T*-**1d'**: δ 8.40 (d, J_{H-H} = 4.1 Hz, 1H, Py H-6), 7.99 (t, J_{H-H} = 7.1 Hz, 1H, Py H-4), 7.50 (bs, 1H, Py H-3), 7.45 (bs, 1H, Py H-5), 7.15–6.98 (m , 2H, *m*-Ar; 1H, *p*-Ar), 5.88 (bs, 1H, N*H*-Ar), 4.93 (dd, J_{H-H} = 17.1, 7.3 Hz, 1H, Py-C*H*H'N), 4.53 (bs, 2H, Pd(CH_2=CH$_2$)), 4.29 (dd, J_{H-H} = 16.8, 8.1 Hz, 1H, Py-CH*H'*N), 4.13 (bs, 2H, Pd(CH$_2$=CH_2)), 2.71 (s, 3H, Ar-CH_3), 2.48 (s, 3H, Ar-CH_3), 0.81 (s, 3H, Pd-CH_3); *C*-**1d'**: δ 7.90 (bs, 1H, Py H-6; 1H, Py H-4), 7.51 (bs, 1H, Py H-3), 7.45 (bs, 1H, Py H-5), 7.15-6.98 (m , 2H, *m*-Ar; 1H, *p*-Ar), 6.94 (bs, 1H, N*H*-Ar), 5.00 (dd, J_{H-H} = 17.5, 6.3 Hz, 1H, Py-C*H*H'N), 4.42 (dd, J_{H-H} = 17.4, 7.7 Hz, 1H, Py-CH*H'*N), 2.71 (s, 3H, Ar-CH_3), 2.51 (s, 3H, Ar-CH_3), 0.06 (s, 3H, Pd-CH_3). The Pd(CH_2=CH_2) signals of *C*-**1d'** are overlapped with free ethylene.

{[(2,6-^{i}Pr$_2$C$_6$H$_3$)HNCH$_2$(*o*-C$_6$H$_4$N)]Pd(Me)(CH$_2$=CH$_2$)}(BF$_4$) (1e'). ^{1}H NMR ($CDCl_3$, 500 MHz, 263 K) for *T*-**1e'**: δ 8.40 (d, J_{H-H} = 3.5 Hz, 1H, Py H-6), 7.98 (t, J_{H-H} = 7.1 Hz, 1H, Py H-4), 7.57–7.38 (m, 1H, Py H-3; 1H, Py H-5), 7.20 (bs, 2H, *m*-Ar; 1H, *p*-Ar), 5.89 (bs, 1H, N*H*-Ar), 5.10 (dd, J_{H-H} = 17.9, 7.7 Hz, 1H, Py-C*H*H'N), 4.62 (bs, 2H, Pd(CH_2=CH$_2$)), 4.19 (dd, J_{H-H} = 18.2, 7.2 Hz, 1H, Py-CH*H'*N), 4.15 (bs, 2H, Pd(CH$_2$=CH_2)), 3.68 (bs, 1H, Ar-C*H*(CH_3)$_2$), 3.32 (bs, 1H, Ar-C*H*(CH_3)$_2$), 1.47–1.09 (m, 12H, Ar-CH(CH_3)$_2$), 0.80 (s, 1H, Pd-CH_3); *C*-**1e'**: δ 7.90 (bs, 1H, Py H-6; 1H, Py H-4), 7.57–7.38 (m, 1H, Py H-3; 1H, Py H-5), 7.20 (bs, 2H, *m*-Ar; 1H, *p*-Ar), 6.97 (bs, 1H, N*H*-Ar), 5.02 (dd, J_{H-H} = 17.4, 5.8 Hz, 1H, Py-

C*H*H'N), 4.38 (dd, J_{H-H} = 16.7, 8.1 Hz, 1H, Py-CH*H'*N), 3.92, 3.32 (bs, 2H, Ar-C*H*(CH_3)$_2$), 1.47–1.09 (m, 12H, Ar-CH(CH_3)$_2$), 0.15 (s, 1H, Pd-CH_3). The Pd(CH_2=CH_2) signals of *C*-**1e'** are overlapped with free ethylene.

{[^{i}PrHNCH$_2$(*o*-C$_6$H$_4$N)]Pd(Et)(NCMe)}(BF$_4$) (5a). ^{1}H NMR ($CDCl_3$, 500 MHz, 263 K) for *T*-**5a**: δ 8.28 (d, J_{H-H} = 5.2 Hz, 1H, Py H-6), 7.88 (t, J_{H-H} = 7.4 Hz, 1H, Py H-4), 7.44 (d, J_{H-H} = 8.2 Hz, 1H, Py H-3), 7.40 (d, J_{H-H} = 6.2 Hz, 1H, Py H-5), 4.50 (dd, J_{H-H} = 17.4, 5.6 Hz, 1H, Py-C*H*H'N), 4.11 (bs, 1H, N*H*CH(CH_3)$_2$), 3.89 (d, J_{H-H} = 16.5 Hz, 1H, Py-CH*H'*N), 2.77 (sept, J_{H-H} = 6.0 Hz, 1H, NHC*H*(CH_3)$_2$), 2.41 (s, 3H, NCCH_3), 1.74 (q, J_{H-H} = 7.7 Hz, 2H, Pd-CH_2CH$_3$), 1.25–1.07 (m, 6H, NHCH(CH_3)$_2$), 0.78 (t, J_{H-H} = 7.1 Hz, 3H, Pd-CH$_2$CH_3).

{[^{t}BuHNCH$_2$(*o*-C$_6$H$_4$N)]Pd(Et)(NCMe)}(BF$_4$) (5b). ^{1}H NMR ($CDCl_3$, 500 MHz, 263 K) for *T*-**5b**: δ 8.27 (d, J_{H-H} = 5.5 Hz, 1H, Py H-6), 7.86 (t, J_{H-H} = 7.3 Hz, 1H, Py H-4), 7.41 (d, J_{H-H} = 7.9 Hz, 1H, Py H-3), 7.37 (t, J_{H-H} = 6.7 Hz,, 1H, Py H-5), 4.42 (dd, J_{H-H} = 17.1, 7.3 Hz, 1H, Py-C*H*H'N), 4.13 (d, J_{H-H} = 6.8 Hz, 1H, N*H*C(CH_3)$_3$), 4.00 (d, J_{H-H} = 17.0 Hz, 1H, Py-CH*H'*N), 2.40 (s, 3H, NCCH_3), 1.74 (q, J_{H-H} = 7.8 Hz, 2H, Pd-CH_2CH$_3$), 1.10 (s, 9H, NHC(CH_3)$_3$), 0.77 (t, J_{H-H} = 7.5 Hz, 3H, Pd-CH$_2$CH_3).

{[PhHNCH$_2$(*o*-C$_6$H$_4$N)]Pd(Et)(NCCH$_3$)}(BF$_4$) (5c). ^{1}H NMR ($CDCl_3$, 500 MHz, 263 K) for *T*-**5c**: δ 8.36 (bs, 1H, Py H-6), 7.85 (t, J_{H-H} = 7.5 Hz, 1H, Py H-4), 7.53 (d, J_{H-H} = 7.9 Hz, 1H, Py H-3), 7.32 (d, J_{H-H} = 6.3 Hz, 1H, Py H-5), 7.26 (t, J_{H-H} = 7.7 Hz, 1H, *m*-Ar), 7.10 (m, 1H, *m*-Ar; 1H, *o*-Ar; 1H, *p*-Ar), 6.89 (d, J_{H-H} = 6.9 Hz, 1H, *o*-Ar), 6.48 (bs, 1H, N*H*-Ar), 4.95 (bs, 1H, Py-C*H*H'N), 4.41 (bs, 1H, Py-CH*H'*N), 2.01 (s, 3H, NCCH_3), 1.67 (bs, 2H, Pd-CH_2CH$_3$), 0.67 (t, J_{H-H} = 7.6 Hz, 3H, Pd-CH$_2$CH_3).

{[^{i}PrHNCH$_2$(*o*-C$_6$H$_4$N)]Pd(Et)(CH$_2$=CH$_2$)}(BF$_4$) (5a'). ^{1}H NMR ($CDCl_3$, 500 MHz, 263 K) for *T*-**5a'**: δ 8.36 (d, J_{H-H} = 5.7 Hz, 1H, Py H-6), 7.97 (bs, 1H, Py H-4), 7.55 (d, J_{H-H} = 7.9 Hz, 1H, Py H-3), 7.52 (bs, 1H, Py H-5), 4.55 (bs, 1H, Py-C*H*H'N), 4.11 (bs, 1H, Py-CH*H'*N), 3.61 (bs, 1H, N*H*CH(CH_3)$_2$), 284 (bs, 1H, NHC*H*(CH_3)$_2$), 1.40 (q, J_{H-H} = 7.3 Hz, 2H, Pd-CH_2CH$_3$), 1.25–1.07 (m, 6H, NHCH(CH_3)$_2$), 0.92 (t, J_{H-H} = 7.2 Hz, 3H, Pd-CH$_2$CH_3); *C*-**5a'**: δ 7.97 (bs, 1H, Py H-4), 7.82 (bs, 1H, Py H-6), 7.60–7.34 (m, 1H, Py H-3; 1H, Py H-5), 4.55 (bs, 1H, Py-C*H*H'N), 4.11 (bs, 1H, Py-CH*H'*N), 3.61 (bs, 1H, N*H*CH(CH_3)$_2$), 2.84 (bs, 1H, NHC*H*(CH_3)$_2$), 1.38 (q, J_{H-H} = 7.8 Hz, 2H, Pd-CH_2CH$_3$), 1.25–1.07 (m, 6H, NHCH(CH_3)$_2$), 0.92 (t, J_{H-H} = 7.2 Hz, 3H, Pd-CH$_2$CH_3). The Pd(CH_2=CH_2) signals are overlapped with free ethylene.

{[^{t}BuHNCH$_2$(*o*-C$_6$H$_4$N)]Pd(Et)(CH$_2$=CH$_2$)}(BF$_4$) (5b'). ^{1}H NMR ($CDCl_3$, 500 MHz, 263 K) for *T*-**5b'**: δ 8.30 (bs, 1H, Py H-6), 7.97 (t, J_{H-H} = 7.2 Hz, 1H, Py H-4), 7.54 (d, J_{H-H} = 7.8 Hz, 1H, Py H-3), 7.51 (bs, 1H, Py H-5), 4.51 (bs, 1H, Py-C*H*H'N), 4.18 (bs, 1H, Py-CH*H'*N), 3.61 (bs, 1H, N*H*C(CH_3)$_3$), 1.40 (q, J_{H-H} = 7.6 Hz, 2H, Pd-CH_2CH$_3$), 1.10 (s, 9H, NHC(CH_3)$_3$), 0.92 (t, J_{H-H} = 7.5 Hz, 3H, Pd-CH$_2$CH_3); *C*-**5b'**: δ 8.05 (bs, 1H, Py H-6; 1H, Py H-4), 7.54 (d, J_{H-H} = 7.8 Hz, 1H, Py H-3), 7.51 (bs, 1H, Py H-5), 4.51 (bs, 1H, Py-C*H*H'N), 4.18 (bs, 1H, Py-CH*H'*N), 3.61 (bs, 1H, N*H*C(CH_3)$_3$), 1.38 (q, J_{H-H} = 7.8 Hz, 2H, Pd-CH_2CH$_3$), 1.10 (s, 9H, NHC(CH_3)$_3$), 0.92 (t, J_{H-H} = 7.5 Hz, 3H, Pd-CH$_2$CH_3). The Pd(CH_2=CH_2) signals are overlapped with free ethylene.

{[PhHNCH$_2$(*o*-C$_6$H$_4$N)]Pd(Et)(CH$_2$=CH$_2$)}(BF$_4$) (5c'). ^{1}H NMR ($CDCl_3$, 500 MHz, 263 K) for *T*-**5c'**: δ 8.42 (bs, 1H, Py H-6), 8.06 (t, J_{H-H} = 7.6 Hz, 1H, Py H-4), 7.60 (m, 1H, Py H-3; 1H, Py H-5), 7.26 (t, J_{H-H} = 7.7 Hz, 1H, *m*-Ar), 7.10 (t, J_{H-H} = 6.6 Hz, 1H, *m*-Ar), 6.89 (d, J_{H-H} = 6.8 Hz , 1H, *o*-Ar), 6.83 (t, J_{H-H} = 7.3 Hz , 1H, *p*-Ar), 6.45 (d, J_{H-H} = 7.9 Hz, 1H, *o*-Ar), 6.14 (bs, 1H, N*H*-Ar), 4.95 (bs, 1H, Py-C*H*H'N), 4.41 (bs, 1H, Py-CH*H'*N), 1.51 (br q, J_{H-H} = 6.3 Hz, 2H, Pd-CH_2CH$_3$), 0.88 (bs, 3H, Pd-CH$_2$CH_3). The Pd(CH_2=CH_2) signals are overlapped with free ethylene.

{[(2,6-Me$_2$C$_6$H$_3$)HNCH$_2$(*o*-C$_6$H$_4$N)]Pd(Et)(CH$_2$=CH$_2$)} (BF$_4$) (5d'). ^{1}H NMR ($CDCl_3$, 500 MHz, 263 K) for *T*-**5d'**: δ 8.37 (bs, 1H, Py H-6), 7.99 (bs, 1H, Py H-4), 7.63–7.38 (m, 1H, Py H-3; 1H, Py H-5), 7.16–6.97 (m , 2H, *m*-Ar; 1H, *p*-Ar), 5.69 (bs, 1H, N*H*-Ar), 4.75 (bs, 1H, Py-C*H*H'N), 4.44 (bs, 2H, Pd(CH_2=CH$_2$)), 4.26 (bs, 1H, Py-CH*H'*N), 4.07 (bs, 2H, Pd(CH$_2$=CH_2)), 2.72 (s, 3H, Ar-CH_3), 2.45 (s, 3H, Ar-CH_3), 1.33 (bs, 2H, Pd-CH_2CH$_3$), 0.84 (m, 3H, Pd-CH$_2$CH_3); *C*-**5d'**: δ 7.89 (bs, 1H, Py H-6; 1H, Py H-4), 7.63–7.38 (m, 1H, Py H-3; 1H, Py H-5), 7.16–6.97 (m , 2H, *m*-Ar; 1H, *p*-Ar), 6.72 (bs, 1H, N*H*-Ar), 5.09 (bs, 1H, Py-C*H*H'N), 4.35 (bs, 1H, Py-CH*H'*N), 2.72 (s, 3H, Ar-CH_3), 2.54 (s, 3H, Ar-CH_3), 1.33 (bs, 2H, Pd-CH_2CH$_3$), 0.84 (m, 3H, Pd-CH$_2$CH_3). The Pd(CH_2=CH_2) signals of *C*-**5d'** are overlapped with free ethylene.

{[(2,6-^{i}Pr$_2$C$_6$H$_3$)HNCH$_2$(*o*-C$_6$H$_4$N)]Pd(Et)(CH$_2$=CH$_2$)} (BF$_4$) (5e'). ^{1}H NMR ($CDCl_3$, 500 MHz, 263 K) for *T*-**5e'**: δ 8.36 (bs, 1H, Py H-6), 7.98 (t, J_{H-H} = 7.6 Hz, 1H, Py H-4), 7.55 (bs, 1H, Py H-3), 7.45 (m, 1H, Py H-5), 7.19 (bs, 2H, *m*-Ar; 1H, *p*-Ar), 5.68 (bs, 1H, N*H*-Ar), 5.02 (dd, J_{H-H} = 17.6, 8.4 Hz, 1H, Py-C*H*H'N), 4.54 (bs, 2H, Pd(CH_2=CH$_2$)), 4.16 (dd, J_{H-H} = 18.1, 8.1 Hz, 1H, Py-CH*H'*N), 4.10 (bs, 2H, Pd(CH$_2$=CH_2)), 3.74 (bs, 1H, Ar-C*H*(CH_3)$_2$), 3.30 (bs, 1H, Ar-C*H*(CH_3)$_2$), 1.48–1.07 (m, 12H, Ar-CH(CH_3)$_2$; 2H, Pd-CH_2CH$_3$), 0.85 (m, 3H, Pd-CH_3); *C*-**5e'**: δ 7.90 (bs, 1H, Py H-6; 1H, Py H-4), 7.45 (m, 1H, Py H-3; 1H, Py H-5), 7.19 (bs, 2H, *m*-Ar; 1H, *p*-Ar), 6.79 (bs, 1H, N*H*-Ar), 5.08 (m, 1H, Py-C*H*H'N), 4.32 (bs, 1H, Py-CH*H'*N), 3.99 (bs, 1H, Ar-C*H*(CH_3)$_2$), 3.42 (bs, 1H, Ar-C*H*(CH_3)$_2$), 1.48–1.07 (m, 12H, Ar-CH(CH_3)$_2$; 2H, Pd-CH_2CH$_3$), 0.85 (m, 3H, Pd-CH_3). The Pd(CH_2=CH_2) signals of *C*-**5e'** are overlapped with free ethylene.

General procedure for copolymerization of ethylene-norbornene

Into a 600 mL Parr autoclave equipped with a magnetic stirring bar was placed norbornene (0.5–10 g) in dried CH_2Cl_2 (50 mL). The autoclave was sealed. Upon flush with ethylene gas several times, the ethylene gas was pressurized. The solution was stirred for 20 min in order to be saturated with ethylene gas. After release of ethylene pressure, the palladium complexes (0.06 mmol) were added, and then refilled with ethylene gas up to 21 bar. The mixture was stirred for 30 min, and the ethylene pressure was kept constant during the copolymerization runs. The reaction was quenched with venting the autoclave followed by adding 100 mL MeOH–HCl in 4:1 v/v ratio. The precipitated polymers were filtered from solution, washed with methanol and dried in vacuum oven at 80 °C overnight.

General procedures for the kinetic study determined by variable-temperature ^{1}H NMR spectroscopy

VT-NMR Insertion Kinetics for Ethylene

An NMR tube was charged with 0.7 mL of [(N^N)Pd(Me) (NCMe)]BF_4] solution ($2.5 \times 10^{-2} \sim 1.1 \times 10^{-3}$ M) in $CDCl_3$ and tetramethylsilane (TMS) as internal standard under nitrogen atmosphere, and then frozen with dry ice/acetone bath. After evacuation, desired amount of ethylene (4-8 mL) was injected by gastight syringe. The tube was warmed briefly around the melting point of $CDCl_3$ and shaken vigorously in order to adequately dissolve the ethylene. The sample was placed in a precooled NMR probe at desired temperature, and the decrease in intensity of Pd-Me was measured by using 1H NMR spectroscopy. Spectra were taken at intervals of 91-301 sec. The slope of the line defined by the Ln(integral of Pd-Me) signal vs time afforded the first-order rate constant, and the reaction was monitored for 2-4 half-lives.

Insertion Kinetics for Norbornene

An NMR tube was charged with 0.7 mL of [(N^N)Pd(Me) (NCMe)]BF_4 solution ($2.5 \times 10^{-2} – 1.1 \times 10^{-3}$ M) in $CDCl_3$ and tetramethylsilane (TMS) as internal standard, and then frozen with dry ice/acetone bath. Desired amount of norbornene (1.2 – 104.0 mg) was added directly. The sample was warmed briefly around the melting point of $CDCl_3$ and mixed by an iron stirrer. The tube was placed in a precooled NMR probe at desired temperature, and the decrease in intensity of Pd-Me for *cis*-isomers and Py H-4 or Py H-6 for *trans*-isomers were measured by using ^{1}H NMR spectroscopy. Spectra were taken at intervals of 46 – 301 sec. The slope of the line defined by the Ln(integral of Pd-Me, Py H-4 or Py H-6) signal vs time afforded the first-order rate constant, and the reaction was monitored for 2 – 4 half-lives.

Isomerization Kinetics

In a typical run, a single crystal of *C*-**2e** (3 mg, 4.9 μmol) was ground into powder, and then placed into an NMR tube with dry ice/acetone bath. $CDCl_3$ (0.7 mL) was added slowly at -78 °C in dry ice/acetone bath. The sample was warmed briefly and mixed by an iron stirrer around the melting point of $CDCl_3$. The tube was placed in a precooled NMR probe at desired temperature, and the decrease in intensity of Py H-6 for *C*-isomer was measured by using ^{1}H NMR spectroscopy. Spectra were taken at intervals of 46–301 sec. The slope of the line defined by the Ln(integral of Py H-6) signal vs time afforded the first-order rate constant, and the reaction was monitored for 2–4 half-lives.

Its rate constant k_{isom} could be evaluated as 5.48×10^{-5} at 248 K, 2.52×10^{-4} and 3.15×10^{-4} at 253 k, 2.71×10^{-4} at 258 K, 7.83×10^{-4} at 263 k, 1.48×10^{-3} at 265.5 K, 1.80×10^{-3} at 268 K, and 5.69×10^{-3} at 273 K.

X-ray crystallographic analysis

Diffraction data were measured on a Nonius CAD-4, SmartCCD, or Nonius KappaCCD diffractometer with graphite-monochromatized Mo K_α radiation (λ = 0.7103 Å). No significant decay was observed during the data collection. The data were processed on a PC using the SHELXTL refinement software package.[1] The structures were solved using the direct method and refined by full-matrix least-squares on the F^2 value.

All the non-hydrogen atoms were refined anisotropically. Hydrogen atoms were identified by calculation and refined using a riding mode, and their contributions to structure factors were included. Atomic scattering factors were taken from the International Tables of Crystallographic Data, Vol IV. Computing programs are from the NRC VAX package.[2]

Computational Details

All geometries of the reactants, intermediates, transition states and products of reactions were fully optimized by using the Gaussian 09 program,[3] and the B3LYP method with the LanL2DZ basis sets. No symmetry constrains were used for transition state optimization.[4–5] Saddle points were determined by relaxed potential energy surface (PES) scan for bond

distance or dihedral angle. Harmonic vibration frequency calculations were performed on all stationary points to identify the local minimum (without imaginary frequency) or transition state (with one imaginary frequency), and determinate zero-point energies (ZPE). The relative energies (E + ZPE), obtained from vibrational frequency analyses on optimized structures by single point calculations, were corrected for basis set superposition error (BSSE)[6] using the counterpoise method. Intrinsic reaction coordinate (IRC) calculations[7] were also performed to confirm the connectivity between the transition state and the designed intermediates. The enantiomers are omitted for discussion, because they generally show nearly identical energies in the preliminary calculations.. The chirality of the coordinated amino group is fixed in *R*-form for the comparing purpose.

General procedure for copolymerization of ethylene-norbornene

Into a 600 mL Parr autoclave equipped with a magnetic stirring bar was placed norbornene (0.5-10 g) in dried CH_2Cl_2 (50 mL). The autoclave was sealed. Upon flush with ethylene gas several times, the ethylene gas was pressurized. The solution was stirred for 20 min in order to be saturated with ethylene gas. After release of ethylene pressure, the palladium complexes (0.06 mmol) were added, and then refilled with ethylene gas up to 21 bar. The mixture was stirred for 30 min, and the ethylene pressure was kept constant during the copolymerization runs. The reaction was quenched with venting the autoclave followed by adding 100 mL MeOH-HCl in 4:1 v/v ratio. The precipitated polymers were filtered from solution, washed with methanol and dried in vacuum oven at 80 ^{o}C overnight.

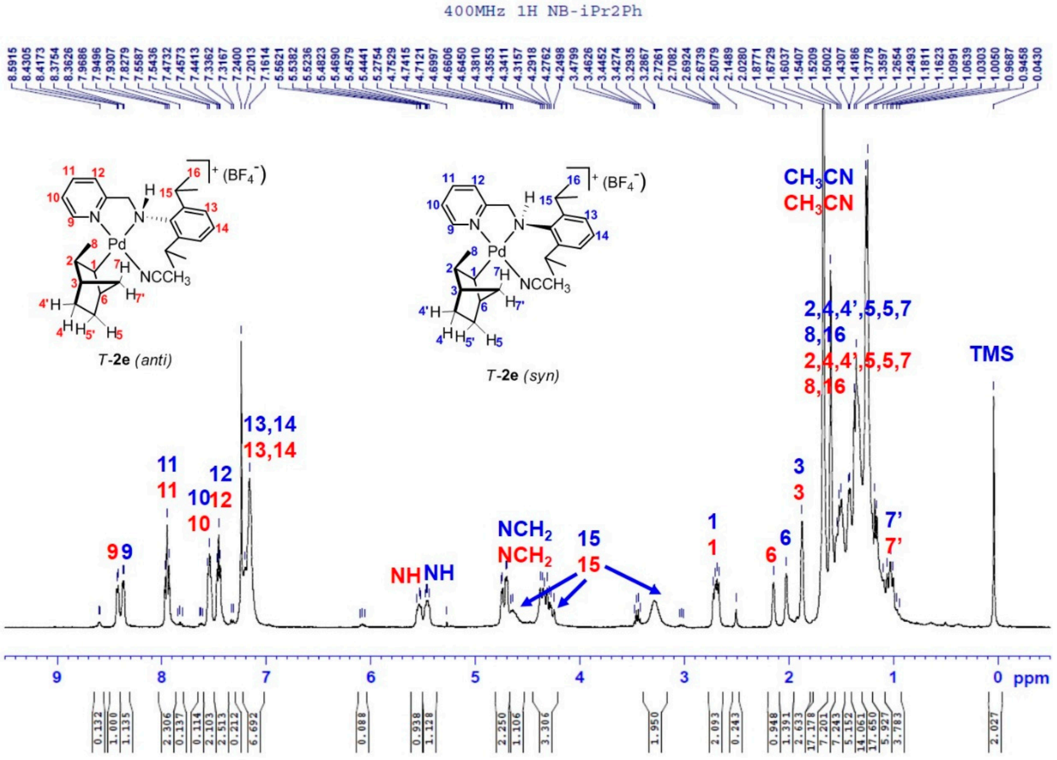

Figure S1. ^{1}H NMR spectrum and assignment for *T*-**2e** (room temperature, $CDCl_3$).

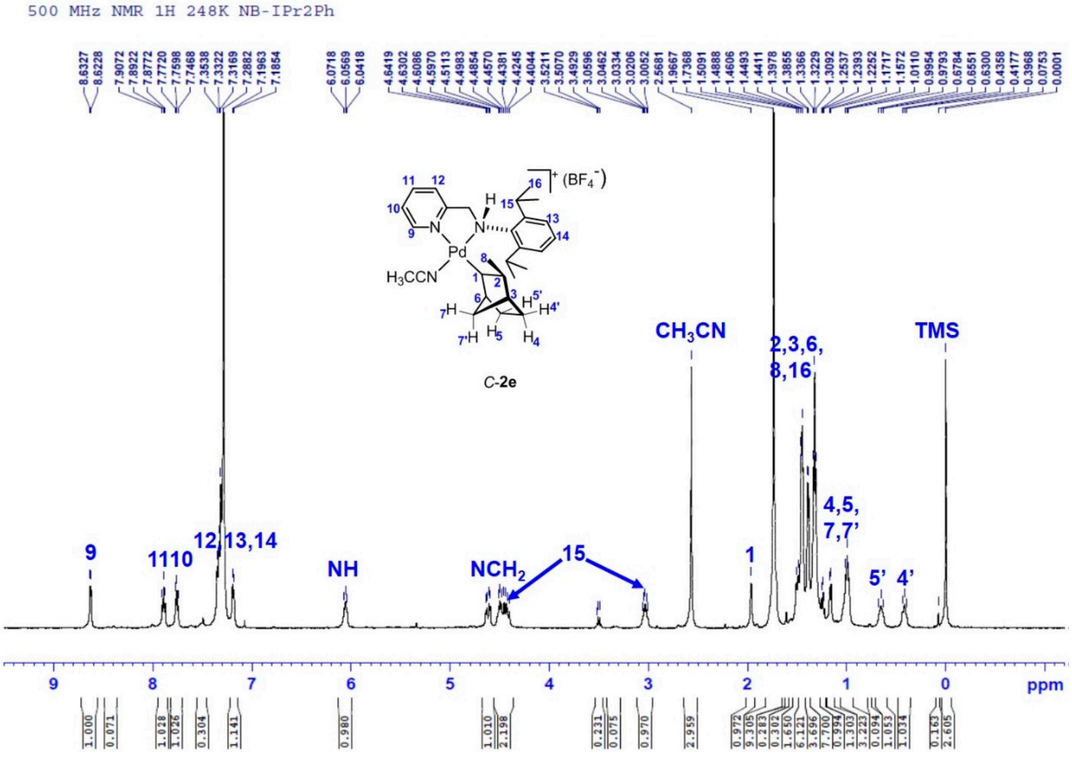

Figure S2. ^{1}H NMR spectrum and assignment for *C*-**2e** (248 K, $CDCl_3$).

Table S1. Selected bond distances (Å) and angles (°) for norbornyl palladium complexes.

{[^{t}BuHNCH$_2$(*o*-C$_6$H$_5$N)]Pd(C$_7$H$_{10}$Me)(NCMe)}(BF$_4$) (*T*-**2b**)							
Pd-N1	2.040 (3)	Pd-N2	2.195 (3)	Pd-C9	2.050 (3)	Pd-N3	2.009 (3)
N1-C6	1.361 (4)	N2-C7	1.463 (5)	C6-C7	1.505 (5)	N2-C21	1.508 (4)
N1-Pd-N2	81.5 (1)	C9-Pd-N3	93.7 (1)	Pd-N1-C6	113.7 (2)	Pd-N2-C7	103.0 (2)
N1-C6-C7	116.5 (3)	N2-C7-C6	112.2 (3)	C21-N2-Pd	119.2 (2)	C21-N2-C7	115.8 (3)
{[(2,6-^{i}Pr$_2$C$_6$H$_3$)HNCH$_2$(*o*-C$_6$H$_5$N)]Pd(C$_7$H$_{10}$Me)(NCMe)}(BF$_4$) (*C*-**2e**)							
Pd-N1	2.157 (2)	Pd-N2	2.116 (2)	Pd-C9	2.044 (2)	Pd-N3	2.007 (2)
N1-C6	1.335 (3)	N2-C7	1.492 (3)	C6-C7	1.449 (3)	N2-C21	1.466 (3)
N1-Pd-N2	80.20 (7)	C9-Pd-N3	92.63 (9)	Pd-N1-C6	113.9 (2)	Pd-N2-C7	100.3 (1)
N1-C6-C7	116.9 (2)	N2-C7-C6	112.8 (2)	C21-N2-Pd	120.5 (1)	C21-N2-C7	111.2 (2)

Table S2. X-ray crystal parameters and data collection for norbornyl palladium complexes.

**Compound**	*T*-**2b**	*C*-**2e**
Formula	$C_{20}H_{32}BF_4N_3Pd$	$C_{28}H_{40}BF_4N_3Pd$
Formula wt	507.70	611.84
Crystal size / mm	0.25×0.20×0.15	0.20×0.15×0.10
Crystal system	Monoclinic	Monoclinic
Space group	$P2_1/c$	$P2_1/c$
a / Å	12.1370(4)	10.7045(2)
b / Å	9.6243(3)	19.0549(3)
c / Å	19.6988(5)	14.6718(2)
α / °	90	90
β / °	102.741(3)	95.208(1)
γ / °	90	90
V / Å^3	2244.4(1)	2980.30(8)
Z	4	4
ρ_{calcd} / Mg·m^{-3}	1.503	1.364
F(000)	1040	1264
T / K	295(2)	295(2)
μ / mm^{-1}	0.870	0.668
Transmission	0.865-0.730	1.00000-0.91567
θ range / °	3.00-27.50	2.87-27.50
h, k, l	±15, ±12, ±25	±13, ±24, ±19
Reflections collected	26632	34969
Independent reflections / R_{int}	5146/0.0286	6843/0.0279
Data / restraints	5146/0	6843/0
Parameters	266	338
R_1 / wR_2 [I > 2σ(I)]	0.0387/0.1276	0.0313/0.0971
R_1 / wR_2 (all data)	0.0490/0.1414	0.0459/0.1052
Goodness of fit on F^2	1.090	0.764
Largest diff. peak and hole, eÅ^{-3}	1.599 and -0.533	0.561 and -0.375

Table S3. E-N Copolymerization at different comonomer ratios by using catalyst **1e**[a]

Entry	NB$_{feed}$ (g)	NB$_{feed}$[b] (mol%)	Yield (g)	Activity (kg mol^{-1}h^{-1})	Mw[c] (x 10^3)	PDI[c]
1	0.5	6	0.51	17	3.4	1.8
2	1	11	0.84	28	8.5	1.7
3	3	28	1.59	53	16.9	1.9
4	5	39	1.47	49	23.0	2.1
5	7	47	1.41	47	18.6	2.6
6	10	56	1.18	39	26.6	4.8

Entry	T$_g$[d] (℃)	NB$_{coc}$[e] (mol %)	Single[f] (mol %)	Diads[f] (mol %)	Triads[f] (mol %)	Alternating[g] (mol %)
1	85	43.0	37.7	4.4	1.8	81.0
2	119	49.0	38.7	6.1	4.2	86.3
3	135	51.3	36.4	9.3	5.6	85.8
4	156	55.1	26.3	14.4	14.4	76.6
5	162	55.7	28.3	13.8	13.6	79.5
6	177	58.0	19.9	15.5	22.6	70.4

[a] Reaction conditions: 0.06 mmol of catalysts, 21 bar of ethylene, 50 mL of CH_2Cl_2, 30 min, room temperature. [b] Norbornene content in the feed. [c] Determined by GPC using polystyrene as standards. [d] Determined by DSC. [e] Norbornene content in the copolymer, determined by ^{13}C NMR. [f] Norbornene content of single norbornene unit or blocks in the copolymer, determined by ^{13}C NMR. [g] Determined by ^{13}C NMR, alternating mol % = 2 × single norbornene mol% + norbornene diads mol % + 2/3 × norbornene triads mol%.

Fineman-Ross Plot

The copolymer compositions are determined according to the literature.[9]

$$\text{Pd-N-polymer} + \text{N} \xrightarrow{k_{NN}} \text{Pd-N-N-polymer} \qquad (3\text{-}6)$$

$$\text{Pd-N-polymer} + \text{E} \xrightarrow{k_{NE}} \text{Pd-N-E-polymer} \qquad (3\text{-}7)$$

$$\text{Pd-E-polymer} + \text{N} \xrightarrow{k_{EN}} \text{Pd-E-N-polymer} \qquad (3\text{-}8)$$

$$\text{Pd-E-polymer} + \text{E} \xrightarrow{k_{EE}} \text{Pd-E-E-polymer} \qquad (3\text{-}9)$$

Where Pd-N-polymer and Pd-E-polymer are the growing chain with norbornene and ethylene as the last inserted monomer, and the reactivity ratios are defined as $r_1 = k_{NN}/k_{NE}$ and $r_2 = k_{EE}/k_{EN}$. The r_1 represents the ratio of rate constants between the successive norbornene insertions and norbornene insertion followed by ethylene, and the r_2 represents the ratio of rate constants between the successive ethylene insertions and ethylene insertion followed by norbornene. By using quasi-steady-state assumption for propagation, the copolymer composition equation can be derived as:

$$\left(\frac{[N]}{[E]}\right)_{polymer} = \frac{[N]}{[E]} \frac{\left(1 + r_1 \frac{[N]}{[E]}\right)}{\left(r_2 + \frac{[N]}{[E]}\right)} \qquad (3\text{-}10)$$

where $([E]/[N])_{polymer}$ and ([E]/[N]) are the ethylene and norbornene molar ratios in the copolymer and the bulk reaction solution. Futher replacement of $([N]/[E])_{polymer}$ and ([N]/[E]) to f and F, the following Fineman-Ross equation can be derived as:

$$\frac{(f-1)}{f} F = r_1 \frac{F^2}{f} - r_2 \qquad (3\text{-}11)$$

According to the N/E feeding ratios and norbornene calculated from ^{13}C NMR spectra in Table S1, the values of F and f in Eq. (3-11) are collected in Table S2. The Fineman-Ross data of E-N copolymerization catalyzed by **1e** give linear relationships between F^2/f and $F(f-1)/f$ as shown in Figure S1. Such data give the $r_1 = k_{EE}/k_{EN} = 0.013$, $r_2 = k_{NN}/k_{NE} = 0.31$ and $r_1 \times r_2 = 0.004$. The product of $r_1 \times r_2$ is significantly smaller than 1.0, indicating the consecutive hetero-olefin insertions are faster than the consecutive homo-olefin insertions.

Table S4. Fineman-Ross data of E-N copolymerization catalyzed by **1e**

NB (g)	NB Conc. (M)	E Conc. (M)	F ([N]/[E])	NB % (^{13}C NMR)	f $(([N]/[E])_{polymer})$	F^2/f	$F(f\text{-}1)/f$
0.5	0.11	1.66	0.066265	43.0	0.754386	0.005821	-0.02157
1	0.21	1.66	0.126506	49.0	0.960784	0.016657	-0.00516
3	0.64	1.66	0.385542	51.3	1.053388	0.141109	0.01954
5	1.06	1.66	0.638554	55.1	1.227171	0.332269	0.118208
7	1.47	1.66	0.885542	55.7	1.257336	0.623687	0.181242
10	2.13	1.66	1.283133	58.0	1.380952	1.192243	0.353968

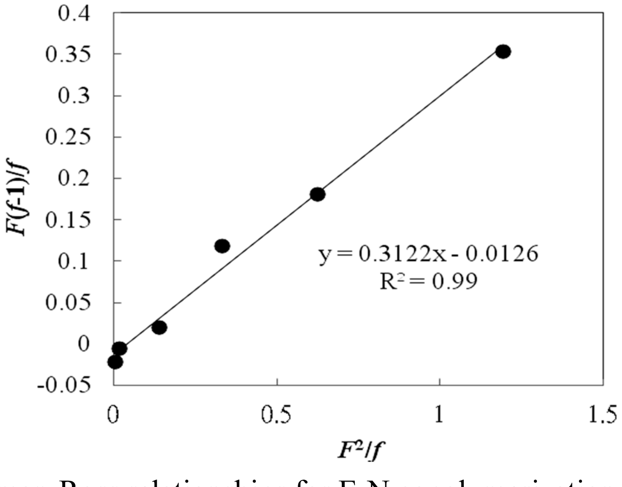

Figure S3. Fineman-Ross relationships for E-N copolymerization catalyzed by **1e**.

Determination of norbornene content and alternating percentage for copolymers

The norbornene content, X_{NB} was evluated with use of ^{13}C NMR integrations as designated in the following table according to Eq. (1) used by Kaminsky.[10]

$$X_{\mathrm{NB}} = \frac{I(\mathrm{A})}{I(\mathrm{B}) + I(\mathrm{C}) + I(\mathrm{D}) - 1.5\, I(\mathrm{A})} \qquad (1\text{-}1)$$

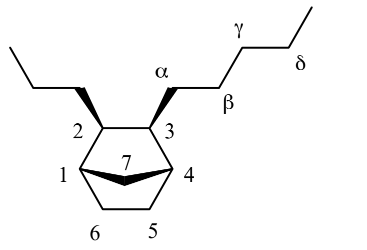

cis-2,3-*exo* insert norbornene unit

Table S5. Assignments of ethylene/norbornene copolymers in ^{13}C NMR spectra

Singnal area	δ (^{13}C NMR) (ppm)	Asignments
A	51 - 45	C2, C3
B	43 - 37	C1, C4
C	37 – 32.3	C7
D	32.3 - 28	C5, C6, C_α, C_β, C_γ, C_δ

The alternating percentage in a copolymer may be calculated according to Eq. (1-2).

$$Alternating\% = 2 \times single\ NB\ mol\ \% + NB\ Diads\ mol\ \% + 2/3 \times NB\ Triads\ mol\ \% \qquad (1\text{-}2)$$

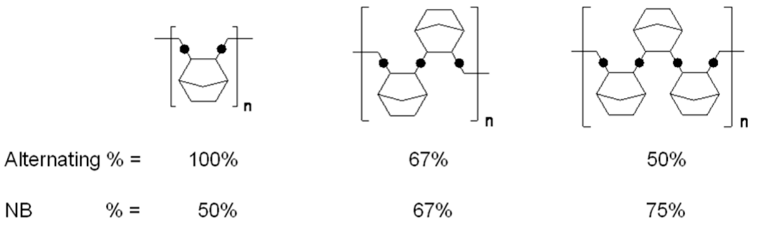

Figure S4. Examples of ethylene/norbornene copolymer and block copolymers

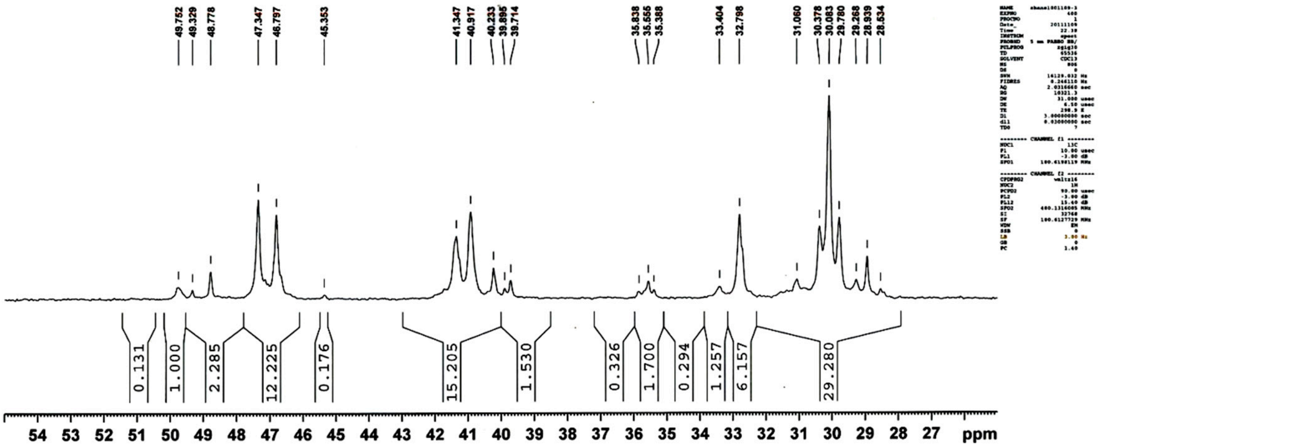

Figure S5. E-N copolymer catalyzed by **1a** with 1 g of norbornene feeding.

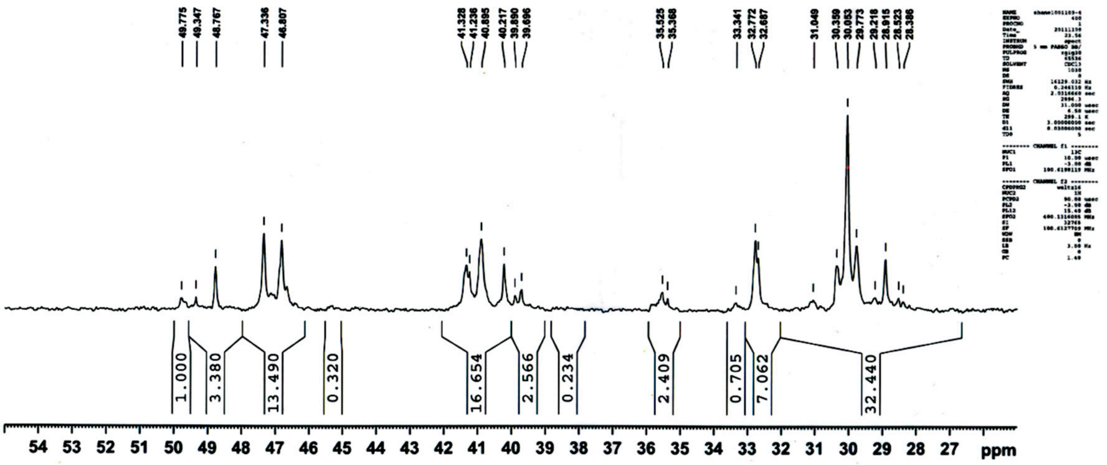

Figure S6. E-N copolymer catalyzed by **1b** with 1 g of norbornene feeding.

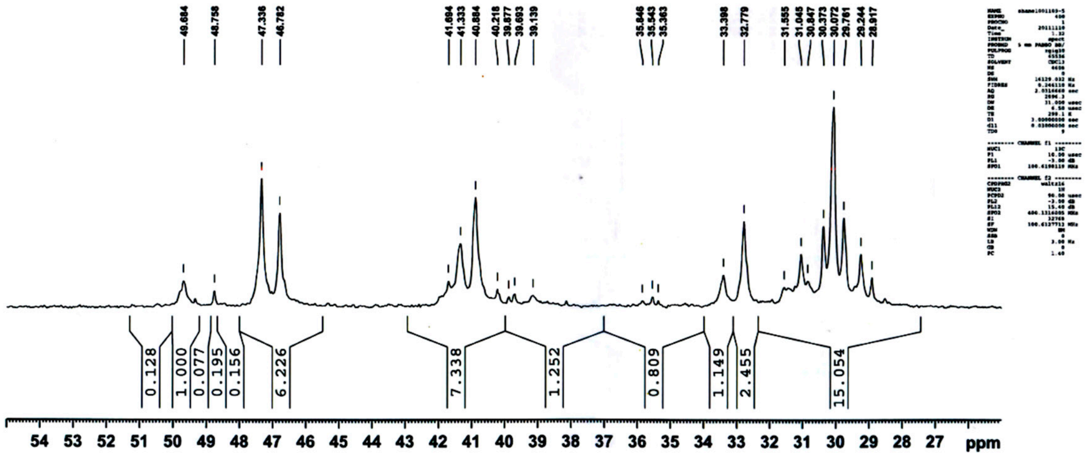

Figure S7. E-N copolymer catalyzed by **1c** with 1 g of norbornene feeding.

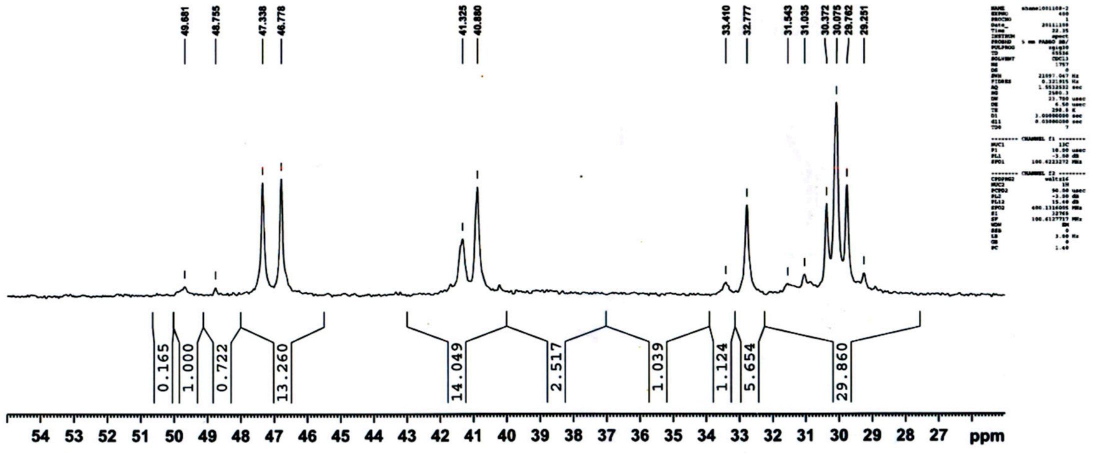

Figure S8. E-N copolymer catalyzed by **1d** with 1 g of norbornene feeding.

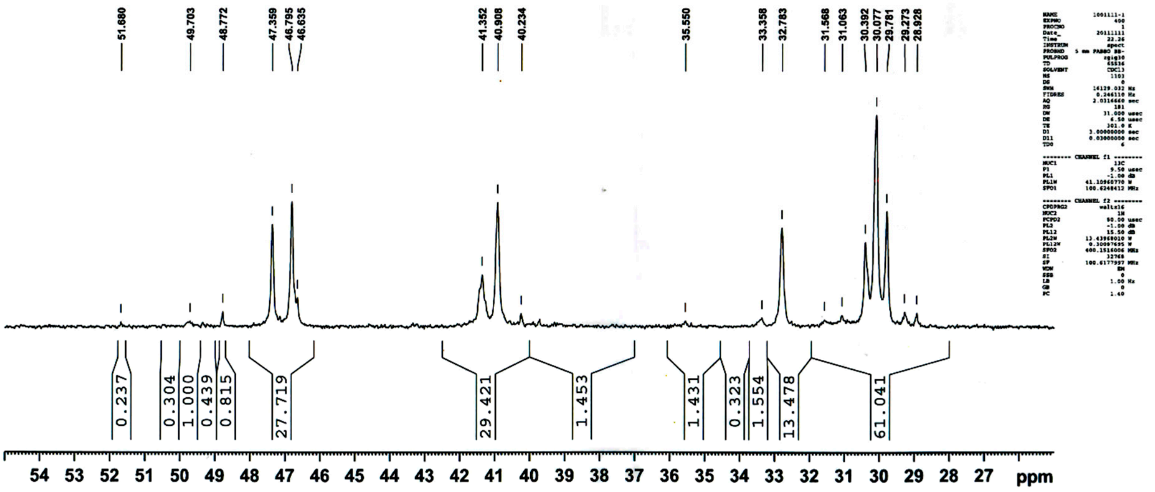

Figure S9. E-N copolymer catalyzed by **1e** with 0.5 g of norbornene feeding

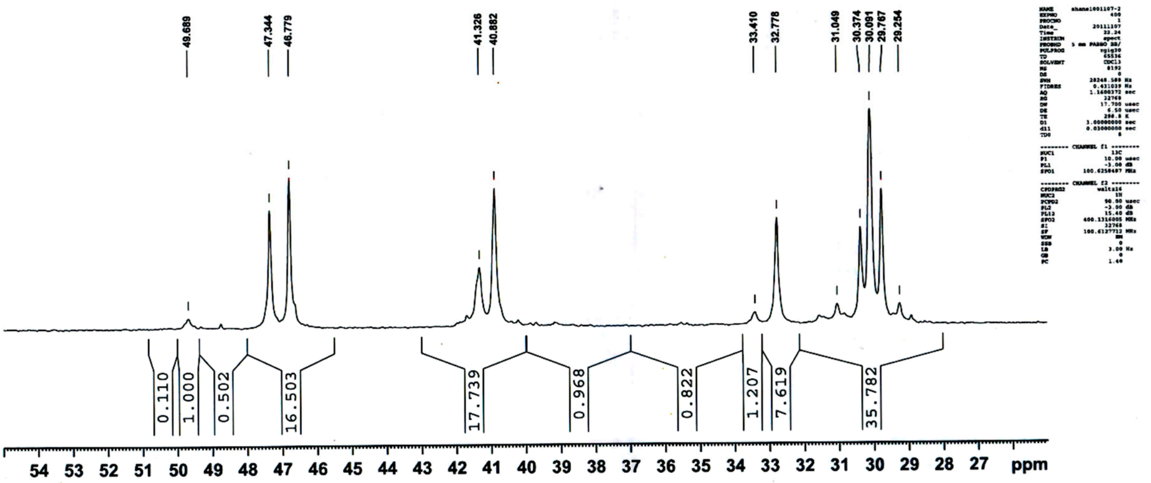

Figure S10. E-N copolymer catalyzed by **1e** with 1 g of norbornene feeding.

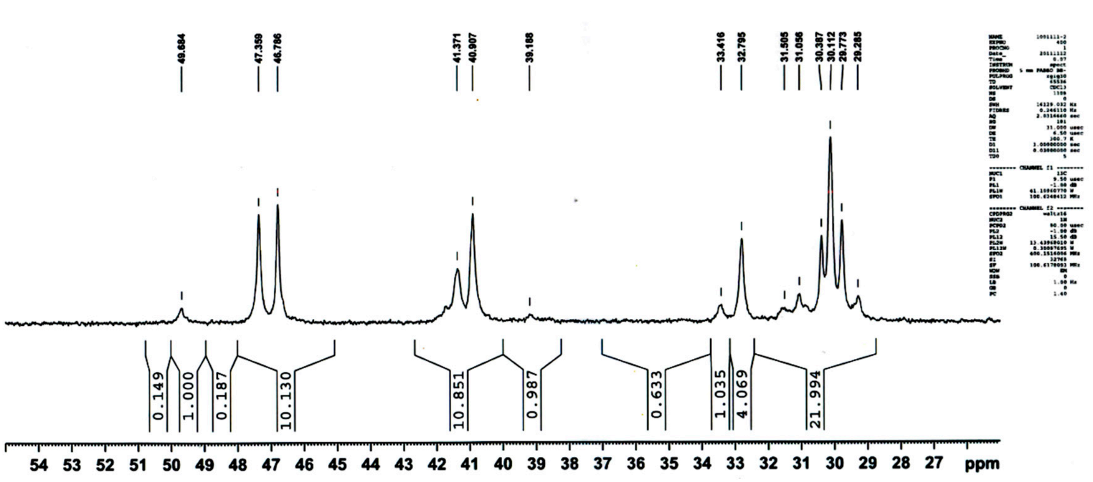

Figure S11. E-N copolymer catalyzed by **1e** with 3 g of norbornene feeding.

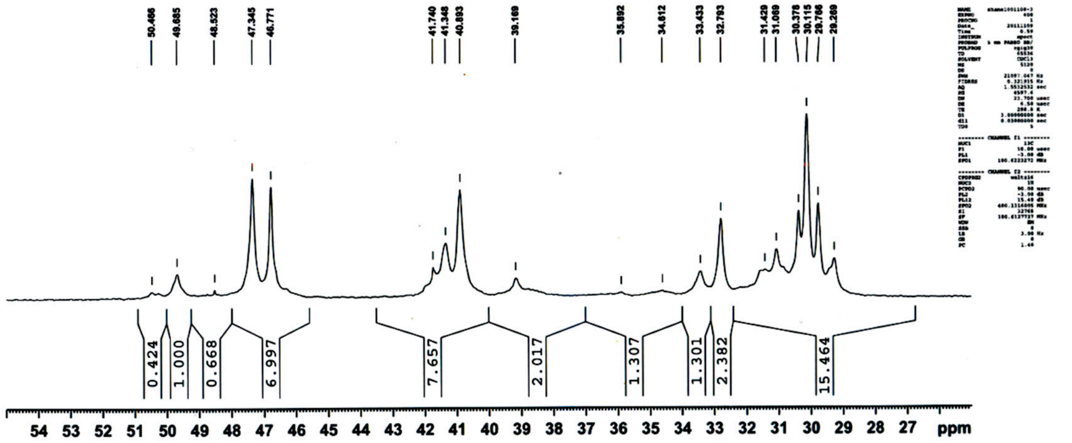

Figure S12. E-N copolymer catalyzed by **1e** with 5 g of norbornene feeding.

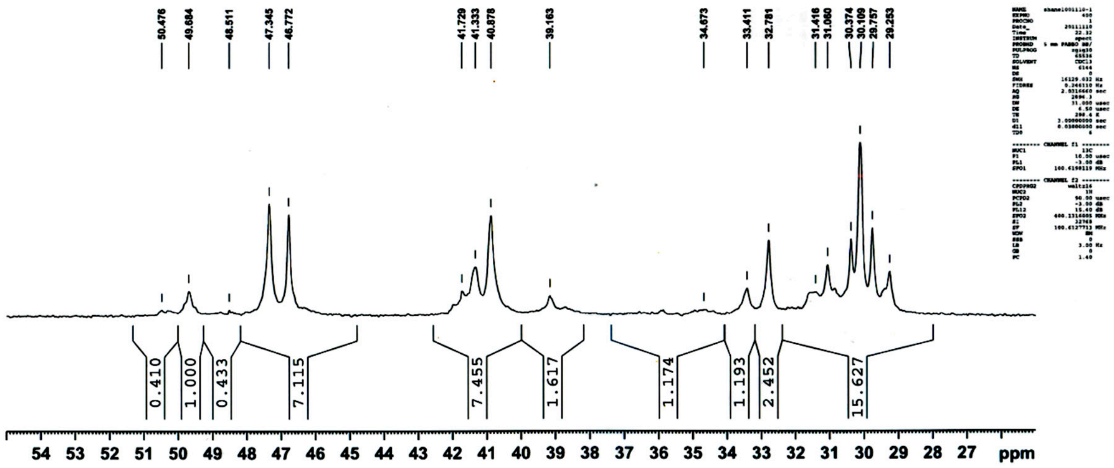

Figure S13. E-N copolymer catalyzed by **1e** with 7 g of norbornene feeding.

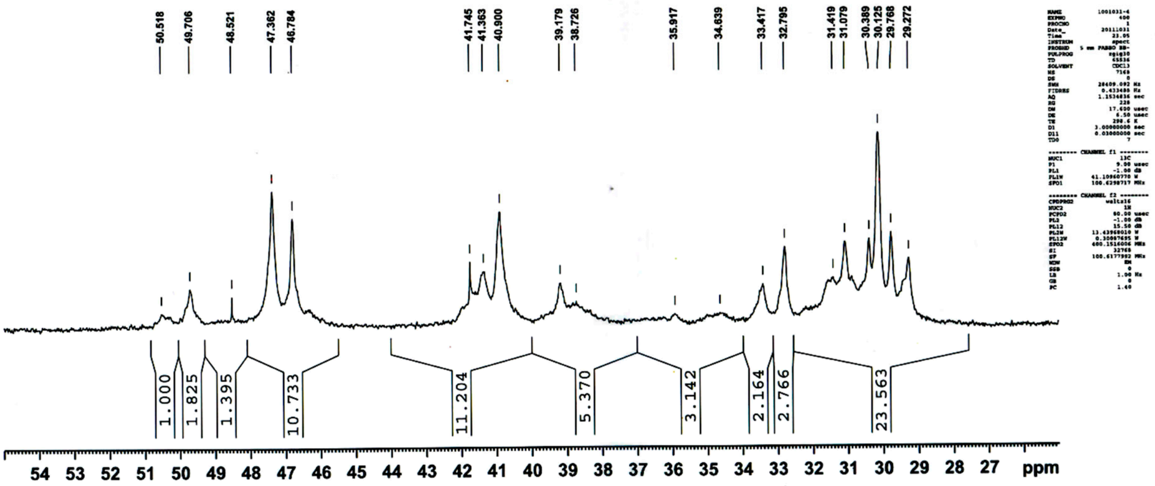

Figure S14. E-N copolymer catalyzed by **1e** with 10 g of norbornene feeding.

Table S6. E-N Copolymerization by using catalyst **1b** and **2b**.[a]

**Cat.**	**NB$_{feed}$** (g)	**NB$_{feed}$**[b] (mol%)	**Yield** (g)	**Activity** (kg mol^{-1}h^{-1})	**Mw**[c] (x 10^3)	**PDI**[c]	**T$_g$**[d] (℃)	**NB$_{coc}$**[e] (mol %)
**1b**	10	56	1.06	26	5.6	1.8	117	43.0
**2b**	10	56	0.54	18	3.6	1.9	127	46.0

[a] Reaction conditions: 0.06 mmol of catalysts, 21 bar of ethylene, 50 mL of CH_2Cl_2, 30 min, room temperature. [b] Norbornene content in the feed. [c] Determined by GPC using polystyrene as standards. [d] Determined by DSC. [e] Norbornene content in the copolymer, determined by ^{13}C NMR.

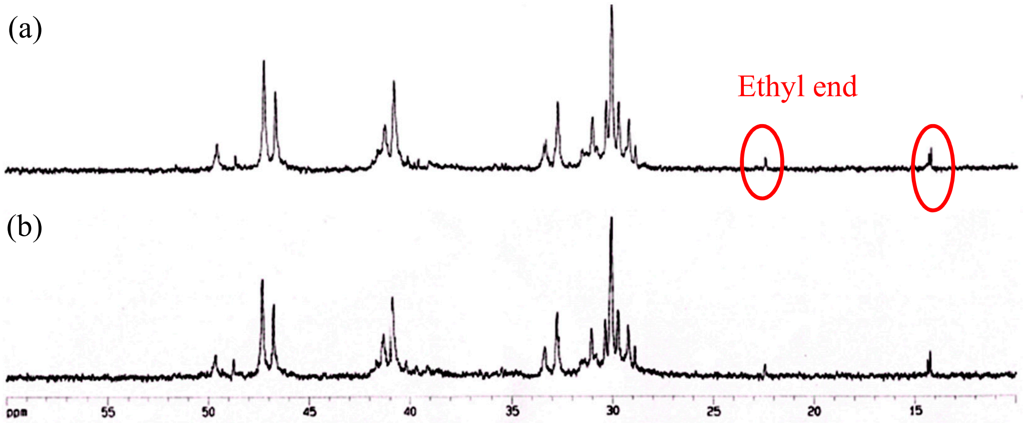

Figure S15. ^{13}C NMR spectra of E-N copolymer catalyzed by (a) **1b** and (b) **2b** show the same ethyl end group, which may be attributed to the chain transfer of E-bounded species.

Table S7. Norbornene blocks of E-N copolymers catalyzed by 1a-1e.[a]

**Cat**	**Act.**[b]	**NB$_{coc}$**[c] **(mol %)**	**Single**[c] **(mol %)**	**Diads**[c] **(mol %)**	**Triads**[c] **(mol %)**	**Alter.**[c] **(mol %)**
**1a**	14	44.0	33.7	6.9	3.4	76.6
**1b**	5	42.9	39.0	3.9	0	81.9
**1c**	6	47.5	26.4	12.4	8.7	71.0
**1d**	27	48.1	34.8	6.9	6.4	80.8
**1e**	28	49.0	38.7	6.1	4.2	86.3

[a] Reaction conditions: 0.06 mmol of catalysts, 21 bar of ethylene, 50 mL of CH_2Cl_2, 30 min, room temperature. [b]Activity = kg (COC)mol^{-1}(Pd) h^{-1}. Determined by 13C NMR.

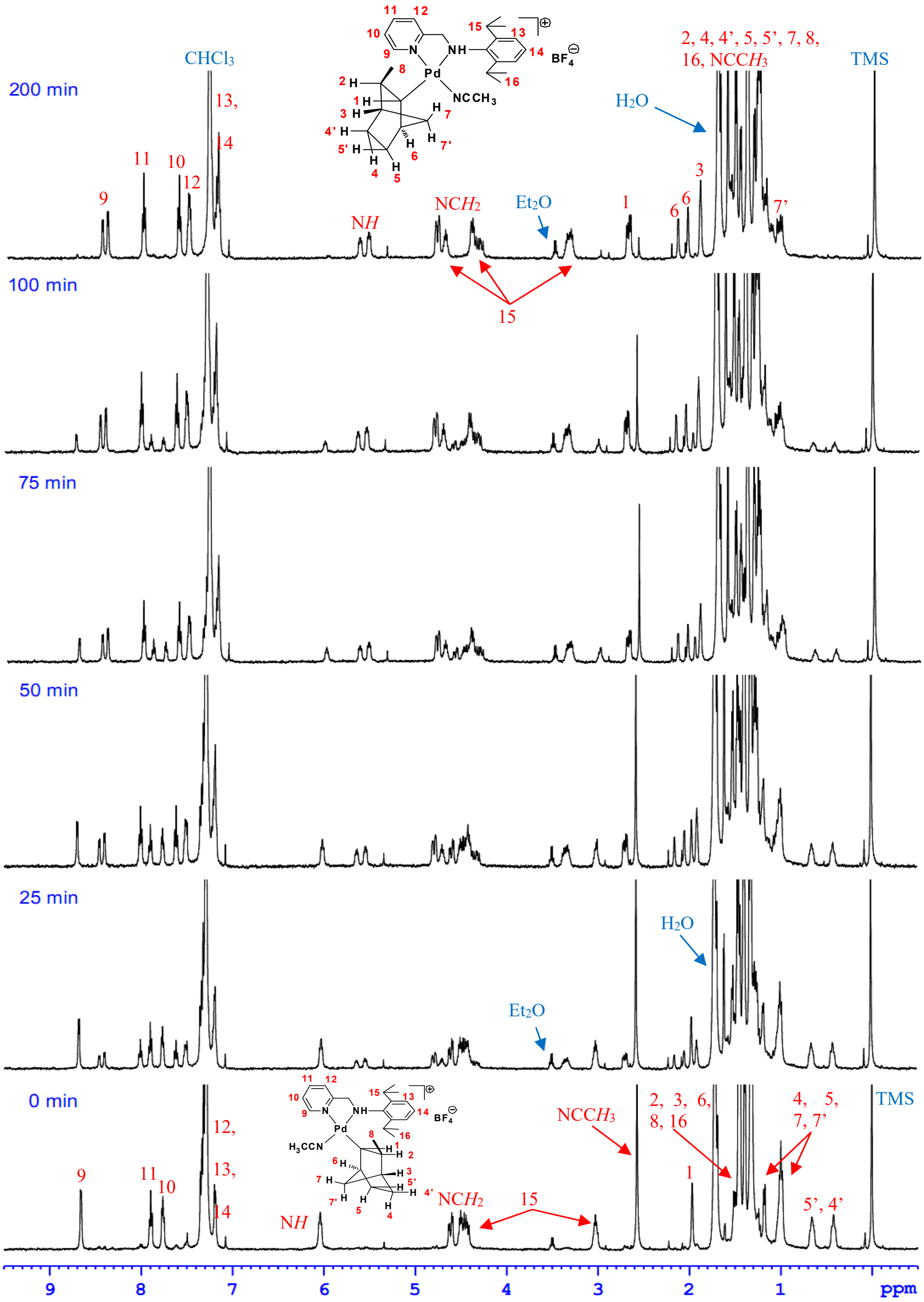

Figure S16. *C*-to-*T* isomerization of *C*-**2e** at 253 K.

Table S8. Kinetic data of *cis* to *trans* isomerization for *C*-**2e**.

Temp (K)	[Complex] (M)	Interval (s)	k_{isom} (s^{-1})	$\tau_{1/2\ isom}$ (s)	Half-Lives
273	6.07×10^{-3}	46	5.69×10^{-3}	122	11.3
268	4.97×10^{-3}	60	1.80×10^{-3}	385	3.9
265.5	7.17×10^{-3}	91	1.48×10^{-3}	468	4.5
263	1.44×10^{-2}	91	7.83×10^{-4}	855	2.9
258	6.35×10^{-3}	455	2.71×10^{-4}	2557	5.3
253 (1st)	7.17×10^{-3}	301	3.15×10^{-4}	2200	6.8
253 (2nd)	6.07×10^{-3}	301	2.52×10^{-4}	2750	5.7
248	6.07×10^{-3}	903	5.48×10^{-5}	12646	2.7

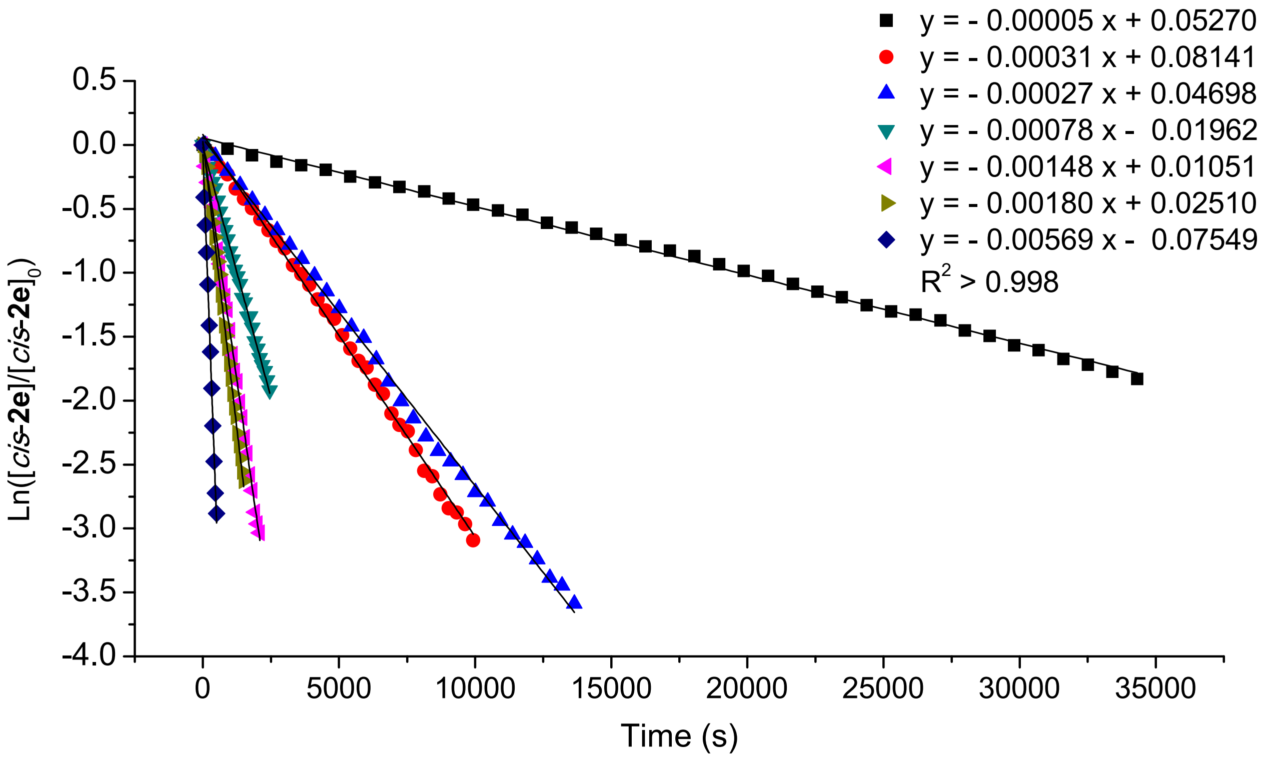

Figure S17. First-order kinetic plots for the *C*-to-*T* isomerization reaction of *C*-**2e** at various temperatures in $CDCl_3$ (■, 248 K; ●, 253 K; ▲, 258 K; ▼, 263 K; ◀, 265.5 K; ▶, 268 K; ◆, 273 K).

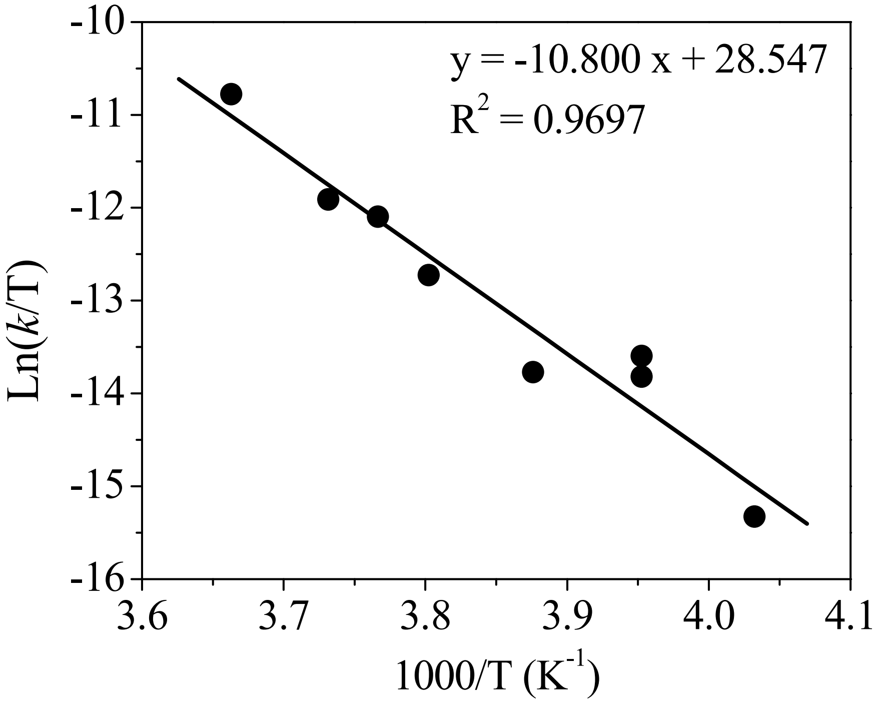

Figure S18. Eyring relationship for the isomerization reaction of *C*-**2e** in $CDCl_3$.

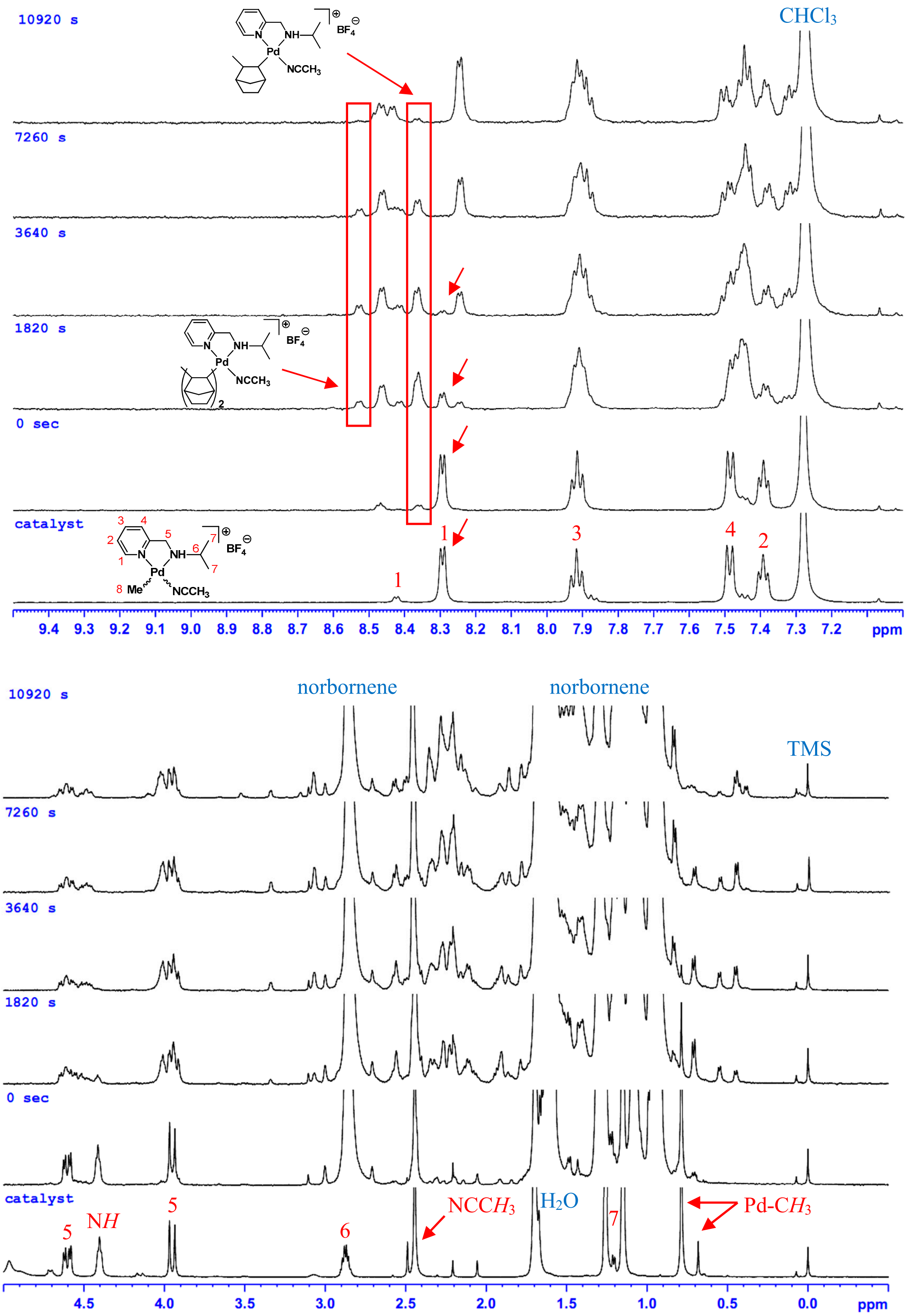

Figure S19. Norbornene insertion reaction for **1a** at 263 K with the range 7.0 - 9.5 ppm (up) and -0.5 – 5.0 ppm (down) in ^{1}H NMR spectra.

Table S9. Kinetic data for the k_{obs} of norbornene insertion reaction in $CDCl_3$.

Temp. (K)	Cat	R	[Cat] (10^{-3} M)	[N][a] (10^{-2} M)	[N]/[Cat]	k_{obs} ($\times 10^{-4}$ s^{-1})[b] Cis	k_{obs} ($\times 10^{-4}$ s^{-1})[b] Trans	k_{cis} / k_{trans}
263	**1a**	^{i}Pr	5.3	9.9	28.7	-	8.69 (5.0)	
263	**1b**	^{t}Bu	24.9	205.0	82.2	-	2.40 (3.6)	
263	**1c**	Ph	2.0	4.7	23.2	-	51.60 (4.0)	
263	**1d**	2,6-$Me_2C_6H_3$	1.1	1.8	16.7	134.00 (4.8)	21.80 (2.8)	7.8
273	**1e**	2,6-$^{i}Pr_2C_6H_3$	3.3	10.8	32.5	-	23.80 (3.5)	
268	**1e**	2,6-$^{i}Pr_2C_6H_3$	3.3	8.2	24.6	-	12.10 (3.8)	
263	**1e**	2,6-$^{i}Pr_2C_6H_3$	3.4	3.6	10.8	-	5.47 (4.2)	
263	**1e**	2,6-$^{i}Pr_2C_6H_3$	3.4	8.1	24.2	-	6.19 (3.9)	
263	**1e**	2,6-$^{i}Pr_2C_6H_3$	3.4	21.0	62.6	-	10.10 (3.8)	
263	**1e**	2,6-$^{i}Pr_2C_6H_3$	3.4	45.1	134.5	-	12.90 (4.7)	
263	**1e**	2,6-$^{i}Pr_2C_6H_3$	3.4	87.4	260.9	-	23.80 (4.3)	
263	**1e**	2,6-$^{i}Pr_2C_6H_3$	1.7	3.6	21.0	110.00 (4.1)	-	
258	**1e**	2,6-$^{i}Pr_2C_6H_3$	3.3	10.3	31.1	109.00 (5.0)	2.35 (3.5)	46.5
253	**1e**	2,6-$^{i}Pr_2C_6H_3$	3.4	8.9	26.3	41.30 (5.5)	1.29 (2.7)	32.0
243	**1e**	2,6-$^{i}Pr_2C_6H_3$	3.3	9.1	28.0	16.80 (4.2)	-	
238	**1e**	2,6-$^{i}Pr_2C_6H_3$	3.4	9.3	27.2	9.68 (5.0)	-	
233	**1e**	2,6-$^{i}Pr_2C_6H_3$	3.3	13.3	40.5	12.90 (4.9)	-	
233	**1e**	2,6-$^{i}Pr_2C_6H_3$	3.3	11.1	33.7	10.80 (4.8)	-	
233	**1e**	2,6-$^{i}Pr_2C_6H_3$	3.3	6.6	20.1	7.11 (4.6)	-	
228	**1e**	2,6-$^{i}Pr_2C_6H_3$	3.4	10.5	31.3	2.50 (4.2)	-	

[a] The [N] were calculated from ^{1}H NMR spectra. [b] The numbers of half-lives are given in the parentheses.

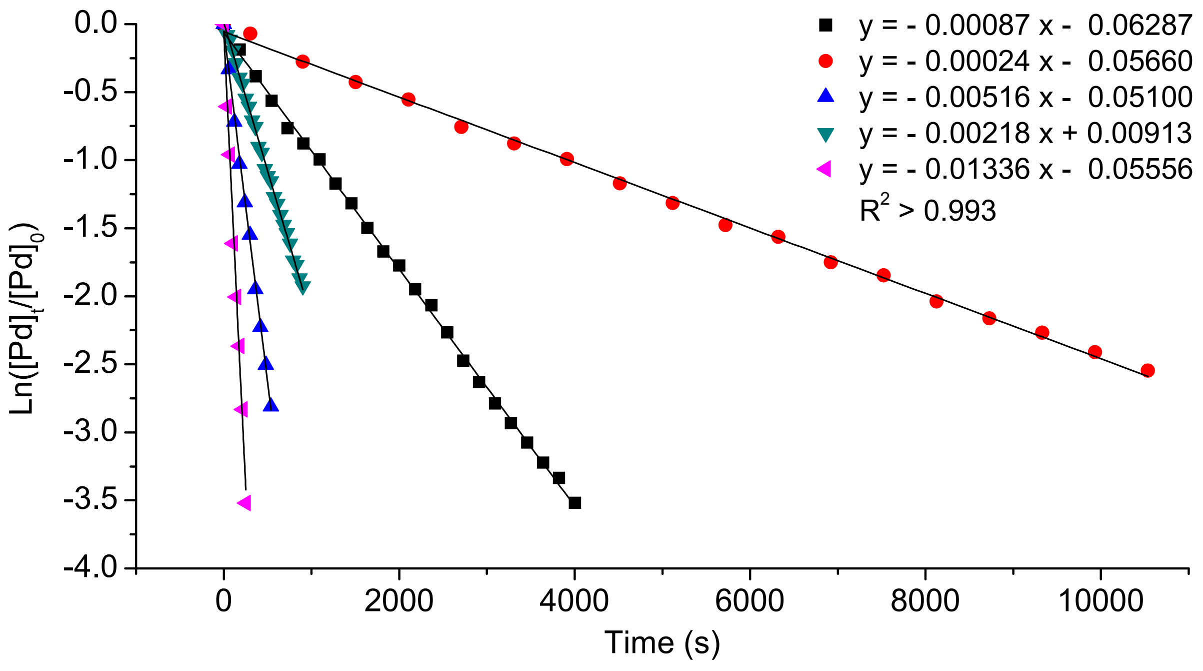

Figure S20. Pseudo-first-order kinetic plots for the norbornene insertion reactions of various catalysts at 263 K in $CDCl_3$ (■, *T*-**1a**; ●, *T*-**1b**; ▲, *T*-**1c**; ▼, *T*-**1d**; ◀, *C*-**1d**).

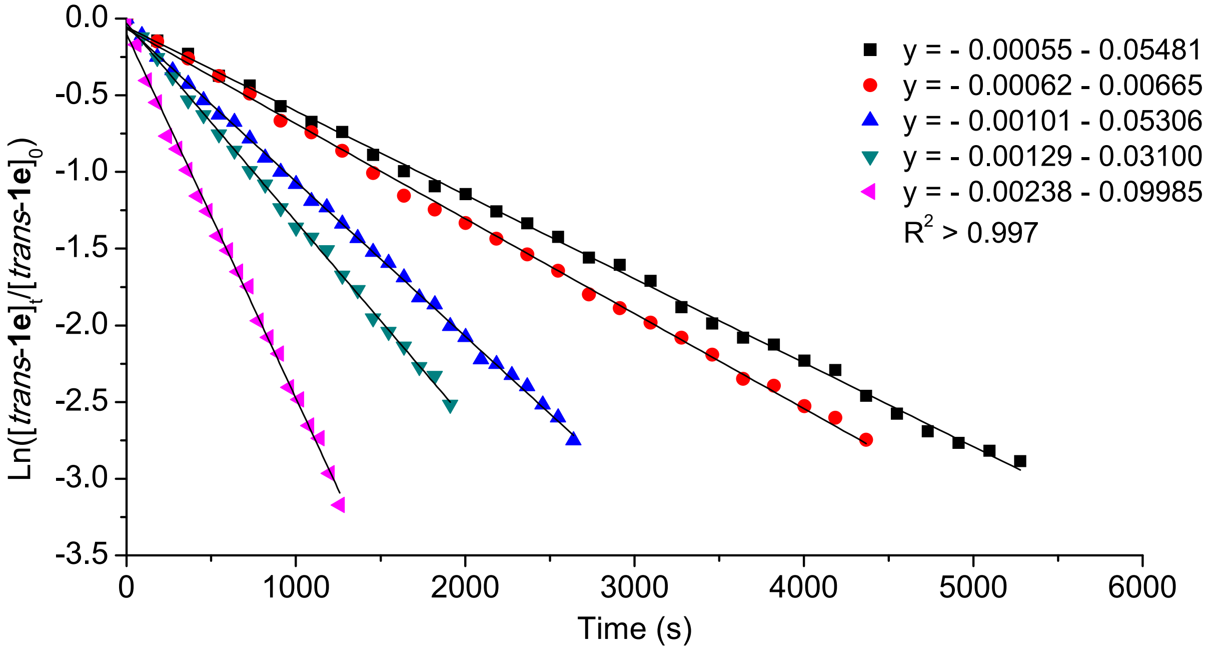

Figure S21. Pseudo-first-order kinetic plots for the norbornene insertion reactions of *T*-**1e** with various [N]/[Pd] ratios at 263 K in $CDCl_3$ ([N]/[Pd]: ■, 10.8; ●, 24.2; ▲, 62.6; ▼, 134.5; ◀, 260.9).

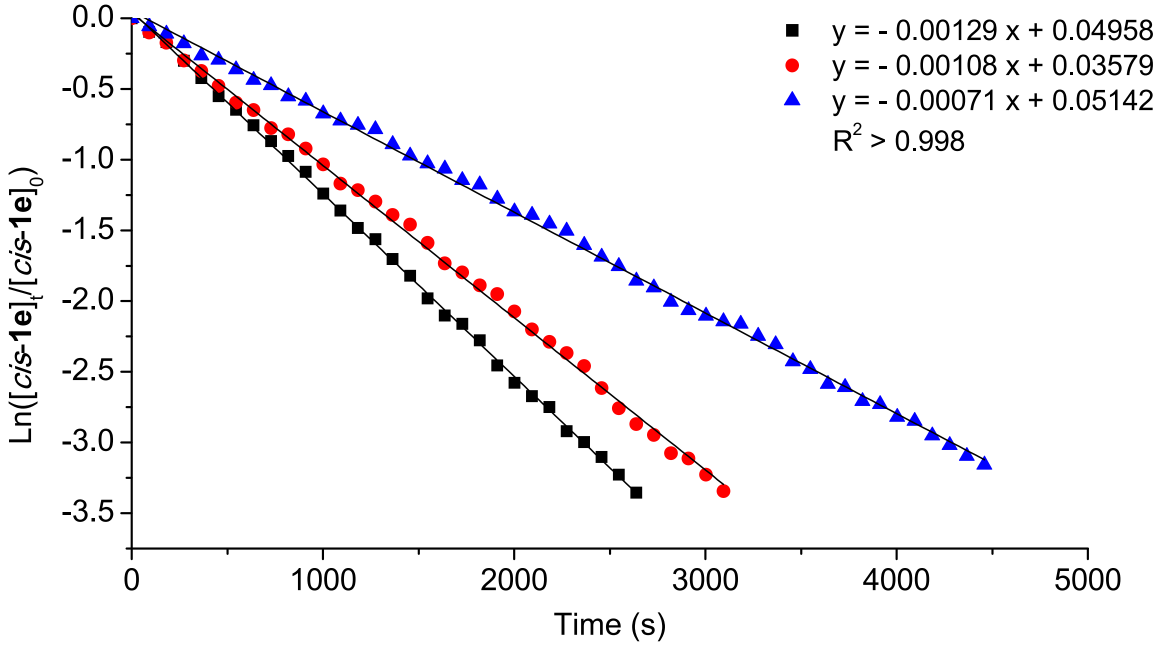

Figure S22. Pseudo-first-order kinetic plots for the norbornene insertion reactions of *C*-**1e** with various [N]/[Pd] ratios at 233 K in $CDCl_3$ ([N]/[Pd]: ■, 40.5; ●, 33.7; ▲, 20.1).

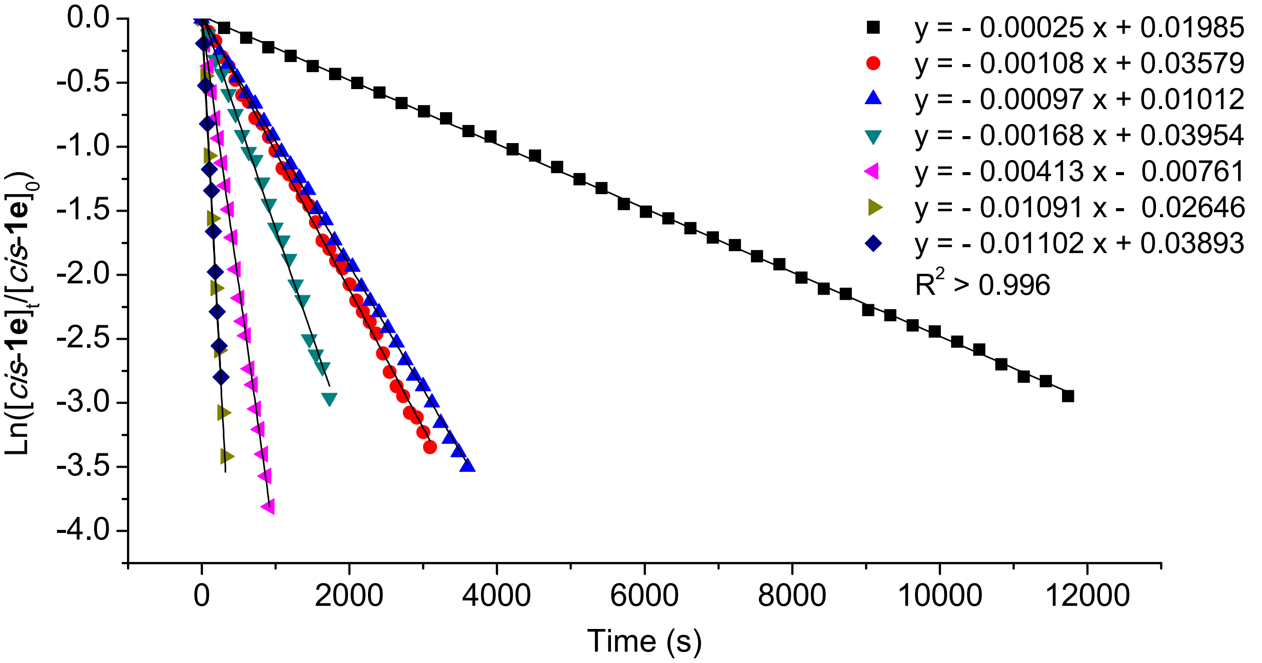

Figure S23. Pseudo-first-order kinetic plots for the norbornene insertion reactions of *C*-**1e** at various temperatures in $CDCl_3$ (■, 228 K; ●, 233 K; ▲, 238 K; ▼, 243 K; ◀, 253 K; ▶, 258 K; ◆, 263 K).

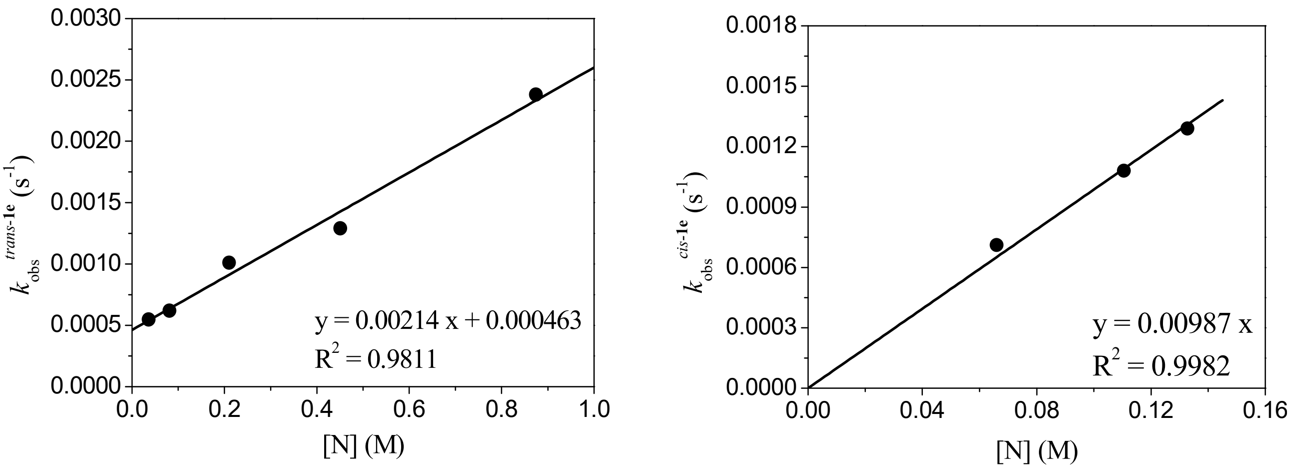

Figure S24. Relationships between k_{obs} and [N] for *T*-**1e** at 263 K (left) and *C*-**1e** at 233 K (right).

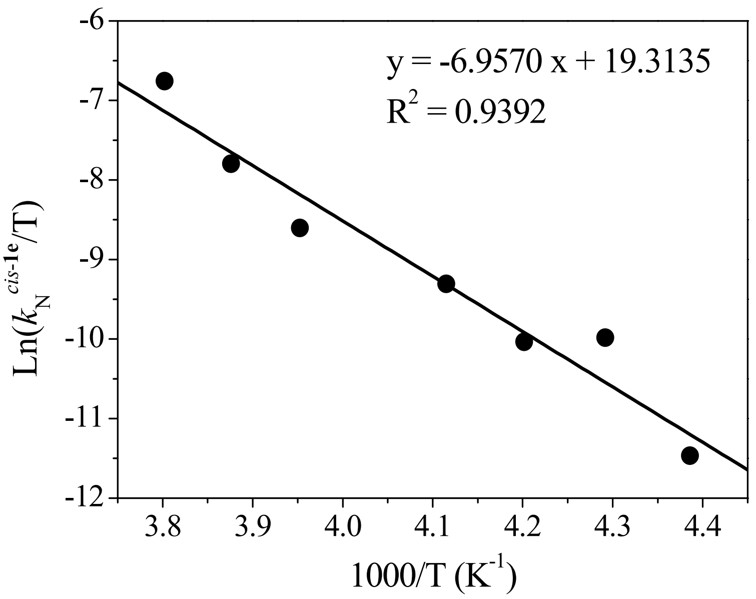

Figure S25. Eyring relationship for the norbornene insertion reaction of *C*-**1e** in $CDCl_3$.

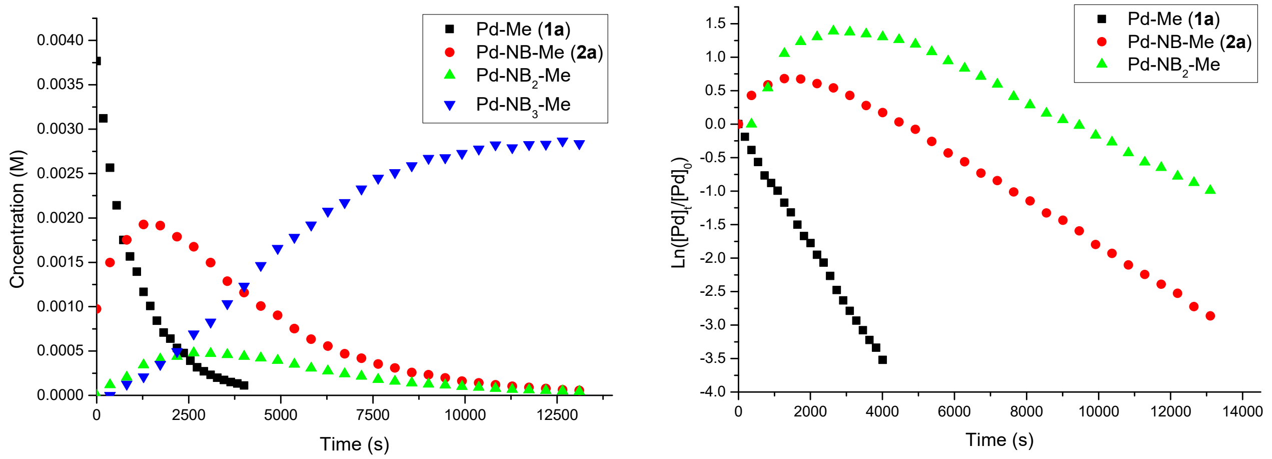

Figure S26. The relationship for **1a** and N-inserted products toward time (left), and the pseudo-first-order kinetic plot for the norbornene insertion of **1a** and N-inserted products (right) in $CDCl_3$ at 263 K.

Table S10. Kinetic data for successive norbornene insertion of **1a** in $CDCl_3$.

Observed rate	Rate constants for trans form isomers<br>Pseudo-first-order rate constant (s^{-1})
k_N^{trans}	8.69 x 10^{-4} (5.0)
k_{NN}^{trans}	3.32 x 10^{-4} (5.3)
k_{NNN}^{trans}	2.69 x 10^{-4} (3.5)

[a] Reaction condition: **1a** (5.3×10^{-3} M) and norbornene (9.9×10^{-2} M) in $CDCl_3$ (0.7 mL) at 263K.

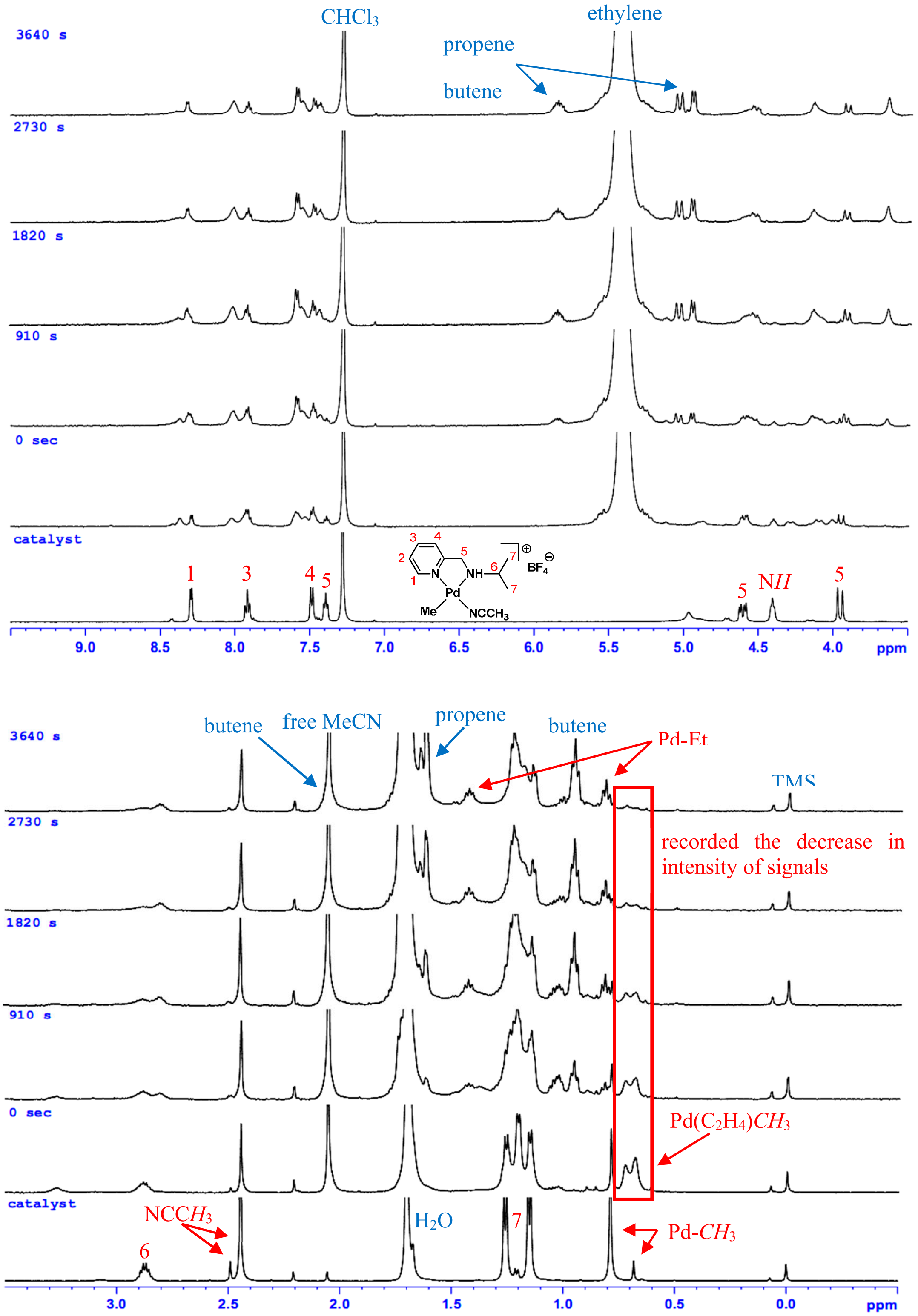

Figure S27. Ethylene insertion reaction for **1a** at 263 K with the range 3.5 - 9.5 ppm (up) and -0.5 - 3.5 ppm (down) in ^{1}H NMR spectra.

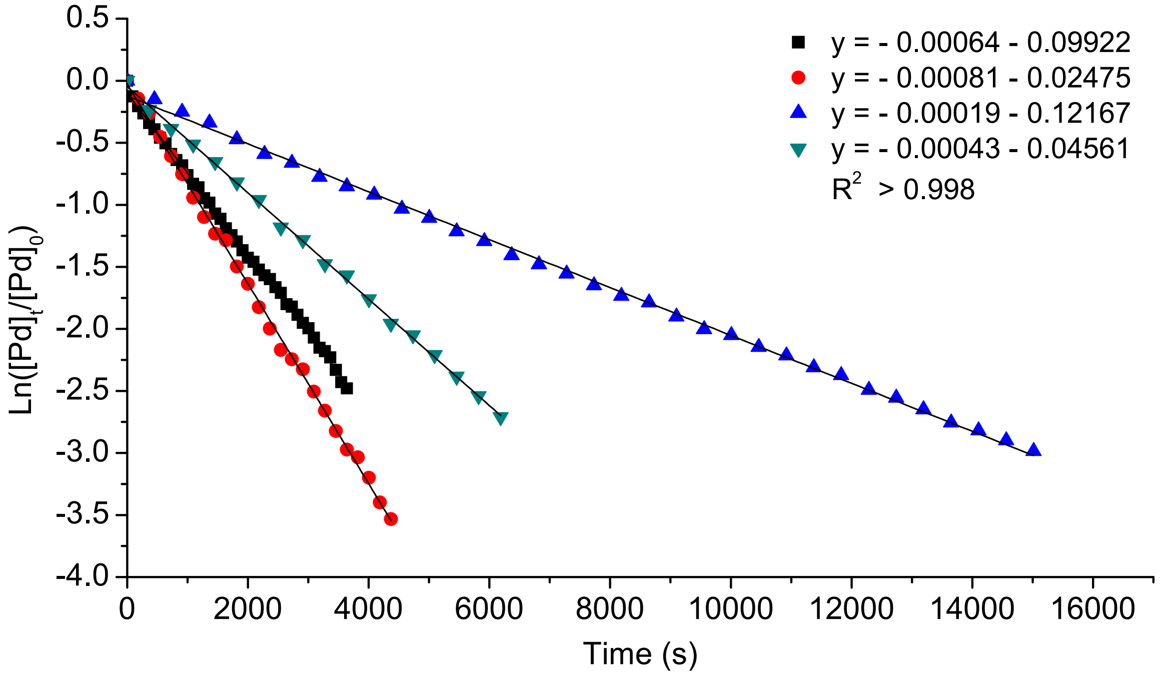

Figure S28. Pseudo-first-order kinetic plots for the ethylene insertion reactions of various catalysts at 263 K in $CDCl_3$ (■, *C*/*T*-**1a’**; ●, *T*-**1b’**; ▲, *C*/*T*-**1c’**; ▼, *C*/*T*-**1d’**).

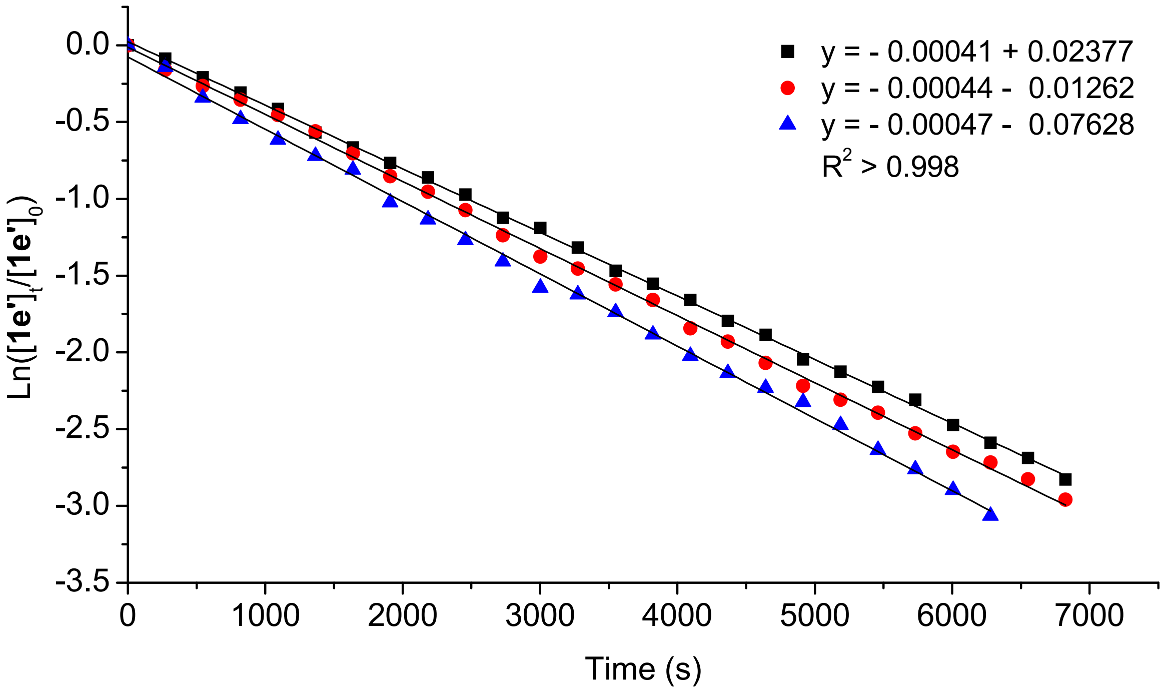

Figure S29. Pseudo-first-order kinetic plots for the norbornene insertion reactions of *C*/*T*-**1e’** with various [E]/[Pd] ratios at 263 K in $CDCl_3$ ([E]/[Pd]: ■, 14.3; ●, 25.0; ▲, 51.9).

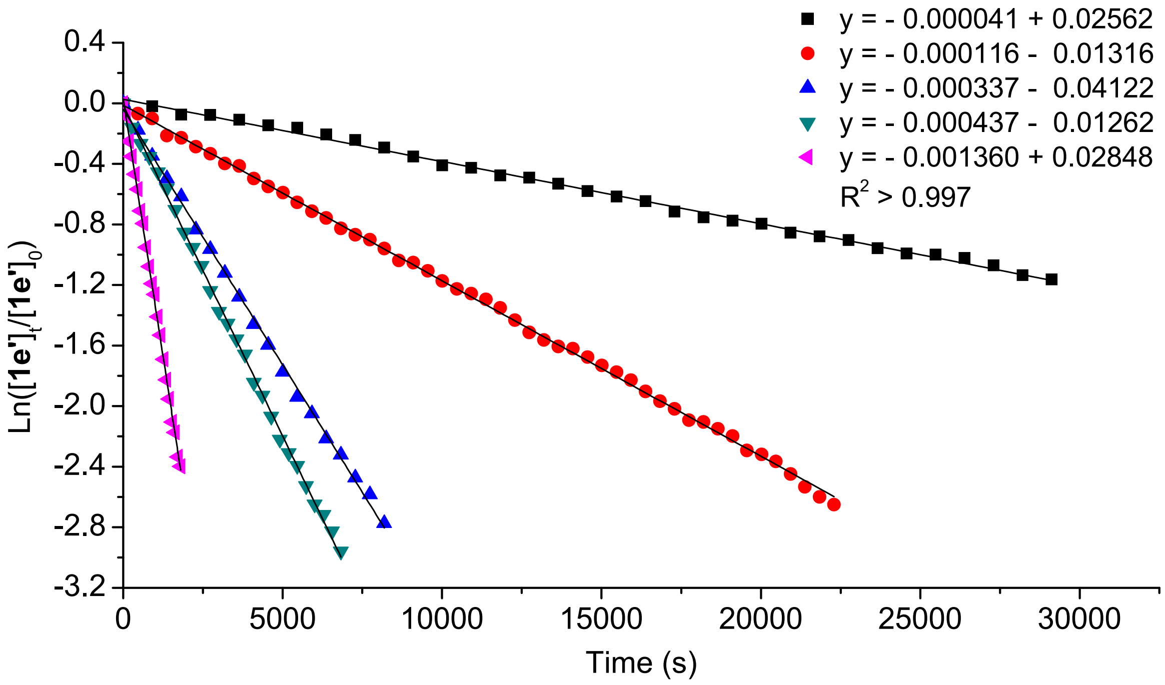

Figure S30. Pseudo-first-order kinetic plots for the norbornene insertion reactions of *C*/*T*-**1e'** at various temperatures in $CDCl_3$ (■, 243 K; ●, 253 K; ▲, 258 K; ▼, 263 K; ◀, 268 K).

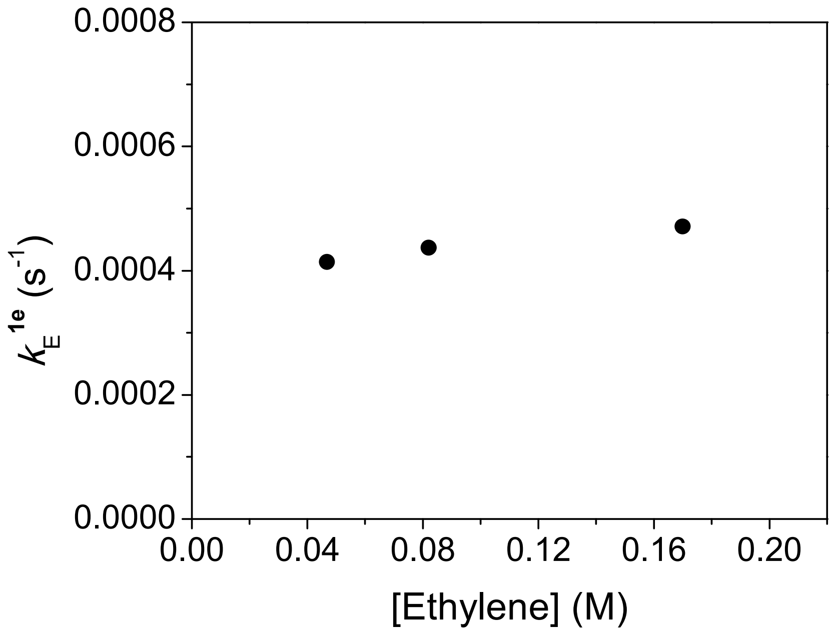

Figure S31. Relationship for k_E and [E] for **1e** at 263 K.

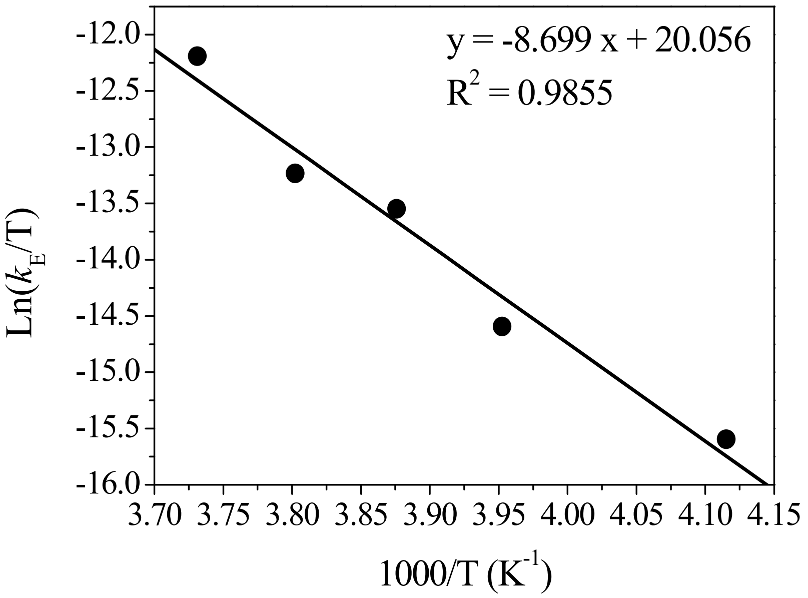

Figure S32. Eyring relationship for the ethylene insertion reaction of **1e** in $CDCl_3$.

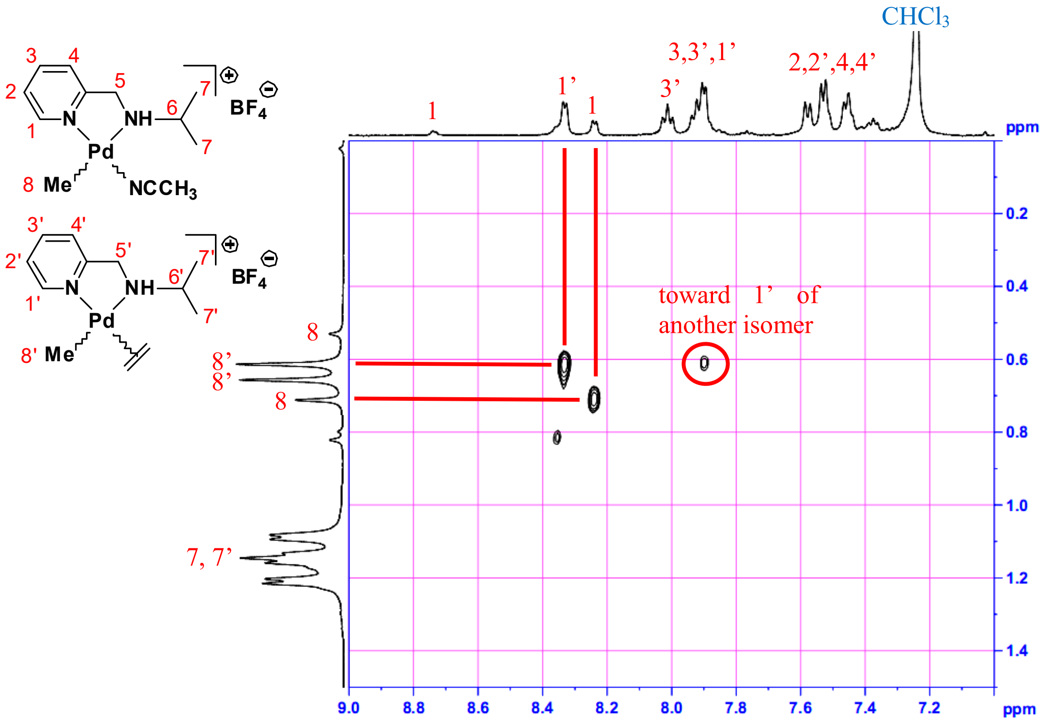

Figure S33. 2D NOESY spectrum of **1a'** at 223 K with small amount of **1a**.

The *T*/*C* ratio of ethylene-bound species **1e'**, in the formula of {[(2,6-$^{i}Pr_2C_6H_3$)$HNCH_2$(*o*-C_6H_4N)] Pd(Me)(CH_2=CH_2)}(BF_4), also changed along with temperature. The Van't Hoff plot of **1e'** as shown in Figure S32 gives the estimation of ΔH° for -0.73 kcal/mol and ΔS° for -1.72 cal/mol·K. At 298.15 K, the ΔG° is estimated as -0.22 kcal/mol, and the *trans*/*cis* percentages are 59/41.

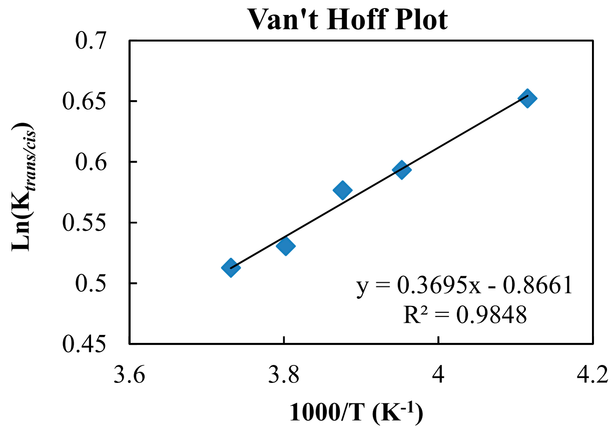

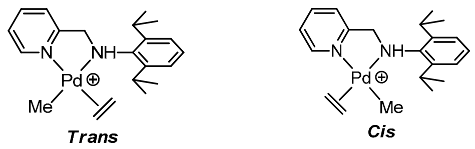

T	1000/T	$K_{T/C}$	Ln(K)
243	4.115226	1.92	0.652325
253	3.952569	1.81	0.593327
258	3.875969	1.78	0.576613
263	3.802281	1.70	0.530628
268	3.731343	1.67	0.512824

Figure S34. Van't Hoff plot and *T*/*C* ratios of **1e'**.

Theoretical Calculation

C-T isomerization

The first possible *C-T* isomerization is the ligand association-substitution reaction, involving square pyramidal intermediates, trigonal bipyramidal and square pyramidal transition states (Scheme S1).[11] Decrease of the distance for an external ethylene and palladium denoted as **6** and **6'**, respectively, affords the transition states **TS(6-6)** and **TS(6'-6')** in trigonal bipyramidal geometries, which the energies lie 14.1 and 7.0 kcal/mol above the **6** and **6'**, respectively (Scheme S2). Subsequent decrease of the ethylene-palladium distance resulted in the dissociation of original ethylene. Our trials to locate a ground state for **6** and **6'** with external ethylene in square pyramidal geometry resulted in a simple dissociation of ethylene. Relaxation of **TS(6-6)** and **TS(6'-6')** to the product sides give the **6** and **6'**, indicative of the ethylene exchange process. Such low ethylene exchange energies would result in the broadening signals in ^{1}H NMR spectra at low temperature, which are corresponded to the experimental observation. Therefore, the *C-T* isomerization via associative pathway has been ruled out.

Scheme S1. Possible *C-T* isomerization via Association-Substitution reaction (E + ZPE, kcal/mol). The GS1 and GS3 are not found. (R = 2,6-^{i}Pr$_2$C$_6$H$_3$)

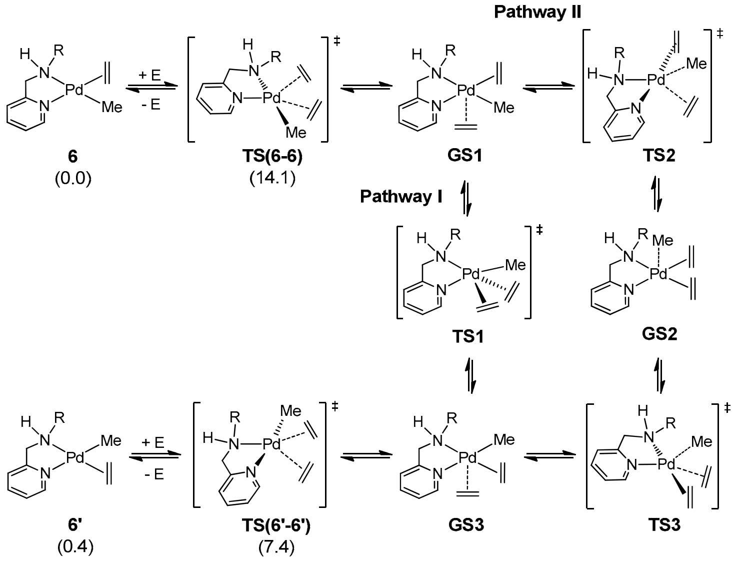

Scheme S2. Ethylene Exchange of [(Am-Py)Pd(Me)(C$_2$H$_4$)]$^+$ Complexes (E + ZPE, kcal/mol). (R = 2,6-^{i}Pr$_2$C$_6$H$_3$)

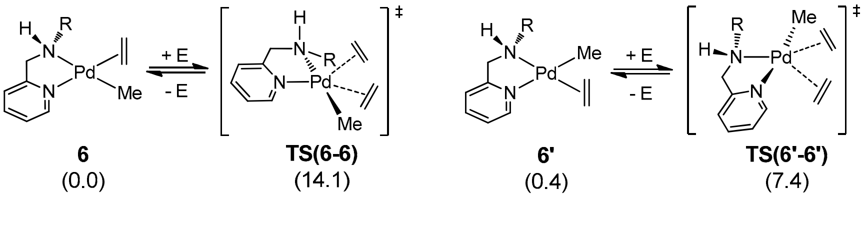

The second possible pathway is the ligand dissociation reaction. Increase of the ethylene-palladium distance could not find the local maximum or minimum. However, increase of the amine-palladium distance of **6** affords the transition states **TS(6-**

7) in trigonal pyramidal geometry, which is endothermic by 21.7 kcal/mol (Scheme S3). A weak δ-agostic interaction from ^{i}Pr group with the bond distance Pd-H = 2.5157 Å for **TS(6-7)** is found. The dissociative product **7** in trigonal bipyramidal geometry is lower in energy than **TS(6-7)** by 3.4 kcal/mol. The bond distances of Pd-H_{ax} and Pd-H_{eq} for **7** are 2.2027 and 2.5458 Å, respectively. Such value of energy barrier is comparable to the enthalpy of isomerization for norbornyl palladium complex (21.5 kcal/mol) in kinetic study.

Scheme S3. *C-T* Isomerization via Amine Dissociation (E + ZPE, kcal/mol). (R = 2,6-$^i Pr_2C_6H_3$)

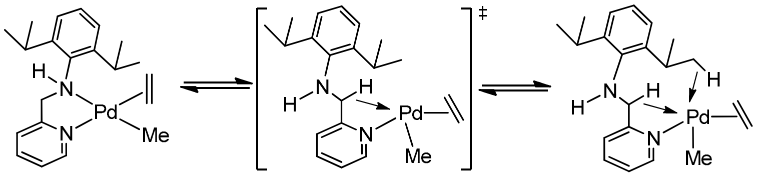

6 (0.0) **TS(6-7)** (21.7) **7** (18.3)

The transition states of *C-T* isomerization vis distorted tetrahedral geometry for norbornyl complex **8**, (Am-Py)Pd(C_7H_{10}Me)(C_2H_4), and γ-agostic complex **9'**, (am-py)Pd(C_7H_{10}Me), were also calculated (Scheme S4). The energies of **TS(8-8')** and **TS(9'-9)** lie 15.1 and 15.9 kcal/mol above the **8** and **9'**, respectively. The weak δ-agostic interactions of ^{i}Pr group for **TS(8-8')** and **TS(9'-9)** are observed with the bond distances Pd-H = 2.6270 and 2.2459 Å.

Scheme S4. *C-T* Isomerization via Distorted Tetrahedral Species for Norbornyl Complexes (E + ZPE, kcal/mol). (R = 2,6-$^i Pr_2C_6H_3$)

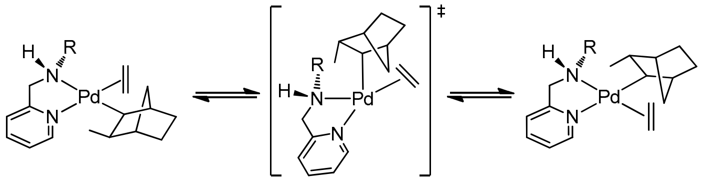

8 (0.0) **TS(8-8')** (15.1) **8'** (1.4)

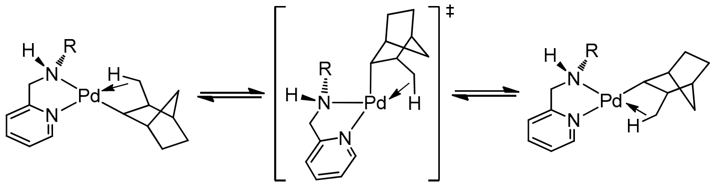

9' (0.0) **TS(9'-9)** (15.9) **9** (2.8)

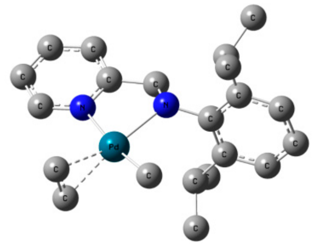

6

Pd	1.11139200	-0.61064400	-0.97525600
N	2.44698400	0.61481000	0.28751700
N	-0.36108500	0.62782800	0.07072200
C	-0.32342800	-1.51388300	-2.13655800
C	3.80067700	0.70783300	0.19140500
C	4.54583600	1.62515400	0.94388600
C	3.86338300	2.48956300	1.82094800
C	2.46466800	2.39714800	1.91711900
C	1.77973400	1.44186500	1.14434500
C	0.28349800	1.25106600	1.28895600
C	-1.73361400	0.14034600	0.34902900
C	-1.91721500	-1.03437300	1.13159200
C	-3.23956200	-1.44753400	1.39282800
C	-4.34056100	-0.74516300	0.88175600
C	-4.13449400	0.39661800	0.09743500
C	-2.83472500	0.87314700	-0.17810700
C	-0.76654700	-1.87965900	1.70017300
C	-0.85326100	-3.35872400	1.23701000
C	-0.72576000	-1.81356700	3.25226000
C	-2.70561800	2.14055900	-1.04190700
C	-3.16230800	1.87016800	-2.50167300
C	-3.48199900	3.33827300	-0.43194900
H	-1.20476500	-1.64521400	-1.50364300
H	0.01629800	-2.47669300	-2.53191900
H	-0.53390000	-0.81831400	-2.96003900
H	4.29183100	0.03303200	-0.50103500
H	5.62474600	1.66183200	0.83896200
H	4.40783900	3.21917300	2.41265200
H	1.91151400	3.05142200	2.58409300
H	0.08863200	0.59058600	2.13738100
H	-3.41020900	-2.33598900	1.99413200
H	-5.34999300	-1.08796100	1.09054600
H	-4.99300500	0.93075300	-0.29896300
H	0.18834500	-1.49099000	1.31889000
H	-0.86679000	-3.43941800	0.14401800
H	-1.75654500	-3.84788900	1.61974800
H	0.01026900	-3.92211700	1.61355400
H	0.13340900	-2.37730700	3.63705800
H	-1.63306000	-2.25167500	3.68529300
H	-0.65521900	-0.78354300	3.62698100
H	-1.65502000	2.47057600	-1.09862100
H	-3.03529200	2.77045700	-3.11556900
H	-4.22162600	1.58906500	-2.53337900
H	-2.58866700	1.05478400	-2.96060700
H	-3.16176800	3.54537500	0.59687500
H	-4.56111900	3.14737500	-0.41247600
H	-3.31726200	4.24094100	-1.03317400
H	-0.43750100	1.35737300	-0.64454800
H	-0.20371500	2.20864000	1.51558800
C	2.59830000	-1.20812600	-2.56484000
C	2.61857300	-2.17041900	-1.55692500
H	2.06805200	-3.10086000	-1.65956600
H	3.35836400	-2.14416700	-0.76007500
H	2.02709200	-1.36474200	-3.47479400
H	3.32031200	-0.39511900	-2.59393700

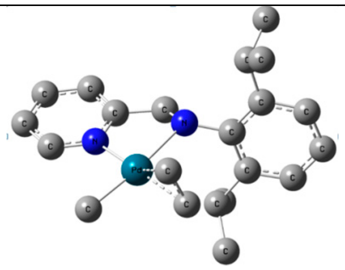

6’

Pd(Fragment=1)	1.18155100	-0.58244400	-1.06098300
C(Fragment=1)	2.69816600	-1.56998900	-2.02954400
N(Fragment=1)	2.52249400	0.49469300	0.20439200
N(Fragment=1)	-0.29420100	0.64581700	0.15099300
C(Fragment=2)	-0.05003000	-0.79871400	-2.94394700
C(Fragment=1)	3.87535000	0.54278500	0.04962500
C(Fragment=1)	4.68794100	1.37108200	0.83235100
C(Fragment=1)	4.08480600	2.19058800	1.80430200
C(Fragment=1)	2.68984900	2.14426000	1.95927500
C(Fragment=1)	1.92628500	1.28087600	1.15352600
C(Fragment=1)	0.43045100	1.15748200	1.36570900
C(Fragment=1)	-1.65972700	0.15338800	0.42922000
C(Fragment=1)	-1.84255900	-1.06175300	1.15378700
C(Fragment=1)	-3.16383600	-1.48619200	1.40393400
C(Fragment=1)	-4.26946300	-0.75879200	0.93995700
C(Fragment=1)	-4.06804200	0.42344600	0.21749600
C(Fragment=1)	-2.76996300	0.91236700	-0.04491400
C(Fragment=1)	-0.69119900	-1.92570500	1.69682900
C(Fragment=1)	-0.80463300	-3.41123800	1.26116900
C(Fragment=1)	-0.61783000	-1.85245300	3.24894500
C(Fragment=1)	-2.65604700	2.24601300	-0.80638600
C(Fragment=1)	-3.29637300	2.15936600	-2.21756400
C(Fragment=1)	-3.27710000	3.41168400	0.01122400
C(Fragment=2)	-0.34258900	-1.87535300	-2.11164500
H(Fragment=2)	-0.68921800	0.08156500	-2.96273200
H(Fragment=2)	0.66464500	-0.88532900	-3.75718700
H(Fragment=1)	4.30287700	-0.09258000	-0.71427200
H(Fragment=1)	5.76098900	1.37381400	0.67517300
H(Fragment=1)	4.68541900	2.85109200	2.42226000
H(Fragment=1)	2.19439400	2.76600100	2.69861300
H(Fragment=1)	0.24735600	0.46046500	2.18614700
H(Fragment=1)	-3.33047600	-2.40272600	1.96250600
H(Fragment=1)	-5.27721800	-1.11114100	1.14060200
H(Fragment=1)	-4.92945100	0.98213100	-0.13705900
H(Fragment=1)	0.25819800	-1.55372200	1.28778200
H(Fragment=1)	-0.85442600	-3.51774700	0.17199900
H(Fragment=1)	-1.69559700	-3.88961500	1.68394000
H(Fragment=1)	0.06828200	-3.97276000	1.61747900
H(Fragment=1)	0.24548100	-2.41957600	3.61922400
H(Fragment=1)	-1.51979400	-2.28484500	3.69881100
H(Fragment=1)	-0.53733200	-0.82276900	3.62169100
H(Fragment=1)	-1.60046600	2.51992000	-0.96462500
H(Fragment=1)	-3.16029200	3.10447400	-2.75753600
H(Fragment=1)	-4.37324000	1.96545800	-2.15364500
H(Fragment=1)	-2.85170100	1.35356800	-2.81649100
H(Fragment=1)	-2.81231500	3.50147000	1.00129700
H(Fragment=1)	-4.35209000	3.25888500	0.16263900
H(Fragment=1)	-3.14601700	4.36285100	-0.51944100
H(Fragment=1)	-0.37176300	1.42261600	-0.50990800
H(Fragment=1)	0.01798400	2.12599300	1.68040100
H(Fragment=2)	-1.21417500	-1.85033300	-1.46403800
H(Fragment=2)	0.12812600	-2.84365400	-2.25819300
H(Fragment=1)	2.32163900	-2.32386500	-2.72637700
H(Fragment=1)	3.28710500	-2.05328200	-1.23995000
H(Fragment=1)	3.28295700	-0.81641900	-2.57170100

References

1. Sheldrick, G. M. *SHELXTL-97*, *Program for Crystal Structure Solution*; University of Göttingen: Germany, 1997.
2. Cromer, D. T.; Waber, J. T. *International Tables for X-ray Crystallography*, vol. IV; Kynoch Press: Birmingham, England, 1974.
3. Frisch, M. J.; et al. *Gaussian 09*, Revision A.1; Gaussian, Inc.: Wallingford, CT, 2009.
4. (a) Becke, A. D. *J. Chem. Phys.* **1993**, *98*, 5648-5652. (b) Lee, C.; Yang, W.; Parr, R. G. *Phys. Rev. B* **1988**, *37*, 785-789.
5. (a) Dunning Jr., T. H.; Hay, P. J. In *Modern Theoretical Chemistry*; Schaefer III, H. F. Ed.; Plenum: New York, 1976; Vol. 3, pp 1-28. (b) Hay, P. J.; Wadt, W. R. *J. Chem. Phys.* **1985**, *82*, 270-83. (c) Wadt, W. R.; Hay, P. J. *J. Chem. Phys.* **1985**, *82*, 284-98. (d) Hay, P. J.; Wadt, W. R. *J. Chem. Phys.* **1985**, *82*, 299-310.
6. (a) Boys, S. F.; Bernardi, F. *Mol. Phys.* **1970**, *19*, 553-566. (b) Simon, S.; Duran, M.; Dannenberg, J. J. *J. Chem. Phys.* **1996**, *105*, 11024-11031.
7. (a) Gonzalez, C.; Schlegel, H. B. *J. Chem. Phys*. **1989**, *90*, 2154-2161. (b) Gonzalez, C.; Schlegel, H. B. *J. Phys. Chem*. **1990**, *94*, 5523-5527.
8. (a) Fineman, M.; Ross, S. D. *J. Polym. Sci.* **1950**, 5, 259-265. (b) Stevens, M. P. *Polymer Chemistry: An Introduction*, 3rd Edition; Oxford University Press: New York, **1999**, p194.
9. Kiesewetter, J.; Kaminsky, W. *Chem. Eur. J.* **2003**, *9*, 1750-1758.
10. (a) Anderson, G. K.; Cross, R. *J. Chem. Soc. Rev.* **1980**, *9*, 185-215. (b) Casares, J. A.; Espinet, P. *Inorg. Chem.* **1997**, *36*, 5428-5431. (c) Casares, J. A.; Espinet, P. *Inorg. Chem.* **1998**, *37*, 2096. (d) Noda, S; Nakamura, A.; Kochi, T.; Chung, L. W.; Morokuma, K.; Nozaki, K. *J. Am. Chem. Soc.* **2009**, *131*, 14088-14100.
